# Uncovering the pharmacological mechanism of the effects of the Banxia-Xiakucao Chinese Herb Pair on sleep disorder by a systems pharmacology approach

**DOI:** 10.1038/s41598-020-77431-1

**Published:** 2020-11-24

**Authors:** Jing Guo, Meng-Ping Lou, Lin-Lin Hu, Xin Zhang

**Affiliations:** 1 First Clincal Medical College, Zhejiang Chinese Medical University, 548 Binwen Road, Hangzhou, 310053 Zhejiang People’s Republic of China; 2 Guangxing Affiliated Hospital of Zhejiang Chinese Medical University, 453 Tiyuchang Road, Hangzhou, 310007 Zhejiang People’s Republic of China; 3 College of Pharmaceutical Sciences, Zhejiang Chinese Medical University, 548 Binwen Road, Hangzhou, 310053 Zhejiang People’s Republic of China

**Keywords:** Sleep disorders, Computational biology and bioinformatics, Neuroscience, Systems biology

## Abstract

Sleep disorder (SD) has a high incidence and seriously affects quality of life, mental health and even the manifestation of physical diseases. The combination of *Pinellia ternata* (Chinese name: banxia) and *Prunella vulgaris* (Chinese name: xiakucao), known as the Banxia–Xiakucao Chinese herb pair (BXHP), is a proven Chinese herbal medicine that has been used to treat SD for thousands of years due to its significant clinical effects. However, its active pharmacological components and sedative–hypnotic mechanisms have not been fully elucidated. Thus, the present study used a systematic pharmacological approach to develop pharmacokinetic screens and target predictions via construction of a protein–protein interaction network and annotation database for SD-related and putative BXHP-related targets. Visualization, screening and integrated discovery enrichment analyses were conducted. The BXHP chemical database contains 166 compounds between the two herbal ingredients, and of these, 22 potential active molecules were screened by pharmacokinetic evaluation. The targets of 114 of the active molecules were predicted, and 34 were selected for further analysis. Finally, gene ontology and Kyoto Encyclopedia of Genes and Genomes pathway analyses suggested that BXHP can reduce inflammatory responses. and mediate immune-related and central nervous system neurotransmitters via regulation of multiple targets and pathways. The use of a systematic pharmacology-based approach in the present study further elucidated the mechanisms of action underlying BXHP for the treatment of SD from a holistic perspective and sheds light on the systemic mechanisms of action of Chinese herbal medicines in general.

## Introduction

Sleep disorder (SD) is common in the general population as well as in individuals with mental illnesses. People with SD often report dissatisfaction regarding sleep quality, sleep time and amount of sleep^1^, which can adversely affect daily functions^2^ and quality of life^3^. Epidemiological data from Western populations indicate that the prevalence of various SDs in the general population ranges from 0.047 to 50.5%^4^, and that the most common SD is insomnia followed by sleep apnoea^5^, restlessness syndrome^6^, nightmares, sleep talking, sleepwalking^7^ and narcolepsy^8^. Long-term SDs cause anxiety, depression and/or fear and can lead to decreased mental activity^9^ and increased risks of cardiovascular disease, dementia^10^, mental illness and hypogonadism^11^.

Sedative–hypnotic drugs, including benzodiazepines and non-benzodiazepines, are commonly used to treat SD but are not suitable for prolonged use^12^. In fact, patients may not be able to tolerate the various side effects of these drugs, which include rebound withdrawal effects, disturbed sleep structure, drowsiness, memory disorders and bad behaviors during sleep^13,14^. In addition to the abovementioned drugs, antidepressants, antipsychotics and antihistamines can also be used to treat SD due to their sedative-hypnotic effects. However, these treatments are generally not recommended when there are no corresponding symptoms of a disease^15^. Due to limitations in the current treatment options for SD, a significant number of patients worldwide have begun to use herbal medicines as an adjuvant therapy for SD^16^.

**Figure 1:**
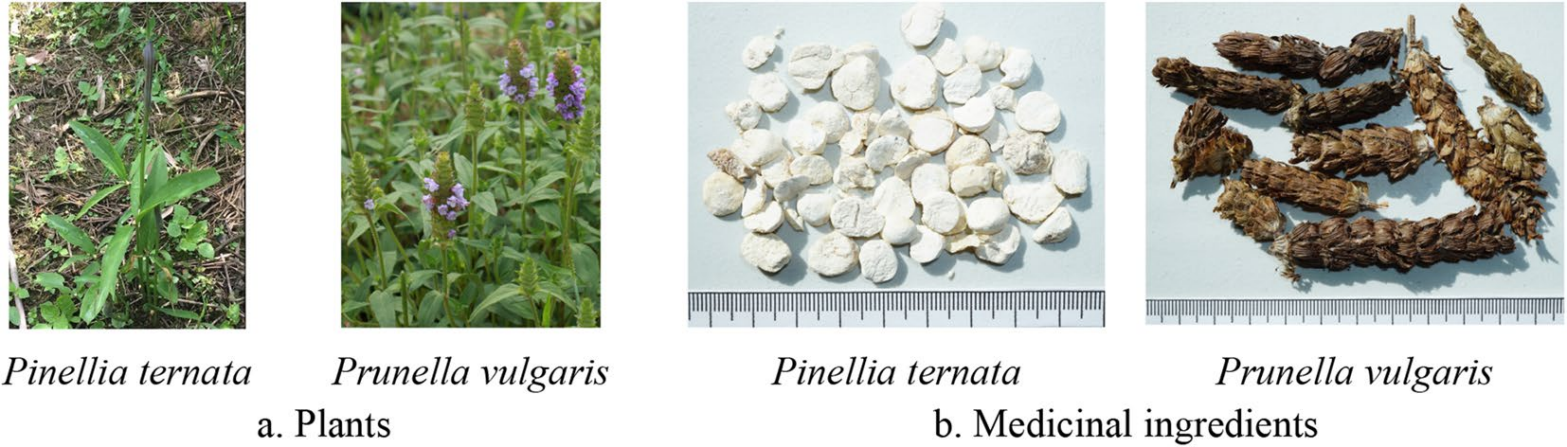
Photographs of *Pinellia ternata* (*Pinellia ternata* (Thunb.) Breit.) and *Prunella vulgaris* (*Prunella vulgaris* L.) in plant and medicinal ingredient forms.

It has been shown that Chinese herbal medicine (CHM) therapies are reliable and safe for the treatment of SD^17^. CHM involves mixtures of complex compounds that are thought to interact with various biological targets to exert multiple therapeutic effects. Chinese herb pairs are the simplest form and most concentrated representative of Chinese herbal compatibility and inherently convey the basic ideas underlying ​​traditional Chinese medicine (TCM) prescriptions. Due to their gentle therapeutic actions, mild side effects and ability to treat complex chronic diseases, these pairs have received widespread attention^18,19^. For example, the Banxia–Xiakucao Chinese herb pair (BXHP) is an empirical formula based on the Banxia decoction, which contains *Pinellia ternate* (Banxia) and *Prunella vulgaris* (Xiakucao) as its two ingredients (Fig. 1) and was recorded in *The Medical Classic of the Yellow Emperor* more than 2000 years ago. Various extracts of *Pinellia ternata* and *Prunella vulgaris* have been shown to exert different sedative and hypnotic effects^20,21^. For example, BXHP increases the expression of the serotonin 5-hydroxytryptamine receptors 5-HT_1A_ and 5-HT_2A_ in the hypothalamus and reduces the serum levels of interleukin (IL)-1 and tumor necrosis factor (TNF)-α^22^. Additionally, *Prunella vulgaris* exerts a variety of biological activities that include antioxidant, cytoprotective, anti-proliferative, anti-inflammatory and sedative–hypnotic effects. Although the effects of this herb pair may be the achieved via the mechanisms associated with the prevention and treatment of SD complications, the active substances and pharmacological mechanisms of BXHP have yet to be evaluated comprehensively.

**Figure 2:**
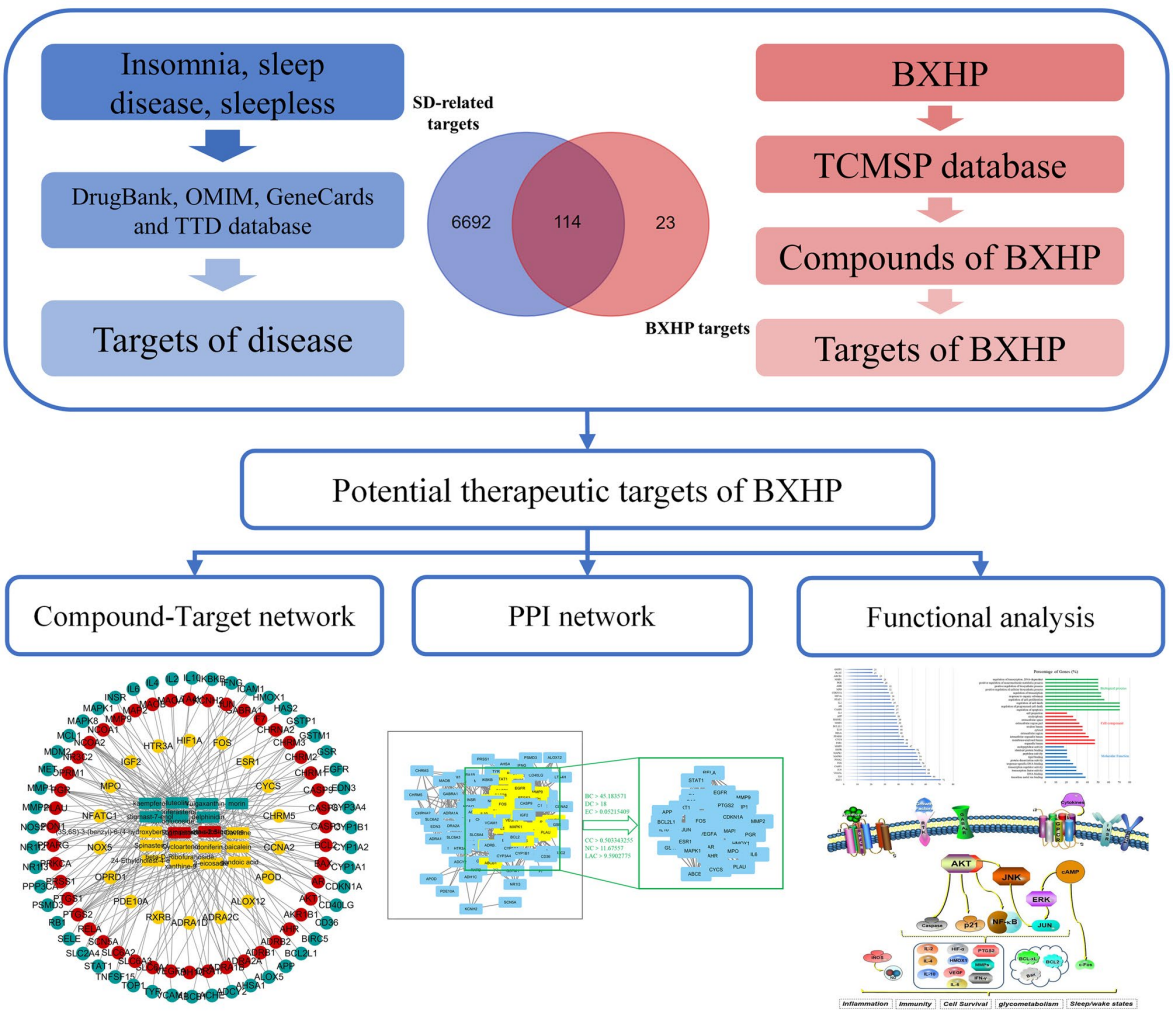
Flowchart of this systems pharmacology-based study exploring the mechanisms of action of Banxia-Xiakucao Chinese Herb Pair (BXHP) for treating sleep disorder. OMIM, Online Mendelian Inheritance in Man; TTD, Therapeutic Target Database; TCMSP, Traditional Chinese Medicine Systems Pharmacology; PPI, protein–protein interaction.

Because herbal formulas contain multiple ingredients with multidimensional pharmacological effects, it is challenging to determine the specific roles of these individual ingredients using traditional analytical methods. However, the emerging science of systems pharmacology has changed the traditional model of thinking that employs the framework of "one drug, one target, one disease"^23^ because of its unique combination of systems biology, multidirectional pharmacology, computational biology and analyses of networks that allow exploration of the connections among drugs and diseases using a holistic perspective^24^. The present study employed a systematic pharmacological approach to advance the discovery and understanding of the mechanisms underlying effective BXHP treatment of SD. A brief flowchart of the study design is provided in Fig. 2.

## Results

### Screening of the active compounds in BXHP

**Figure 3:**
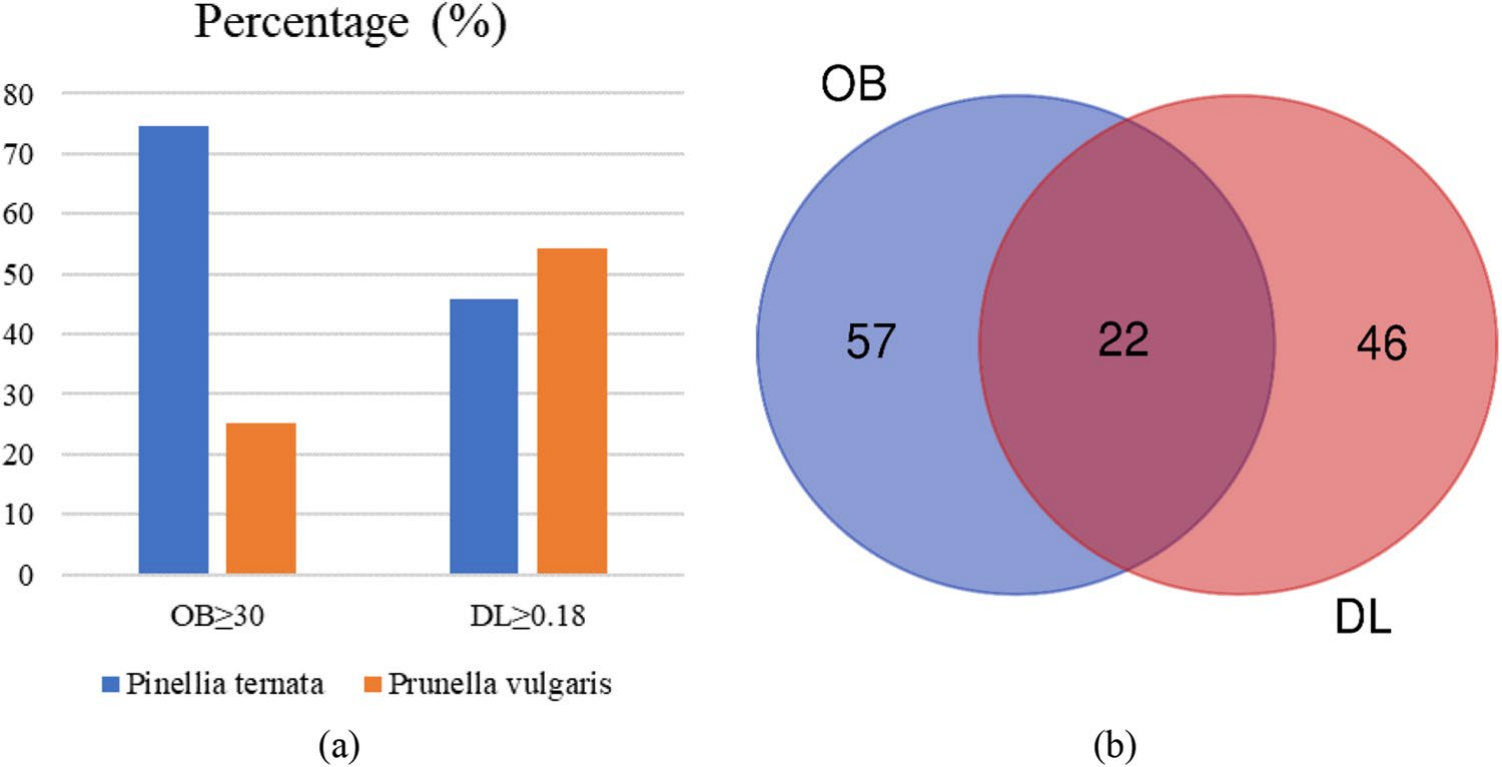
Active compounds in Banxia-Xiakucao Chinese Herb Pair. (**a**) and (**b**) Absorption, distribution, metabolism, and excretion (ADME) data for *Pinellia ternate* (Banxia) and *Prunella vulgaris* (Xiakucao). OB, oral bioavailability; DL, drug-likeness.

According to the TCM systems pharmacology database and analysis platform (TCMSP), 116 and 60 compounds from *Pinellia ternata* and *Prunella vulgaris*, respectively, are present in BXHP (Supplementary Table S1). The pharmacokinetic parameters systemic bioavailability and distribution after oral absorption (i.e. oral bioavailability [OB]) and similarly structural compounds and drugs for clinical use in the DrugBank database (i.e. drug-likeness [DL])^25^, which are related to the absorption, distribution, metabolism and excretion (ADME) of these active compounds, were screened using the following criteria: OB ≥ 30% and DL ≥ 0.18^26^. As a result, 47.6% (79/166) and 41.0% (68/166) of the compounds had good OB and DL values, respectively. A total of 22 duplicate compounds were included (Fig. 3a,b). *Pinellia ternata* and *Prunella vulgaris* were found to contain 13 and 11 compounds, respectively, including 2 common compounds (beta-sitosterol and stigmasterol). (Supplementary Table S2).

### Potential therapeutic targets of BXHP

A total of 137 predicted targets of 19 candidate compounds were identified (Supplementary Table S3); 3 candidate compounds did not have corresponding targets. The TCMSP was used to predict the pharmacological targets of BXHP candidate compounds, and gene names were extracted from UniProt (https://www.uniprot.org/).

### Identification of SD-related targets

**Figure 4:**
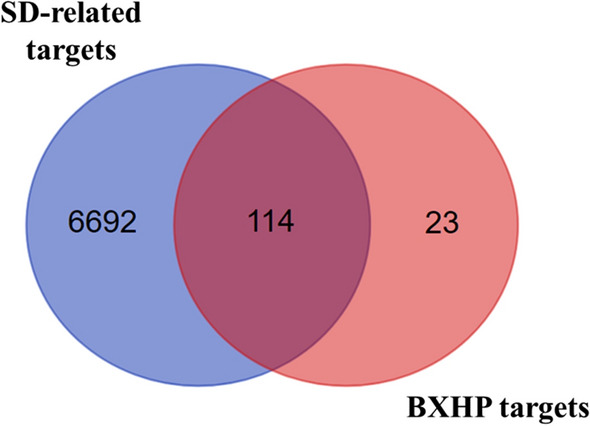
The verified the therapeutic potential of Banxia-Xiakucao Chinese Herb Pair (BXHP) was constructed by linking the overlapped targets (between BXHP putative and known SD-related). SD: Sleep Disorder.

As the pharmacological effects of a drug determine its indications, we searched various databases, including DrugBank, Online Mendelian Inheritance in Man (OMIM), GeneCards and Therapeutic Target Database (TTD), to identify 6806 SD-related targets. After removing redundant data, SD-related targets were identified (Supplementary Table S4) and included 114 of the BXHP targets (Fig. 4), which verified the therapeutic potential of BXHP as a sedative-hypnotic TCM formulation. The top six active ingredients of BXHP, in descending order, were behenyl alcohol, luteolin, beta-sitosterol, stigmasterol, baicalein and cavidine.

### Generation of a composite target network for BXHP

**Figure 5:**
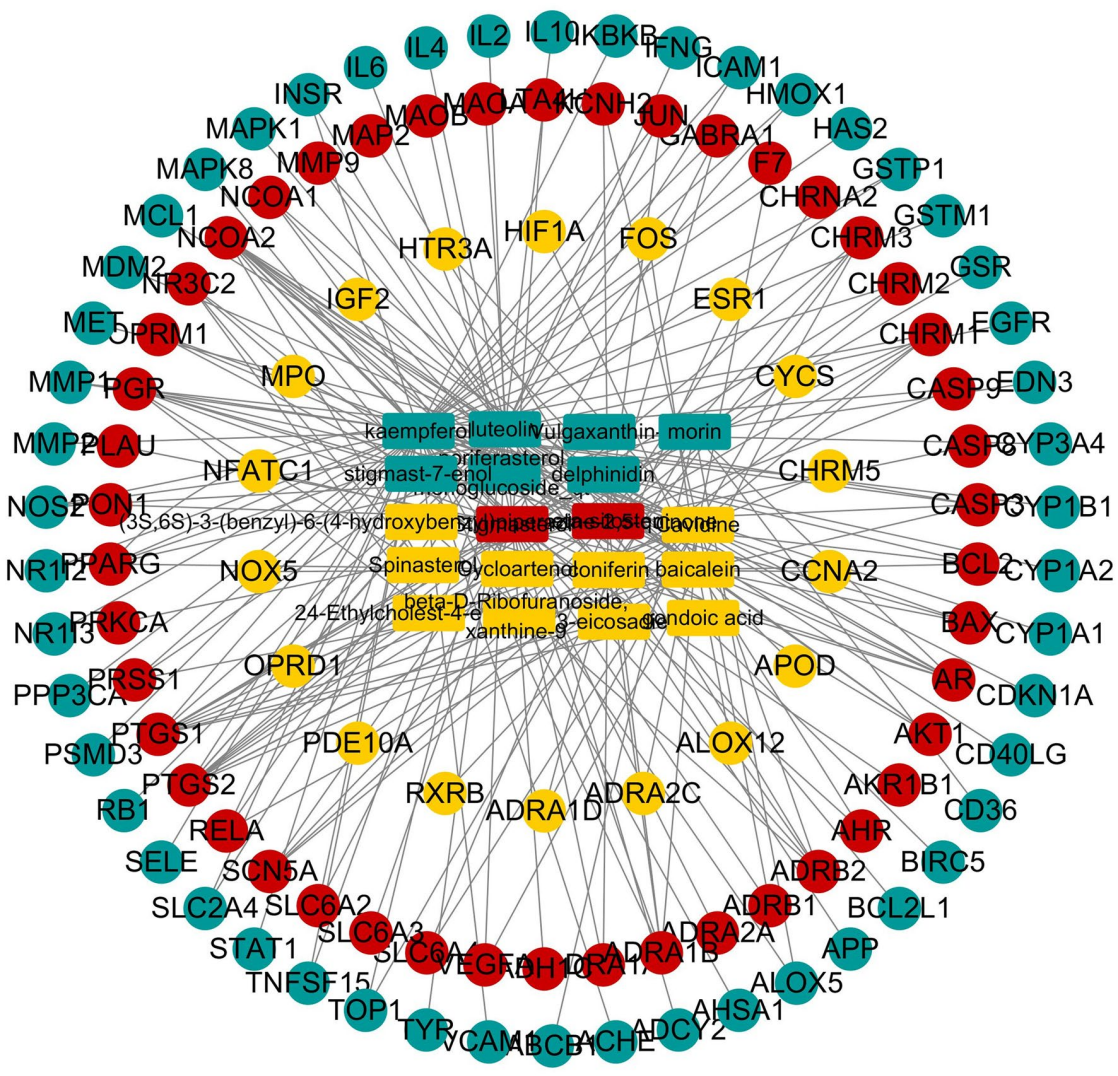
Compound-target network constructed by linking candidate compounds of Banxia-Xiakucao Chinese Herb Pair with their potential targets. In total, 19 candidate compounds of Banxia-Xiakucao Chinese Herb Pair [yellow rectangle, *Pinellia ternate* (Banxia); blue rectangle, *Prunella vulgaris* (Xiakucao); red rectangle, *Pinellia ternate* (Banxia) and *Prunella vulgaris* (Xiakucao)] were connected to 114 putative sleep disorder-related targets [yellow circles, *Pinellia ternate* (Banxia); blue circles, *Prunella vulgaris* (Xiakucao); red circles, *Pinellia ternate* (Banxia) and *Prunella vulgaris* (Xiakucao)].

All available chemical, genomic and pharmacological information was integrated to evaluate the 114 putative SD-related targets of the 19 candidate BXHP compounds^27^. Although each herb had a different number of targets, the targets clearly overlapped, suggesting synergistic effects. To further understand the complex interactions among the compounds and corresponding targets at a systems level, a composite target network was constructed based on the candidate BXHP compounds and their potential targets (Fig. 5); the network contained 133 nodes and 227 edges.

A network topology analysis produced the following results: network concentration = 0.328; network density = 0.026; network heterogeneity = 1.897; and shortest path = 17,556 (100%). The average degree of the nodes was 3.414, and 22 nodes had a larger than average degree. The average betweenness centrality of the nodes was 0.016392473, and 18 nodes had greater betweenness centrality than the average. Detailed data about these composite target networks are shown in Table S5. In the network topology analysis, the degree of a node represented the number of edges associated with it. Among them, 9 candidate compounds possessed a degree value higher than 3.414, suggesting that most of the compounds act on multiple targets and potentially explaining the pleiotropic effects of the active compounds in BXHP. For example, anisodamine, luteolin and beta-sitosterol had 47, 40 and 25 targets, respectively.

### Protein–protein interaction (PPI) networks

Genes and proteins do not operate independently but rather work at multiple levels via interconnected molecular networks and pathways^28^. PPI networks reflect the behaviour and properties of biomolecules and are particularly useful for understanding the roles of various proteins in complex diseases, including SD. STRING version 11.0 was employed to evaluate PPI data using a species limit of "Homo sapiens" and confidence score of ≥ 0.4. Node 1, Node 2 and the combined scores were imported into Cytoscape 3.7.0 software to construct 114 BXHP candidate targets with 114 nodes and 1,290 edges. Using the Cytoscape plugin CytoNCA, the following six topological features were selected to identify candidate targets: betweenness centrality (BC), degree centrality (DC), closeness centrality (CC), eigenvector centrality (EC), network centrality (NC) and local average connectivity (LAC)^29^. These features had median values of 45.183571, 18, 0.05215409, 0.503343255, 11.67557 and 9.5902775, respectively. Subsequently, 34 candidate targets with higher than the median values of these topological features were identified.

**Figure 6:**
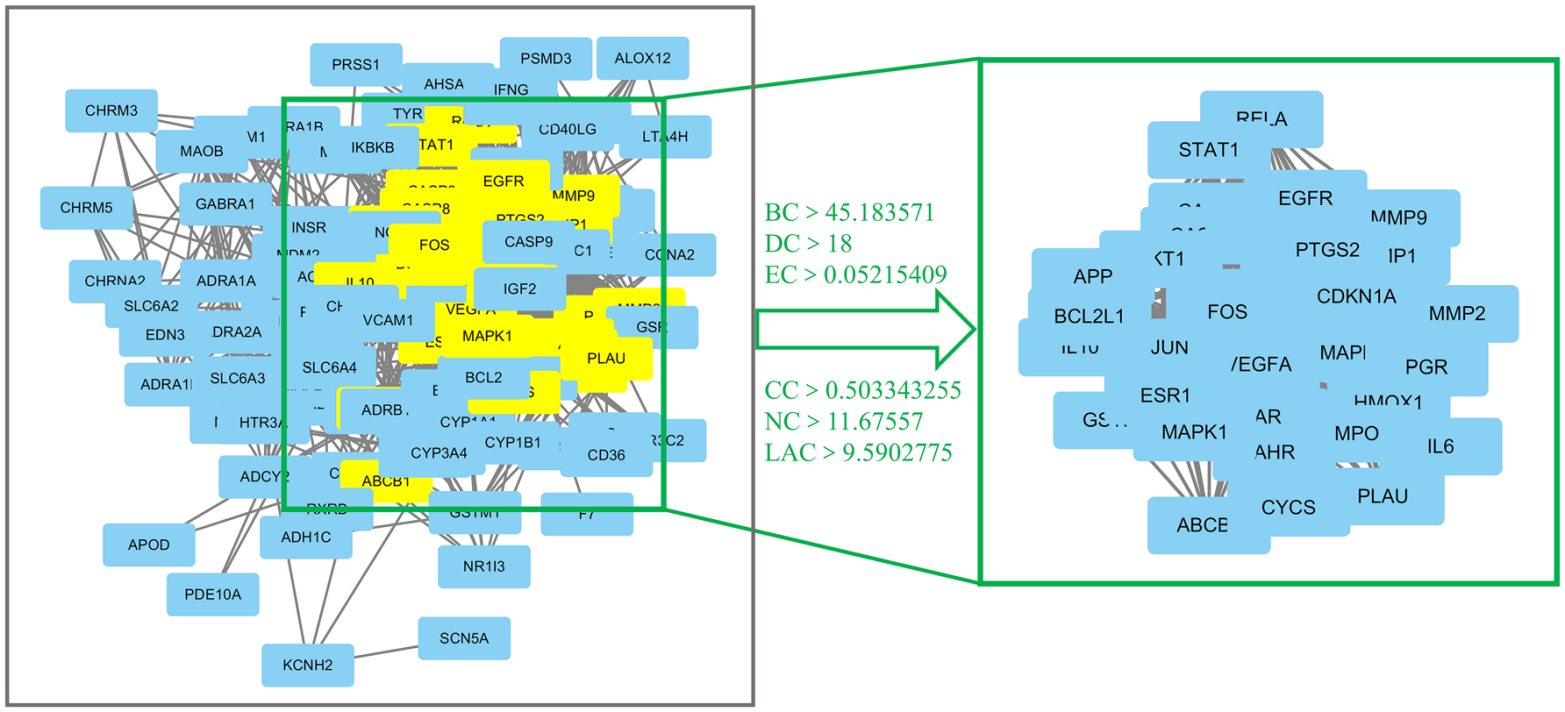
Identification of candidate targets for Banxia-Xiakucao Chinese Herb Pair in the treatment of sleep disorder. Flow chart of the screening process for the PPI network. Identification of candidate BXHP targets for sleep disorder treatment through the PPI network. Thirty-four core candidate BXHP targets with median topological feature values above the median were identified. BC, betweenness centrality; DC, degree centrality; EC, eigenvector centrality; CC, closeness centrality; NC, network centrality; LAC, local average connectivity.

**Figure 7:**
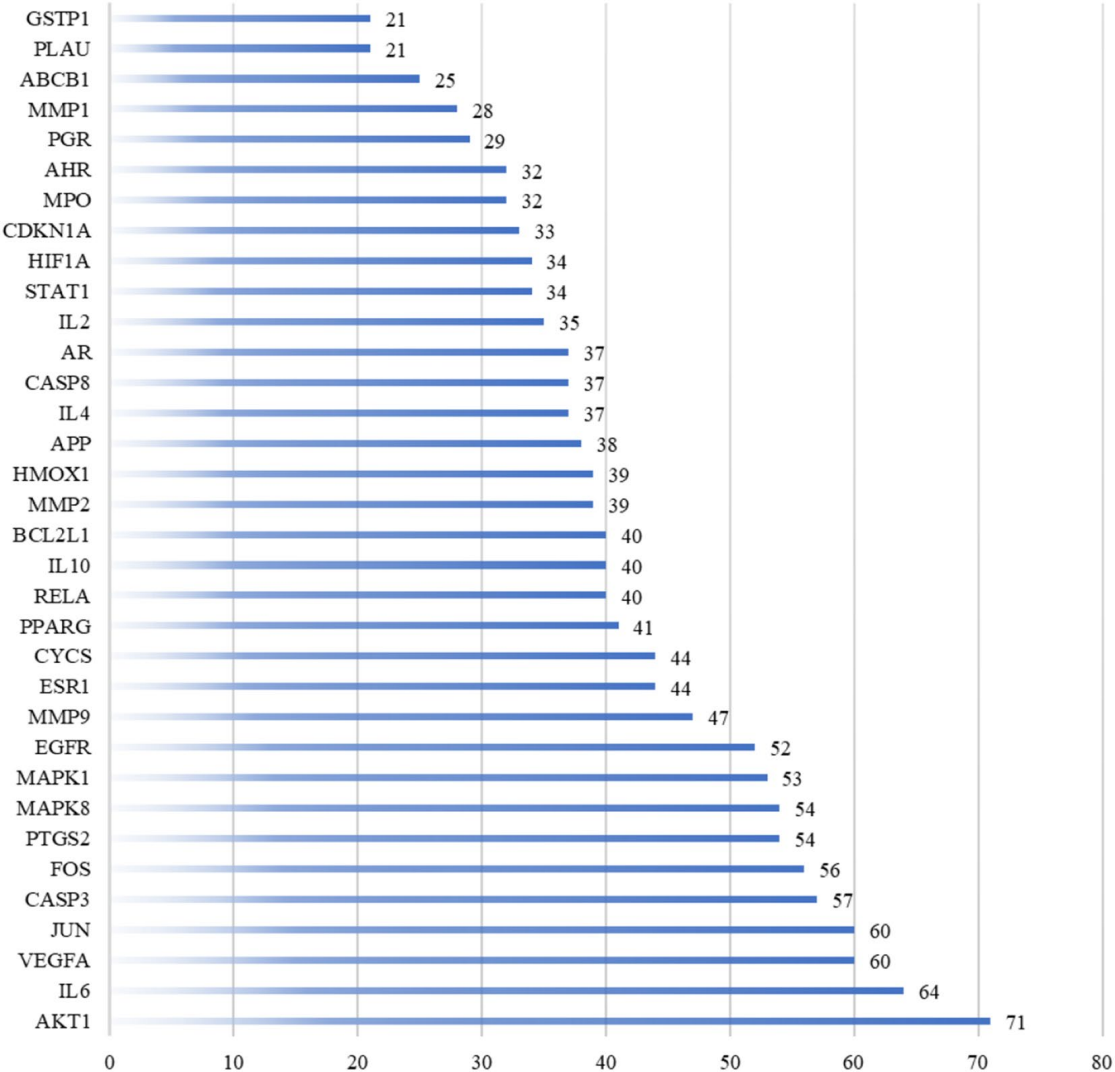
Degree centrality values of the core candidate Banxia-Xiakucao Chinese Herb Pair targets.

A representative flowchart of the screening process is provided in Fig. 6. The detailed topological characteristics of the core PPI network and the 34 candidate targets are shown in Table S6. The degree of the nodes was larger than average for 34 key protein nodes (Fig. 6). The degree for Akt1 (71), IL-6 (64), vascular endothelial growth factor A (VEGFA) (60), JUN (60) and caspase 3 (CASP3) (57) was higher than those of the other protein nodes, suggesting that these five protein networks play important roles in the network, serving as bridges to other nodes (Fig. 7).

### Pathway enrichment analysis of the candidate BXHP targets

**Figure 8:**
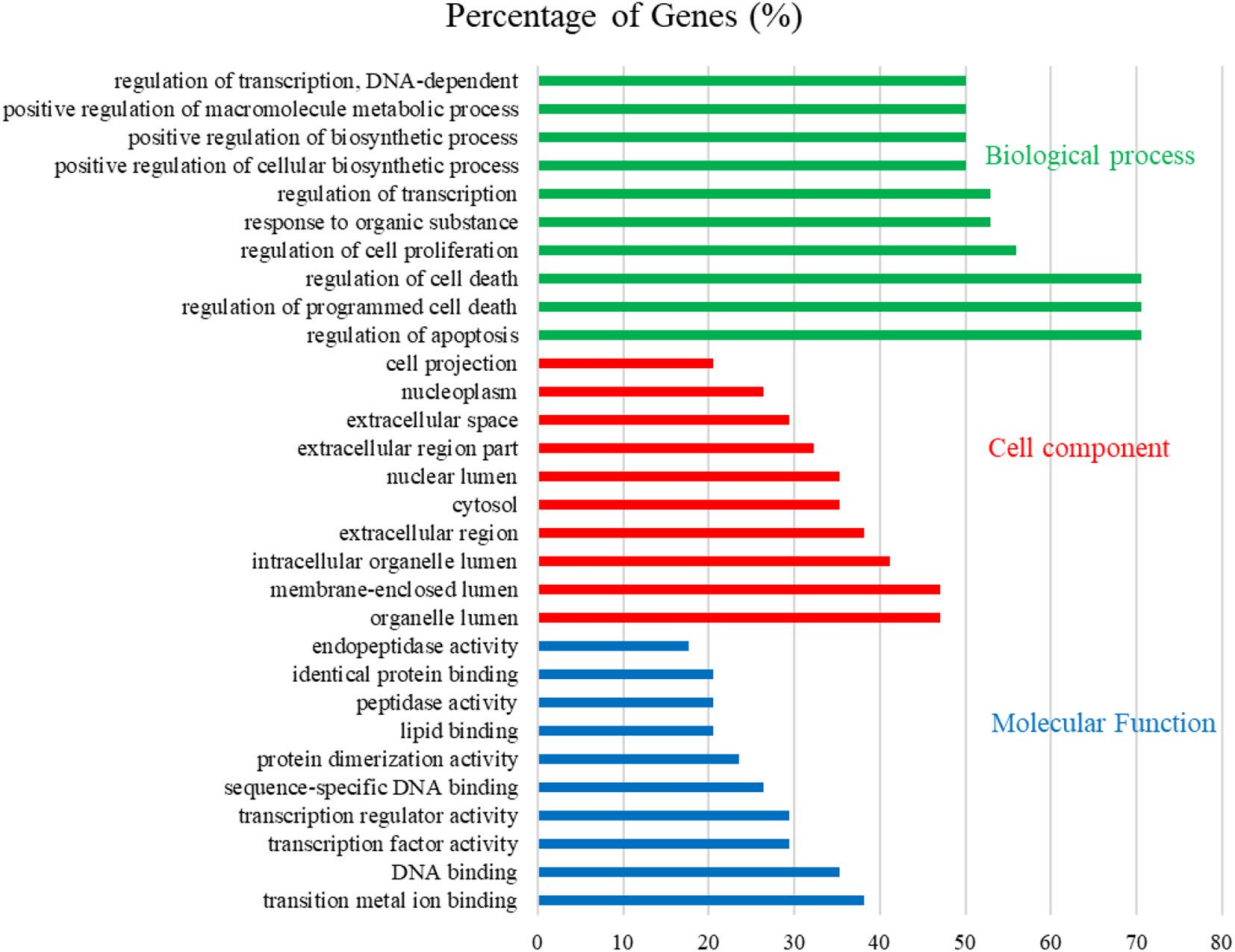
Gene Ontology (GO) analysis of targets of Banxia-Xiakucao Chinese Herb Pair: 34 core candidate targets of BXHP were analyzed. Enrichment results were obtained for biological processes, cell components, and molecular functions; only the top 10 GO terms with *P* < 0.05 are displayed for each category. The terms for each category are presented in ascending order of the percentage of genes.

The 34 core candidate BXHP targets were analyzed using Database for Annotation, Visualization and Integrated Discovery (DAVID), gene ontology (GO) and Kyoto Encyclopedia of Genes and Genomes (KEGG) analyses. The results revealed that 401 biological process (BP), 19 cellular component (CC) and 37 molecular function (MF) terms and 36 pathways were enriched among the targets (*p* < 0.05). The GO and KEGG analysis results are summarized in Fig. 8, Tables S7 and S8; 10 significantly enriched in the BP, CC, and MF terms. The enrichment analysis results indicated that these targets are involved in signal transduction mechanisms and are associated with responses to drugs and other cellular metabolic processes. Based on the DAVID analysis, the BP and MF terms were associated with proteins related to the regulation of cellular processes such as apoptosis, programmed cell death, transition metal ion binding and DNA binding. Additionally, various signalling molecules related to SD, including the toll-like receptor signalling pathway, T cell receptor signalling pathway, MAPK signalling pathway, Jak/STAT signalling pathway, and apoptosis pathway (in order of count), were identified (Table S8).

**Figure 9:**
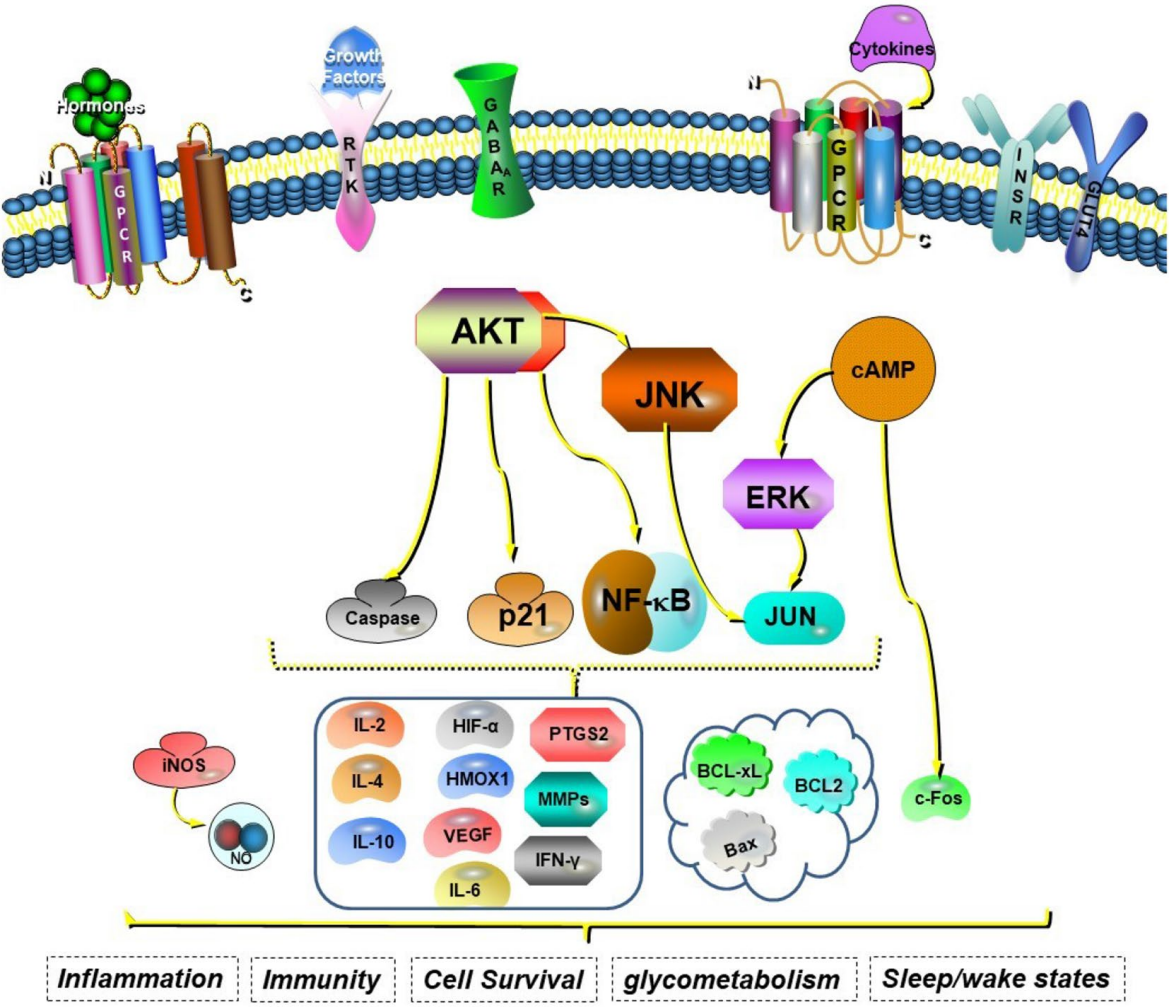
Illustration of the mechanisms by which Banxia-Xiakucao Chinese Herb Pair exerts therapeutic effects in sleep disorder. GPCR: G protein-coupled receptor; RTK: receptor tyrosine kinase; GABAR: gamma-aminobutyric acid receptor; INSR: insulin receptor; AKT(PKB): protein kinase B; JNK: c-Jun N-terminal kinase; ERK: extracellular signal-regulated kinase; cAMP: cyclic adenosine monophosphate; NF-κB: nuclear transcription factor kappa B; iNOS: inducible nitric oxide synthase; NO: nitric oxide; IL: interleukin; HIF-α: hypoxia-inducible factor-α; HMOX1: heme oxygenase (decycling) 1; VEGF: vascular endothelial growth factor; PTGS2: prostaglandin endoperoxide synthase 2; MMP: matrix metalloproteinase; IFN-γ: interferon-γ; BCL: B-cell lymphoma; Bax: BCL2-associated X.

Although the phosphatidylinositol 3-kinase (PI3K)/Akt signalling pathway was not included among the pathways presented in Table S8, these data in combination with a key protein analysis of the 34 core targets revealed that Akt1 was the most significant protein, and that several of the following associated pathway proteins and various differentially expressed genes are involved in PI3K/Akt signalling: JUN, JNK1 MAPK8, ERK2 MAPK1, PTGS2, IL-6, VEGFA, epidermal growth factor receptor (EGFR), matrix metallopeptidase 9 and CASP3. Using a systematic pharmacological approach, we explored key active compounds, targets and pathways associated with the mechanisms underlying the effects of BXHP on SD (Fig. 9).

## Discussion

Recent research investigating the processes associated with SD has gradually become more complex, and several reviews focused on elucidating the mechanisms of SD have been conducted. For example, studies have evaluated the following^30^: (1) how abnormal functioning of the hypothalamic/pituitary/adrenal (HPA) axis leads to increased levels of corticotropin-releasing hormone and cortisol secretion, (2) sympathetic nervous system/vagus nerve disorders, characterized by weakened vagus nerve tone or enhanced sympathetic nerve activity, (3) abnormally low levels of night-time melatonin, (4) excessive inflammatory factor levels, (5) disorders that involve central nervous system (CNS) neurotransmitters, such as 5-HT, norepinephrine (NE) and dopamine (DA) and (6) abnormal function or structures within the limbic-cortex system loop.

According to TCM theory, Phlegm and Heat are pathological products that affect the mind and can result in SD, depression and/or anxiety. BXHP was designed based on the classical therapeutic principle of “clearing Heat, eliminating Phlegm and calming the nerves”. The herbal components in BXHP possess different efficacies and work cooperatively, with *Pinellia ternata* eliminating Phlegm and calming the nerves, and *Prunella vulgaris* clearing Heat and eliminating Phlegm. Although BXHP has been successfully used in TCM for thousands of years, its underlying mechanisms of action remain unclear. However, systems biological and pharmacological network analyses can help identify the multiple targets of TCM ingredients as well as their interactions in terms of molecular networks.

Based on the existing literature combined with the present systematic pharmacological analysis results, the predictions and target pathways associated with the active ingredients in BXHP for SD treatment were evaluated. The main active compounds in BXHP are kaempferol, luteolin, beta-sitosterol, stigmasterol, baicalein and cavidine. Additionally, the compound-target network revealed that BXHP mainly affected targets related to SD, including GABA, c-fos, various cytokines (e.g. IL-6, IL-10, interferon gamma (IFN-γ), hypoxia inducible factor alpha and VEGF), major signalling pathway molecules (Akt1, JUN, ERK2 MAPK1 and insulin receptor), apoptosis-related factors (Bcl-2, Bcl-xL, CASP3 and CASP9) and enzymes (heme oxygenase1 and PTGS2).

Sleep regulation primarily involves neurotransmitters and cytokines. Accordingly, the components of BXHP exert sedative and hypnotic effects via several neurotransmitter proteins and regulate the immune system^31^, especially inflammatory cytokines^32^ important in SD^33^. Additionally, BXHP can improve the symptoms of the secondary hormonal, glucose and lipid metabolic disorders caused by SD.

GABA is a major inhibitory neurotransmitter in the mammalian brain, and its role in improving SD is well known^34^. In particular, GABA_A_ receptors are the main target of natural anxiolytic compounds or sedatives, and therefore these receptors were considered the main targets of BXHP in the present study. Although the main compounds in BXHP (i.e. stigmasterol and beta-sitosterol) regulate sleep structure via the GABA–GABA_A_ receptor system^35^, monoamine neurotransmitters such as DA, NE and 5-HT also play important roles in sleep regulation. Recent studies have shown that BXHP enhances the expression of 5-HT_1A_ and 5-HT_2A_ receptors in the hypothalamus of rats^22^, and that BXHP contains flavonoids such as kaempferol, luteolin and baicalein. Flavonoids can increase the levels of 5-HT, DA and NE in the CNS^36^. In addition, PTGS, also known as cyclooxygenase, is a key enzyme involved in prostaglandin biosynthesis that regulates prostaglandin dioxygenase and peroxidase activities. The primary metabolites of PTGS2 are prostaglandin E2 (PGE2), prostaglandin F2, prostaglandin D2 (PGD2) and prostaglandin I2. PGD2 is the most abundant prostaglandin in the mammalian brain and the most effective endogenous sleep enhancer due to its abilities to induce sleep and inhibit arousal via physiological sleep-regulating mechanisms^37^. In contrast, PGE2 prolongs wake time. Thus, the balance between PGD2 and PGE2 is critical for maintaining a normal sleep–wake cycle. PTGS2 is a target of BXHP and may also be a mechanism for treating SD. At the same time, crucial active compounds such as baicalein and luteolin inhibit the overexpression of inflammatory cytokines, including PGE2 and TNF-α, as well as the expression of cyclooxygenase-2 and inducible nitric oxide synthase (iNOS)^38,39^.

BXHP might also possess extensive anti-inflammatory and immunoregulatory activities via its effects on bioactive compounds and treatment targets. The sleep and immune systems interact with each other, such that activation of the immune system can alter sleep, and insufficient sleep can affect the immune system. Additionally, inflammatory factors are a vital mediator of these actions^40^. For example, during a period of sleep disturbance, nerve fibers from the sympathetic nervous system release NE into lymphoid organs and stimulate the adrenal gland to release stored epinephrine into systemic circulation. Both of these neuromediators stimulate adrenergic receptors on leukocytes and activate nuclear factor kappa beta (NF-κB)-mediated inflammatory pathways^41^. A recent meta-analysis of 72 studies^32^ revealed that more severe sleep disturbances are associated with higher levels of circulating IL-6 and C-reactive protein, and that shorter and longer sleep durations are significantly related to higher IL-6 levels. Furthermore, acute sleep loss may lead to alterations in inflammatory gene expression^42^, as well as upregulation of cellular signalling pathways involving cyclic adenosine monophosphate (cAMP), protein kinase C (PKC), JUN and NF-κB, which are responsible for multiple inflammatory responses^43^.

The present study found that Akt, IL-6, JUN, NF-κB, cAMP, IL-2 and IL-4 are targets of BXHP for the treatment of SD, and that several BXHP compounds exert anti-inflammatory and immunoregulatory effects. For example, the role of kaempferol in reducing inflammation has been validated experimentally, revealing that this compound reduces the levels of several pro-inflammatory cytokines (IL-6, TNF-α and IL-1β) and increases the levels of anti-inflammatory cytokines, including IL-10, by inhibiting PI3K/Akt and NF-κB signalling pathways^44^. Similarly, in murine models, stigmasterol significantly inhibited the lipopolysaccharide-induced febrile response and reduced inflammatory cell proliferation to regulate innate immune responses^45^.

We also performed an enrichment analysis to clarify that Akt1 is the most critical target among the various BXHP targets. Akt1 is an important signalling molecule that modulates a variety of cellular processes, including inflammation, cell growth, survival and metabolism. It was shown that chronic sleep deprivation inhibits Akt phosphorylation and increases glycogen synthase kinase phosphorylation in the hippocampus. Furthermore, those changes occurred in conjunction with increased levels of pro-inflammatory factors (IFN-γ, TNF-α, monocyte chemoattractant protein-1 and IL-1β) as well as decreased levels of anti-inflammatory factors, the transcription factor nuclear factor erythroid 2-related factor 2 and the antioxidant enzyme HMOX1^46^. For example, during the time course of midazolam-induced hypnosis in mice, phosphorylated Akt1 levels markedly increase in the brain cortex, which suggests that Akt1 is involved in sleep-induced neuroplasticity^47^. Additionally, the signalling pathway was centered around Akt1, which was connected to PTGS2, IL-6, JUN, VEGFA, EGFR, ERK2 MAPK1, JNK1 MAPK8, c-Fos and CASP3, as important targets of BXHP for treating SD. The anti-inflammatory, antioxidant, immunoregulatory and glycometabolic effects of BXPH are key for SD treatment as well as a variety of medical problems secondary to SD, such as mental illnesses, cardiovascular disease and diabetes.

The present study focused exclusively on the widely reported effects of BXHP for treating SD, which does not exclude the possibility that this CHM formula exerts other functions. The pathway enrichment analysis revealed that cancer, cardiovascular disease, signal transformation and lipid metabolism were associated with the whole formula, which suggests that multiple functions are involved, and many different pathways are clarified. In general, CHM formulations are considered multifunctional due to their rich chemical diversity.

The present study has several limitations that should be considered. First, the bioactive ingredients and targets that were collected from the available resources may not be comprehensive. Furthermore, the putative active ingredients may not actually exert pharmacological effects. Proteomics technology could be used to further characterize the pharmacological activity and components of CHM. Second, the research are somewhat limited in terms of the quantity and quality of the data. However, the compounds under study have been shown to exert sedative and hypnotic effects in clinical applications for hundreds of years, and animal experimental studies have also confirmed these effects. Nevertheless, the “multi-component, multi-target” properties of BXHP still need to be systematically verified. Third, there may be complex interactions among the Chinese herbs that could influence clinical efficacy. For example, different ratios of *Pinellia ternata* to *Prunella vulgaris* have various effects on the decoction yields of trigonelline, uracil and adenosine^48^. In future research, additional attention should be paid to the interactions among the herbal constituents and the effects of dose. In the future, modern technology will be able to verify the mechanism of action of BXHP in animal models, and the results of this study could serve as a reference.

## Conclusions

In conclusion, the analyses of biologically active compounds, potential targets and compound target–disease networks enabled a systematic pharmacological approach that provided an in-depth understanding of the mechanisms of action underlying the effects of BXHP on SD. Based on these findings, BXHP contains a large number of bioactive compounds with different pharmacological properties. Additionally, the present study showed that the sedative-hypnotic effects of BXHP in patients with SD primarily operate via reductions in inflammation and the mediation of immune and CNS neurotransmitters that regulate multiple targets and pathways.

## Materials and methods

### Chemical database construction for BXHP

All BXHP ingredients were extracted from the following sources: the TCMSP (https://tcmspw.com/tcmsp.php), which is the largest non-commercial database that includes TCM pharmacological data; the National Center for Biotechnology Information PubChem database^49^ (https://pubchem.ncbi.nlm.nih.gov); the DrugBank database (https://www.drugbank.ca/); and large-scale literature mining. Subsequently, a BXHP compound database was developed.

### Prediction of the active compounds in BXHP

Although CHM formulations typically consist of multiple compounds, not all those components are necessarily pharmacologically active. To justify expensive and time-consuming animal and clinical studies, the active ingredients in CHM formulas should be identified. The present study screened for various compounds present in BXHP based on ADME parameters, such as OB^50^ and DL^51^, as used previously^52^. OB refers to the percentage of an orally delivered drug that enters the systemic circulation, which is a key indicator of the properties of bioactive molecules and drugs and has a high effect ratio^53^. DL refers to the structural similarity between herbal ingredients and known drugs. DL is widely used during drug development to evaluate the potential of compounds for inclusion in therapeutic drugs. To identify ingredients that may be absorbed orally and exert curative effects, based on the average value for all compounds in the DrugBank database, the present study selected the following threshold conditions for screening active BXHP compounds: OB ≥ 30% and DL ≥ 0.18^54^.

### Prediction of the putative targets of BXHP

The therapeutic effects of CHM formulas depends on the synergistic effects of multiple components, which also have multiple targets and exert their activity via multiple pathway^55^. Systematically exploring the therapeutic targets of CHM formula is a good way to elucidate the mechanisms of action. In this study, we used the systems drug targeting model (SysDT) model, based on random forest and support vector machine, to identify potential therapeutic targets of candidate compounds^54^. The training set for the SysDT model included 6511 drug molecules and nearly 4000 proteins that interact with drug molecules in the DrugBank database. The likelihood of each ingredient in the TCMSP interacting with its target was determined^56^. The results indicated good ability to predict drug-target interactions; the consistency, sensitivity, and specificity values were 82.83%, 81.33% and 93.62%, respectively. In the present study, the TCMSP database was searched to identify pharmacological targets of individual BXHP compounds^23^; all targets thus identified were imported into the UniProt knowledge base (https://www.uniprot.org/), and all protein names were outputted as official names (official symbol).

### Identification of SD-related targets

Known SD-related targets were identified using the following existing resources: (1) the DrugBank database^57^ (https://www.drugbank.ca/) to identify Food and Drug Administration-approved treatment drugs for SD and human gene/protein target interactions, (2) the OMIM database^58^ using the keyword "insomnia", "sleep disorder", "sleeplessness" (https://omim.org/search/advanced/geneMap), (3) the GeneCards database using "insomnia", "sleep disorder", "sleeplessness" as the keyword (https://www.genecards.org/), and (4) the TTD database^59^ (http://db.idrblab.net/ttd/).

### Identification of candidate BXHP targets responsible for its sedative-hypnotic effects

Based on data obtained using the Bisogenet Cytoscape plugin, a network of interactions between SD-related targets and known potential BXHP pharmacological targets was constructed^60,61^. The frequency data of the target proteins were imported into Cytoscape software (version 3.7.0) and a map was created. We determined the relative importance of each node in the network using two important network topology parameters, namely the degree and betweenness centrality, which can be analyzed using NetworkAnalyzer in Cytoscape software (version 3.7.0). The degree of each node represents the number of other nodes to which it is directly connected, i.e., its centrality; betweenness centrality refers to the number of times a node passes through the shortest path of any other two nodes. This parameter indicates node connectivity. The higher the degree of a node, the more significant it is within the network.

### Constructing the PPI network

Proteins interact in a variety of ways, forming a complex protein–protein interaction (PPI) network that maintain cell activities. To explore the systemic functions of the target proteins, BXHP targets were uploaded to the online STRING database (version 11.0; https://string-db.org/). STRING contains known and predicted PPIs, where the credibility of the interactions is determined by the confidence level (highest confidence, score ≥ 0.9; high confidence or better, score ≥ 0.7; medium confidence or better, score ≥ 0.4; and low confidence or better, score ≥ 0.15). In this study, the score cutoff was set to ≥ 0.4^62^. The proteins isolated from the map are hidden, and the output is the PPI network. Isolated proteins from the map are hidden, and the PPI network is the output. Furthermore, using the CytoNCA Cytoscape plugin, the interactions in the network visualization using Cytoscape software (version 3.7.0) and the topological properties of each node in the interactive network were evaluated based on the betweenness centrality, degree centrality, closeness centrality, eigenvector centrality, network centrality and local average connectivity values^63^. The definitions of, and formulas to calculate, these parameters, which are directly related to the importance of the nodes in the network, have been described previously. We subjected targets with topological feature values exceeding the median to further functional analyses.

### GO and KEGG pathway enrichment analyses

To determine the degree of participation of the 34 core candidate targets of BXHP, these targets were assessed by GO enrichment analyses using DAVID (https://david.ncifcrf.gov/)^64^ according to three different terms (BP, CC and MF). For items with a *p* value < 0.05, hypergeometric tests were used to identify enriched GO terms. Each of the BP, CC, and MF categories had as many as 10 significantly enriched terms according to the GO analysis. Furthermore, a DAVID-based enrichment analysis of the 34 targets was performed using the KEGG pathway database (https://www.kegg.jp/kegg/pathway.html)^65^; only items with a *p* value < 0.05 were selected.

## Supplementary information

Supplementary Information.

## Data Availability

The materials and data used to support the findings of this study are available from public databases.
