# Supplementary material for: Uncovering the pharmacological mechanism of the effects of the Banxia-Xiakucao Chinese Herb Pair on sleep disorder by a systems pharmacology approach
Source: Sci Rep. 2020 Nov 24;10:20454. doi: 10.1038/s41598-020-77431-1 (PMC7686484; doi:10.1038/s41598-020-77431-1)
Supplement: Supplementary file 1 — Supplementary Information. [file 41598_2020_77431_MOESM1_ESM.pdf]

Supplementary tables

Title: Uncovering the Pharmacological Mechanism of the Effects of the Banxia-Xiakucao Chinese Herb Pair on Sleep Disorder by A Systems Pharmacology Approach

Authors: Jing Guo, Meng-Ping Lou, Lin-Lin Hu, Xin Zhang

Supplementary Table S1. All Chemical information of 176 compounds of BXHP

Compound ID	Compound Name	OB(%)	DL	Herb
MOL000105	protocatechuic acid	25.37	0.04	Pinellia ternata
MOL000114	vanillic acid	35.47	0.04	Pinellia ternata
MOL000127	Neral	19.48	0.02	Pinellia ternata
MOL001281	L-alpha-Palmitin	26.66	0.22	Pinellia ternata
MOL000131	EIC	41.9	0.14	Pinellia ternata<br>Prunella vulgaris
MOL001385	9-Oxononanoic acid	19.6	0.03	Pinellia ternata
MOL001396	PENTADECYLIC ACID	20.18	0.08	Pinellia ternata
MOL001452	protocatechualdehyde	38.35	0.03	Pinellia ternata
MOL001492	Ethyl icosanoate	16.67	0.25	Pinellia ternata
MOL000172	Furol	34.35	0.01	Pinellia ternata
MOL001729	Crysophanol	18.64	0.21	Pinellia ternata
MOL001739	zoomaric acid	35.78	0.1	Pinellia ternata
MOL001744	uracil	42.53	0.02	Pinellia ternata
MOL001755	24-Ethylcholest-4-en-3-one	36.08	0.76	Pinellia ternata
MOL001757	GUN	42.45	0.04	Pinellia ternata
MOL001788	adenine	62.81	0.03	Pinellia ternata
MOL001816	Amide HPL	19.79	0.1	Pinellia ternata
MOL001818	Methyl palmitelaidate	34.61	0.12	Pinellia ternata
MOL001831	HX	52.29	0.04	Pinellia ternata
MOL000223	caffeic acid	25.76	0.05	Pinellia ternata<br>Prunella vulgaris
MOL002254	Barolub	16.29	0.22	Pinellia ternata
MOL002495	6-shogaol	31	0.14	Pinellia ternata
MOL002670	Cavidine	35.64	0.81	Pinellia ternata
MOL002714	baicalein	33.52	0.21	Pinellia ternata
MOL002776	Baicalin	40.12	0.75	Pinellia ternata
MOL000346	succinic acid	29.62	0.01	Pinellia ternata
MOL000357	Sitogluside	20.63	0.62	Pinellia ternata<br>Prunella vulgaris
MOL000358	beta-sitosterol	36.91	0.75	Pinellia ternata<br>Prunella vulgaris
MOL000384	DL-Glucuronic acid	3.35	0.04	Pinellia ternata
MOL003870	gynesine	60.07	0.03	Pinellia ternata
MOL000388	gamma-aminobutyric acid	24.09	0.01	Pinellia ternata
MOL000389	FERULIC ACID (CIS)	54.97	0.06	Pinellia ternata
MOL000394	choline	0.47	0.01	Pinellia ternata
MOL003969	L-Serin	98.47	0.01	Pinellia ternata
MOL000397	cis-p-Coumarate	45.98	0.04	Pinellia ternata
MOL003971	Threonin	73.52	0.01	Pinellia ternata

MOL000399	Docosanoate	15.69	0.26	Pinellia ternata<br>Prunella vulgaris
MOL000432	linolenic acid	45.01	0.15	Pinellia ternata
MOL004481	(2S)-2-amino-3-[(2R)-2-amino-3-hydroxy-3-oxopropyl]disulfanylpropanoic acid	73.59	0.05	Pinellia ternata
MOL000449	Stigmasterol	43.83	0.76	Pinellia ternata<br>Prunella vulgaris
MOL000458	campesterol	5.57	0.72	Pinellia ternata
MOL004649	1-[(2R,3R,4S,5S)-3,4-dihydroxy-5-(hydroxymethyl)oxolan-2-yl]pyrimidine-2,4-dione	17.85	0.11	Pinellia ternata
MOL004738	Spantol	2.42	0.04	Pinellia ternata
MOL004739	DAL	85.17	0.01	Pinellia ternata
MOL000475	anethole	32.49	0.03	Pinellia ternata
MOL004796	soya-cerebroside i	3.86	0.37	Pinellia ternata
MOL004797	soya-cerebroside i_qt	21.22	0.51	Pinellia ternata
MOL000050	GLY	48.74	0	Pinellia ternata
MOL005030	gondoic acid	30.7	0.2	Pinellia ternata
MOL005125	ANN	29.69	0.03	Pinellia ternata
MOL000513	3,4,5-trihydroxybenzoic acid	31.69	0.04	Pinellia ternata
MOL000519	coniferin	31.11	0.32	Pinellia ternata
MOL000052	Gulutamine	6.66	0.02	Pinellia ternata
MOL000054	L-	47.64	0.03	Pinellia ternata
MOL005448	Leucinum	72.92	0.01	Pinellia ternata
MOL000055	L-Lysin	29.33	0.02	Pinellia ternata
MOL000056	DTY	57.55	0.05	Pinellia ternata
MOL000579	hydroquinone	29.26	0.02	Pinellia ternata
MOL006240	THM	11.34	0.11	Pinellia ternata
MOL000065	ASI	79.74	0.02	Pinellia ternata
MOL006594	Eciphin	43.35	0.03	Pinellia ternata
MOL006597	OMD	92.44	0.04	Pinellia ternata
MOL000067	L-Valin	53.33	0.01	Pinellia ternata
MOL000675	oleic acid	33.13	0.14	Pinellia ternata<br>Prunella vulgaris
MOL000068	L-Ile	59.05	0.02	Pinellia ternata
MOL006844	Norharman	18.88	0.08	Pinellia ternata
MOL000069	palmitic acid	19.3	0.1	Pinellia ternata<br>Prunella vulgaris
MOL006930	(+) isolariciresinol 9-o-β-d-glucopyranoside	3.83	0.84	Pinellia ternata
MOL006931	isolariciresino	6.96	0.39	Pinellia ternata

MOL006932	l-Pseudoephedrine	45.01	0.03	Pinellia ternata
MOL006933	1,2,3,4,6-penta-o-galloyl-β-d-glucose	3.01	0.13	Pinellia ternata
MOL006934	WLN: Q5Q	24.8	0.01	Pinellia ternata
MOL006935	Octylene	39.25	0.01	Pinellia ternata
MOL006936	10,13-eicosadienoic	39.99	0.2	Pinellia ternata
MOL006937	12,13-epoxy-9-hydroxynonadeca-7,10-dienoic acid	42.15	0.24	Pinellia ternata
MOL006938	DUR	23.69	0.09	Pinellia ternata
MOL006939	Methylpyrazine	27.1	0.01	Pinellia ternata
MOL006940	D-2-Aminobutyrate	68.78	0.01	Pinellia ternata
MOL006941	3-methyleicosa	10.18	0.15	Pinellia ternata
MOL006942	N-(5-methylisoxazol-3-yl)acetamide	20.82	0.02	Pinellia ternata
MOL006943	5,8-epidioxyergosta-6,22-dien-3-ol	22.71	0.82	Pinellia ternata
MOL006944	8-Octadecenoic acid	33.13	0.14	Pinellia ternata
MOL006945	9-Heptadecanol	14.24	0.09	Pinellia ternata
MOL006946	adenosine	19.85	0.16	Pinellia ternata
MOL006947	heptadecanoic acid 2,3-dihydroxy-propyl ester	25.9	0.26	Pinellia ternata
MOL006948	inosine	11.17	0.18	Pinellia ternata
MOL006949	6-Deoxy-gulose	44.03	0.03	Pinellia ternata
MOL006950	(2R)-2-acetamidoglutaric acid	15.5	0.04	Pinellia ternata
MOL006951	pedatisectine a	64.09	0.16	Pinellia ternata
MOL006952	pedatisectine f	53.81	0.06	Pinellia ternata
MOL006953	Thy	74.2	0.02	Pinellia ternata
MOL006954	BVE	42.32	0.01	Pinellia ternata
MOL000875	Cedrol	16.23	0.12	Pinellia ternata
MOL006956	cyclo-(leu-tyr)	111.16	0.15	Pinellia ternata
MOL006957	(3S,6S)-3-(benzyl)-6-(4-hydroxybenzyl)piperazin-e2,5-quinone	46.89	0.27	Pinellia ternata
MOL006958	cyclo-(val-tyr)	122.79	0.14	Pinellia ternata
MOL003578	Cycloartenol	38.69	0.78	Pinellia ternata
MOL006960	(5R)-5-hydroxy-1-(4-hydroxy-3-methoxyphenyl)tetradecan-3-one	19.14	0.28	Pinellia ternata
MOL006961	GR	20.9	0.21	Pinellia ternata
MOL006962	2Z-hexadecenoic acid	34.02	0.1	Pinellia ternata
MOL006963	Ethyl linolelaidate	7.33	0.19	Pinellia ternata
MOL006964	valeraldoxime	82.58	0.01	Pinellia ternata
MOL006965	soya-cerebroside ii	3.86	0.35	Pinellia ternata

MOL006966	soya-cerebroside ii_qt	21.22	0.49	Pinellia ternata
MOL006967	beta-D-Ribofuranoside, xanthine-9	44.72	0.21	Pinellia ternata
MOL000697	MRY	59.62	0.01	Pinellia ternata
MOL000708	WLN: VHR	32.63	0.01	Pinellia ternata
MOL000071	Istidina	53.18	0.03	Pinellia ternata
MOL000748	HMF	45.07	0.02	Pinellia ternata
MOL000774	(-)-Citronellal	35.71	0.02	Pinellia ternata
MOL000858	Glycerol palmitate	26.66	0.22	Pinellia ternata
MOL000860	stearic acid	17.83	0.14	Pinellia ternata Prunella vulgaris
MOL000089	catechol	29.86	0.02	Pinellia ternata
MOL000908	beta-elemene	25.63	0.06	Pinellia ternata
MOL000924	Mnk	17.66	0.03	Pinellia ternata
MOL000971	Ethylpalmitate	18.99	0.14	Pinellia ternata Prunella vulgaris
MOL000122	1,8-cineole	39.73	0.05	Prunella vulgaris
MOL000126	(-)-nopinene	44.84	0.05	Prunella vulgaris
MOL000130	CAM	67.17	0.05	Prunella vulgaris
MOL001393	myristic acid	21.18	0.07	Prunella vulgaris
MOL001654	Oleanolic acid-28-O-beta-D-glucopyranoside	11.48	0.41	Prunella vulgaris
MOL000197	Myrcene	24.96	0.02	Prunella vulgaris
MOL000002	cyanidol	1.36	0.24	Prunella vulgaris
MOL002004	endo-fenchol	76.43	0.05	Prunella vulgaris
MOL002558	Skimmetin	27.37	0.05	Prunella vulgaris
MOL000263	oleanolic acid	29.02	0.76	Prunella vulgaris
MOL002841	l-alpha-Fenchone	72.64	0.05	Prunella vulgaris
MOL002902	Ethyl caffeate	103.85	0.07	Prunella vulgaris
MOL000305	lauric acid	23.59	0.04	Prunella vulgaris
MOL000361	Amyrin	17.6	0.76	Prunella vulgaris
MOL003837	esculetin	22.97	0.07	Prunella vulgaris
MOL000040	Scopoletol	27.77	0.08	Prunella vulgaris
MOL000415	rutin	3.2	0.68	Prunella vulgaris
MOL000422	kaempferol	41.88	0.24	Prunella vulgaris
MOL004355	Spinasterol	42.98	0.76	Prunella vulgaris
MOL004368	Hyperin	6.94	0.77	Prunella vulgaris
MOL004652	(2R,3R,4S,5S,6R)-2-[[(3S,5S,9R,10S,13R,14R,17R)-17-[(E,2R,5S)-5-ethyl-6-methylhept-3-en-2-yl]-10,13-dimethyl-2,3,4,5,6,9,11,12,14,15,16,17-dodecahydro-1H-	21.2	0.63	Prunella vulgaris

	cyclopenta[a]phenanthren-3-yl]oxy]-6-(hydroxymethyl)oxan-e3,4,5-triol			
MOL004798	delphinidin	40.63	0.28	Prunella vulgaris
MOL000499	malvidin	2.48	0.34	Prunella vulgaris
MOL000511	ursolic acid	16.77	0.75	Prunella vulgaris
MOL000515	Melissic acid	13.22	0.49	Prunella vulgaris
MOL005151	[(3S)-3,7-dimethylocta-1,6-dien-3-yl] acetate	36.84	0.04	Prunella vulgaris
MOL005217	Δ7-stigmasterol	13.01	0.76	Prunella vulgaris
MOL000561	Astragalin	14.03	0.74	Prunella vulgaris
MOL000006	luteolin	36.16	0.25	Prunella vulgaris
MOL000659	Montanic acid	13.69	0.46	Prunella vulgaris
MOL000663	lignoceric acid	14.9	0.33	Prunella vulgaris
MOL006763	Malvidin-3,5-diglucoside	13.89	0.63	Prunella vulgaris
MOL006764	Peonin	13.71	0.69	Prunella vulgaris
MOL006765	peonidin	26.92	0.27	Prunella vulgaris
MOL006766	Vulgarsaponin B	18.75	0.23	Prunella vulgaris
MOL006767	Vulgaxanthin-I	56.14	0.26	Prunella vulgaris
MOL006768	arjunglucoside I	8.11	0.35	Prunella vulgaris
MOL006769	isoquercetrin	3.86	0.78	Prunella vulgaris
MOL006770	niga-chigoside F1	8.1	0.35	Prunella vulgaris
MOL006771	poriferasterol monoglucoside	21.32	0.63	Prunella vulgaris
MOL006772	poriferasterol monoglucoside_qt	43.83	0.76	Prunella vulgaris
MOL006773	sericoside	7.76	0.35	Prunella vulgaris
MOL006774	stigmast-7-enol	37.42	0.75	Prunella vulgaris
MOL000698	(R)-(-)-alpha-Phellandrene	27.51	0.02	Prunella vulgaris
MOL000737	morin	46.23	0.27	Prunella vulgaris
MOL000771	p-coumaric acid	43.29	0.04	Prunella vulgaris
MOL000498	isoorientin	23.3	0.76	Prunella vulgaris
MOL000870	HEXATRIACONTANE	7.95	0.41	Prunella vulgaris
MOL000009	luteolin-7-o-glucoside	7.29	0.78	Prunella vulgaris
MOL000098	quercetin	46.43	0.28	Prunella vulgaris

OB: Oral Bioavailability; DL: Drug-Likeness

Supplementary Table S2. Chemical information of 22 candidate compounds of BXHP

Compound ID	Compound name	OB(%)	DL	Herb
MOL001755	24-Ethylcholest-4-en-3-one	36.08	0.76	Pinellia ternate
MOL002670	Cavidine	35.64	0.81	Pinellia ternate
MOL002714	baicalein	33.52	0.21	Pinellia ternate
MOL002776	Baicalin	40.12	0.75	Pinellia ternate
MOL000358	beta-sitosterol	36.91	0.75	Pinellia ternate<br>Prunella vulgaris
MOL000449	Stigmasterol	43.83	0.76	Pinellia ternate<br>Prunella vulgaris
MOL005030	gondoic acid	30.7	0.2	Pinellia ternate
MOL000519	coniferin	31.11	0.32	Pinellia ternate
MOL006936	10,13-eicosadienoic	39.99	0.2	Pinellia ternate
MOL006937	12,13-epoxy-9-hydroxynonadeca-7,10-dienoic acid	42.15	0.24	Pinellia ternate
MOL006957	(3S,6S)-3-(benzyl)-6-(4-hydroxybenzyl)piperazine-2,5-quinone	46.89	0.27	Pinellia ternate
MOL003578	Cycloartenol	38.69	0.78	Pinellia ternate
MOL006967	beta-D-Ribofuranoside, xanthine-9	44.72	0.21	Pinellia ternate
MOL000422	kaempferol	41.88	0.24	Prunella vulgaris
MOL004355	Spinasterol	42.98	0.76	Prunella vulgaris
MOL004798	delphinidin	40.63	0.28	Prunella vulgaris
MOL000006	luteolin	36.16	0.25	Prunella vulgaris
MOL006767	Vulgaxanthin-I	56.14	0.26	Prunella vulgaris
MOL006772	poriferasterol monoglucoside_qt	43.83	0.76	Prunella vulgaris
MOL006774	stigmast-7-enol	37.42	0.75	Prunella vulgaris
MOL000737	morin	46.23	0.27	Prunella vulgaris
MOL000098	quercetin	46.43	0.28	Prunella vulgaris

OB: Oral Bioavailability; DL: Drug-Likeness

Supplementary Table S3. All targets of 19 candidate compounds

Compound ID	Compound Name	Target	Symbol
MOL001755	24-Ethylcholest-4-en-3-one	Progesterone receptor	PGR
MOL001755	24-Ethylcholest-4-en-3-one	Mineralocorticoid receptor	NR3C2
MOL002670	Cavidine	Prostaglandin G/H synthase 1	PTGS1
MOL002670	Cavidine	Muscarinic acetylcholine receptor M3	CHRM3
MOL002670	Cavidine	Potassium voltage-gated channel subfamily H member 2	KCNH2
MOL002670	Cavidine	Muscarinic acetylcholine receptor M1	CHRM1
MOL002670	Cavidine	Beta-1 adrenergic receptor	ADRB1
MOL002670	Cavidine	Sodium channel protein type 5 subunit alpha	SCN5A
MOL002670	Cavidine	Muscarinic acetylcholine receptor M5	CHRM5
MOL002670	Cavidine	Prostaglandin G/H synthase 2	PTGS2
MOL002670	Cavidine	5-hydroxytryptamine receptor 3A	HTR3A
MOL002670	Cavidine	Alpha-2C adrenergic receptor	ADRA2C
MOL002670	Cavidine	Muscarinic acetylcholine receptor M4	CHRM4
MOL002670	Cavidine	Retinoic acid receptor RXR-alpha	RXRA
MOL002670	Cavidine	Delta-type opioid receptor	OPRD1
MOL002670	Cavidine	Alpha-1B adrenergic receptor	ADRA1B
MOL002670	Cavidine	Beta-2 adrenergic receptor	ADRB2
MOL002670	Cavidine	Alpha-1D adrenergic receptor	ADRA1D
MOL002670	Cavidine	Mu-type opioid receptor	OPRM1
MOL002670	Cavidine	Retinoic acid receptor RXR-beta	RXRB
MOL002670	Cavidine	Sodium-dependent serotonin transporter	SLC6A4
MOL002670	Cavidine	Coagulation factor VII	F7
MOL002670	Cavidine	cAMP and cAMP-inhibited cGMP 3',5'-cyclic phosphodiesterase 10A	PDE10A
MOL002714	baicalein	Prostaglandin G/H synthase 1	PTGS1
MOL002714	baicalein	Androgen receptor	AR
MOL002714	baicalein	Prostaglandin G/H synthase 2	PTGS2
MOL002714	baicalein	Trypsin-1	PRSS1
MOL002714	baicalein	Nuclear receptor coactivator 2	NCOA2
MOL002714	baicalein	Nuclear receptor coactivator 1	NCOA1
MOL002714	baicalein	Transcription factor p65	RELA

MOL002714	baicalein	RAC-alpha serine/threonine-protein kinase	AKT1
MOL002714	baicalein	Vascular endothelial growth factor A	VEGFA
MOL002714	baicalein	Apoptosis regulator Bcl-2	BCL2
MOL002714	baicalein	Proto-oncogene c-Fos	FOS
MOL002714	baicalein	Apoptosis regulator BAX	BAX
MOL002714	baicalein	Matrix metalloproteinase-9	MMP9
MOL002714	baicalein	Caspase-3	CASP3
MOL002714	baicalein	Cellular tumor antigen p53	TP63
MOL002714	baicalein	Hypoxia-inducible factor 1-alpha	HIF1A
MOL002714	baicalein	Fos-related antigen 1	FOSL1
MOL002714	baicalein	Fos-related antigen 2	FOSL2
MOL002714	baicalein	G2/mitotic-specific cyclin-B1	CCNB1
MOL002714	baicalein	Myeloperoxidase	MPO
MOL002714	baicalein	Aryl hydrocarbon receptor	AHR
MOL002714	baicalein	Insulin-like growth factor II	IGF2
MOL002714	baicalein	Cytochrome c	CYCS
MOL002714	baicalein	Arachidonate 12-lipoxygenase, 12S-type	ALOX12
MOL002714	baicalein	Nuclear factor of activated T-cells, cytoplasmic 1	NFATC1
MOL002714	baicalein	Tudor domain-containing protein 7	TDRD7
MOL002714	baicalein	Egl nine homolog 1	EGLN1
MOL002714	baicalein	NADPH oxidase 5	NOX5
MOL002714	baicalein	Apolipoprotein D	APOD
MOL000358	beta-sitosterol	Progesterone receptor	PGR
MOL000358	beta-sitosterol	Nuclear receptor coactivator 2	NCOA2
MOL000358	beta-sitosterol	Prostaglandin G/H synthase 1	PTGS1
MOL000358	beta-sitosterol	Prostaglandin G/H synthase 2	PTGS2
MOL000358	beta-sitosterol	Potassium voltage-gated channel subfamily H member 2	KCNH2
MOL000358	beta-sitosterol	Muscarinic acetylcholine receptor M3	CHRM3
MOL000358	beta-sitosterol	Muscarinic acetylcholine receptor M1	CHRM1
MOL000358	beta-sitosterol	Sodium channel protein type 5 subunit alpha	SCN5A
MOL000358	beta-sitosterol	Muscarinic acetylcholine receptor M4	CHRM4
MOL000358	beta-sitosterol	Alpha-1A adrenergic receptor	ADRA1A

MOL000358	beta-sitosterol	Muscarinic acetylcholine receptor M2	CHRM2
MOL000358	beta-sitosterol	Alpha-1B adrenergic receptor	ADRA1B
MOL000358	beta-sitosterol	Beta-2 adrenergic receptor	ADRB2
MOL000358	beta-sitosterol	Neuronal acetylcholine receptor subunit alpha-2	CHRNA2
MOL000358	beta-sitosterol	Sodium-dependent serotonin transporter	SLC6A4
MOL000358	beta-sitosterol	Mu-type opioid receptor	OPRM1
MOL000358	beta-sitosterol	Gamma-aminobutyric acid receptor subunit alpha-1	GABRA1
MOL000358	beta-sitosterol	Apoptosis regulator Bcl-2	BCL2
MOL000358	beta-sitosterol	Apoptosis regulator BAX	BAX
MOL000358	beta-sitosterol	Caspase-9	CASP9
MOL000358	beta-sitosterol	Transcription factor AP-1	JUN
MOL000358	beta-sitosterol	Caspase-3	CASP3
MOL000358	beta-sitosterol	Caspase-8	CASP8
MOL000358	beta-sitosterol	Protein kinase C alpha type	PRKCA
MOL000358	beta-sitosterol	Serum paraoxonase/arylesterase 1	PON1
MOL000358	beta-sitosterol	Microtubule-associated protein 2	MAP2
MOL000449	Stigmasterol	Progesterone receptor	PGR
MOL000449	Stigmasterol	Mineralocorticoid receptor	NR3C2
MOL000449	Stigmasterol	Nuclear receptor coactivator 2	NCOA2
MOL000449	Stigmasterol	Alcohol dehydrogenase 1C	ADH1C
MOL000449	Stigmasterol	Retinoic acid receptor RXR-alpha	RXRA
MOL000449	Stigmasterol	Nuclear receptor coactivator 1	NCOA1
MOL000449	Stigmasterol	Prostaglandin G/H synthase 1	PTGS1
MOL000449	Stigmasterol	Prostaglandin G/H synthase 2	PTGS2
MOL000449	Stigmasterol	Alpha-2A adrenergic receptor	ADRA2A
MOL000449	Stigmasterol	Sodium-dependent noradrenaline transporter	SLC6A2
MOL000449	Stigmasterol	Sodium-dependent dopamine transporter	SLC6A3
MOL000449	Stigmasterol	Beta-2 adrenergic receptor	ADRB2
MOL000449	Stigmasterol	Aldose reductase	AKR1B1
MOL000449	Stigmasterol	Urokinase-type plasminogen activator	PLAU
MOL000449	Stigmasterol	Leukotriene A-4 hydrolase	LTA4H
MOL000449	Stigmasterol	Amine oxidase [flavin-containing] B	MAOB

MOL000449	Stigmasterol	Amine oxidase [flavin-containing] A	MAOA
MOL000449	Stigmasterol	Chymotrypsinogen B	CTRB1
MOL000449	Stigmasterol	Muscarinic acetylcholine receptor M3	CHRM3
MOL000449	Stigmasterol	Muscarinic acetylcholine receptor M1	CHRM1
MOL000449	Stigmasterol	Beta-1 adrenergic receptor	ADRB1
MOL000449	Stigmasterol	Sodium channel protein type 5 subunit alpha	SCN5A
MOL000449	Stigmasterol	Alpha-1A adrenergic receptor	ADRA1A
MOL000449	Stigmasterol	Muscarinic acetylcholine receptor M2	CHRM2
MOL000449	Stigmasterol	Alpha-1B adrenergic receptor	ADRA1B
MOL000449	Stigmasterol	Gamma-aminobutyric acid receptor subunit alpha-1	GABRA1
MOL005030	gondoic acid	Prostaglandin G/H synthase 1	PTGS1
MOL005030	gondoic acid	Nuclear receptor coactivator 2	NCOA2
MOL000519	coniferin	Muscarinic acetylcholine receptor M3	CHRM3
MOL000519	coniferin	Muscarinic acetylcholine receptor M1	CHRM1
MOL000519	coniferin	Estrogen receptor	ESR1
MOL000519	coniferin	Androgen receptor	AR
MOL000519	coniferin	Sodium channel protein type 5 subunit alpha	SCN5A
MOL000519	coniferin	Peroxisome proliferator activated receptor gamma	PPARG
MOL000519	coniferin	Prostaglandin G/H synthase 2	PTGS2
MOL000519	coniferin	Alpha-1B adrenergic receptor	ADRA1B
MOL000519	coniferin	Beta-2 adrenergic receptor	ADRB2
MOL000519	coniferin	Alpha-1D adrenergic receptor	ADRA1D
MOL000519	coniferin	Mu-type opioid receptor	OPRM1
MOL000519	coniferin	Cyclin-A2	CCNA2
MOL000519	coniferin	Nuclear receptor coactivator 2	NCOA2
MOL000519	coniferin	Nuclear receptor coactivator 1	NCOA1
MOL006936	10,13-eicosadienoic	Prostaglandin G/H synthase 1	PTGS1
MOL006936	10,13-eicosadienoic	Nuclear receptor coactivator 2	NCOA2
MOL006957	(3S,6S)-3-(benzyl)-6-(4-hydroxybenzyl)piperazine-2,5-quinone	Androgen receptor	AR
MOL006957	(3S,6S)-3-(benzyl)-6-(4-	Prostaglandin G/H synthase 2	PTGS2

	hydroxybenzyl)piperazine-2,5-quinone		
MOL006957	(3S,6S)-3-(benzyl)-6-(4-hydroxybenzyl)piperazine-2,5-quinone	Beta-2 adrenergic receptor	ADRB2
MOL003578	Cycloartenol	Mineralocorticoid receptor	NR3C2
MOL006967	beta-D-Ribofuranoside, xanthine-9	Purine nucleoside phosphorylase	MTAP
MOL006967	beta-D-Ribofuranoside, xanthine-9	Prostaglandin G/H synthase 2	PTGS2
MOL000422	kaempferol	Nitric oxide synthase, inducible	NOS2
MOL000422	kaempferol	Prostaglandin G/H synthase 1	PTGS1
MOL000422	kaempferol	Androgen receptor	AR
MOL000422	kaempferol	Peroxisome proliferator activated receptor gamma	PPARG
MOL000422	kaempferol	Prostaglandin G/H synthase 2	PTGS2
MOL000422	kaempferol	Nuclear receptor coactivator 2	NCOA2
MOL000422	kaempferol	Trypsin-1	PRSS1
MOL000422	kaempferol	Progesterone receptor	PGR
MOL000422	kaempferol	Muscarinic acetylcholine receptor M1	CHRM1
MOL000422	kaempferol	Acetylcholinesterase	ACHE
MOL000422	kaempferol	Sodium-dependent noradrenaline transporter	SLC6A2
MOL000422	kaempferol	Muscarinic acetylcholine receptor M2	CHRM2
MOL000422	kaempferol	Alpha-1B adrenergic receptor	ADRA1B
MOL000422	kaempferol	Gamma-aminobutyric acid receptor subunit alpha-1	GABRA1
MOL000422	kaempferol	Coagulation factor VII	F7
MOL000422	kaempferol	Transcription factor p65	RELA
MOL000422	kaempferol	Inhibitor of nuclear factor kappa-B kinase subunit beta	IKBKB
MOL000422	kaempferol	RAC-alpha serine/threonine-protein kinase	AKT1
MOL000422	kaempferol	Apoptosis regulator Bcl-2	BCL2
MOL000422	kaempferol	Apoptosis regulator BAX	BAX
MOL000422	kaempferol	Tumor necrosis factor	TNFSF15
MOL000422	kaempferol	Transcription factor AP-1	JUN
MOL000422	kaempferol	Activator of 90 kDa heat shock protein ATPase homolog 1	AHSA1
MOL000422	kaempferol	Caspase-3	CASP3
MOL000422	kaempferol	Mitogen-activated protein kinase 8	MAPK8

MOL000422	kaempferol	Interstitial collagenase	MMP1
MOL000422	kaempferol	Signal transducer and activator of transcription 1-alpha/beta	STAT1
MOL000422	kaempferol	Peroxisome proliferator-activated receptor gamma	PPARG
MOL000422	kaempferol	Heme oxygenase 1	HMOX1
MOL000422	kaempferol	Cytochrome P450 3A4	CYP3A4
MOL000422	kaempferol	Cytochrome P450 1A2	CYP1A2
MOL000422	kaempferol	Cytochrome P450 1A1	CYP1A1
MOL000422	kaempferol	Intercellular adhesion molecule 1	ICAM1
MOL000422	kaempferol	e-selectin	SELE
MOL000422	kaempferol	Vascular cell adhesion protein 1	VCAM1
MOL000422	kaempferol	Nuclear receptor subfamily 1 group I member 2	NR1I2
MOL000422	kaempferol	Cytochrome P450 1B1	CYP1B1
MOL000422	kaempferol	Arachidonate 5-lipoxygenase	ALOX5
MOL000422	kaempferol	Hyaluronan synthase 2	HAS2
MOL000422	kaempferol	Glutathione S-transferase P	GSTP1
MOL000422	kaempferol	Aryl hydrocarbon receptor	AHR
MOL000422	kaempferol	26S proteasome non-ATPase regulatory subunit 3	PSMD3
MOL000422	kaempferol	Solute carrier family 2, facilitated glucose transporter member 4	SLC2A4
MOL000422	kaempferol	Nuclear receptor subfamily 1 group I member 3	NR1I3
MOL000422	kaempferol	Insulin receptor	INSR
MOL000422	kaempferol	Type I iodothyronine deiodinase	DIO1
MOL000422	kaempferol	Serine/threonine-protein phosphatase 2B catalytic subunit alpha isoform	PPP3CA
MOL000422	kaempferol	Glutathione S-transferase Mu 1	GSTM1
MOL000422	kaempferol	Glutathione S-transferase Mu 2	GSTM2
MOL000422	kaempferol	Aldo-keto reductase family 1 member C3	AKR1C3
MOL000422	kaempferol	Antileukoproteinase	SLPI
MOL004355	Spinasterol	Progesterone receptor	PGR
MOL004355	Spinasterol	Mineralocorticoid receptor	NR3C2
MOL004355	Spinasterol	Nuclear receptor coactivator 2	NCOA2
MOL004798	delphinidin	Nitric oxide synthase, inducible	NOS2
MOL004798	delphinidin	Prostaglandin G/H synthase 1	PTGS1
MOL004798	delphinidin	Androgen receptor	AR
MOL004798	delphinidin	Prostaglandin G/H synthase 2	PTGS2

MOL004798	delphinidin	Nuclear receptor coactivator 2	NCOA2
MOL000006	luteolin	Prostaglandin G/H synthase 1	PTGS1
MOL000006	luteolin	Androgen receptor	AR
MOL000006	luteolin	Prostaglandin G/H synthase 2	PTGS2
MOL000006	luteolin	Trypsin-1	PRSS1
MOL000006	luteolin	Nuclear receptor coactivator 2	NCOA2
MOL000006	luteolin	Transcription factor p65	RELA
MOL000006	luteolin	Epidermal growth factor receptor	EGFR
MOL000006	luteolin	RAC-alpha serine/threonine-protein kinase	AKT1
MOL000006	luteolin	Vascular endothelial growth factor A	VEGFA
MOL000006	luteolin	G1/S-specific cyclin-D1	CCND1
MOL000006	luteolin	Bcl-2-like protein 1	BCL2L1
MOL000006	luteolin	Cyclin-dependent kinase inhibitor 1	CDKN1 A
MOL000006	luteolin	Caspase-9	CASP9
MOL000006	luteolin	72 kDa type IV collagenase	MMP2
MOL000006	luteolin	Matrix metalloproteinase-9	MMP9
MOL000006	luteolin	Mitogen-activated protein kinase 1	MAPK1
MOL000006	luteolin	Interleukin-10	IL10
MOL000006	luteolin	Retinoblastoma-associated protein	RB1
MOL000006	luteolin	Tumor necrosis factor	TNFSF15
MOL000006	luteolin	Transcription factor AP-1	JUN
MOL000006	luteolin	Interleukin-6	IL6
MOL000006	luteolin	Caspase-3	CASP3
MOL000006	luteolin	Cellular tumor antigen p53	TP63
MOL000006	luteolin	NF-kappa-B inhibitor alpha	NFKBIA
MOL000006	luteolin	DNA topoisomerase 1	TOP1
MOL000006	luteolin	E3 ubiquitin-protein ligase Mdm2	MDM2
MOL000006	luteolin	Amyloid beta A4 protein	APP
MOL000006	luteolin	Interstitial collagenase	MMP1
MOL000006	luteolin	Proliferating cell nuclear antigen	PCNA
MOL000006	luteolin	Receptor tyrosine-protein kinase erbB-2	ERBB2
MOL000006	luteolin	Peroxisome proliferator-activated receptor gamma	PPARG
MOL000006	luteolin	Heme oxygenase 1	HMOX1
MOL000006	luteolin	Caspase-7	CASP7
MOL000006	luteolin	Intercellular adhesion molecule	ICAM1

		1	
MOL000006	luteolin	Induced myeloid leukemia cell differentiation protein Mcl-1	MCL1
MOL000006	luteolin	Baculoviral IAP repeat-containing protein 5	BIRC5
MOL000006	luteolin	Interleukin-2	IL2
MOL000006	luteolin	G2/mitotic-specific cyclin-B1	CCNB1
MOL000006	luteolin	Tyrosinase	TYR
MOL000006	luteolin	Interferon gamma	IFNG
MOL000006	luteolin	Interleukin-4	IL4
MOL000006	luteolin	DNA topoisomerase 2-alpha	TOP2A
MOL000006	luteolin	Glutathione S-transferase P	GSTP1
MOL000006	luteolin	Solute carrier family 2, facilitated glucose transporter member 4	SLC2A4
MOL000006	luteolin	Insulin receptor	INSR
MOL000006	luteolin	CD40 ligand	CD40LG
MOL000006	luteolin	Prostaglandin E synthase	PTGES
MOL000006	luteolin	Kinetochore protein Nuf2	NUF2
MOL000006	luteolin	Adenylate cyclase type 2	ADCY2
MOL000006	luteolin	Hepatocyte growth factor receptor	MET
MOL006767	Vulgaxanthin-I	Nitric oxide synthase, inducible	NOS2
MOL006767	Vulgaxanthin-I	Prostaglandin G/H synthase 2	PTGS2
MOL006772	poriferasterol monoglucoside_qt	Progesterone receptor	PGR
MOL006772	poriferasterol monoglucoside_qt	Nuclear receptor coactivator 2	NCOA2
MOL006774	stigmast-7-enol	Progesterone receptor	PGR
MOL006774	stigmast-7-enol	Nuclear receptor coactivator 2	NCOA2
MOL000737	morin	Prostaglandin G/H synthase 1	PTGS1
MOL000737	morin	Androgen receptor	AR
MOL000737	morin	Peroxisome proliferator activated receptor gamma	PPARG
MOL000737	morin	Prostaglandin G/H synthase 2	PTGS2
MOL000737	morin	DNA topoisomerase 1	TOP1
MOL000737	morin	Endothelin-1	EDN3
MOL000737	morin	Multidrug resistance protein 1	ABCB1
MOL000737	morin	Arachidonate 5-lipoxygenase	ALOX5
MOL000737	morin	Platelet glycoprotein 4	CD36
MOL000737	morin	Type I iodothyronine deiodinase	DIO1
MOL000737	morin	Glutathione reductase, mitochondrial	GSR
MOL000737	morin	Basic leucine zipper	BATF3

	transcriptional factor ATF-like 3

Supplementary Table S4. SD-Related Targets

Gene Symbol	Source	Gene Symbol	Source	Gene Symbol	Source
SLC6A4	GC	PDHX	GC	MRPL23	GC
HCRT	GC DrugBank	PLEKHB2	GC	HAP1	GC
BDNF	GC	TSEN2	GC	FRG1	GC
IL6	GC	FTSJ1	GC	PLOD2	GC
HTR2A	GC OMIM TTD	PLEKHM1	GC	CPA1	GC
SLC6A3	GC	STH	GC	NELL1	GC
TNF	GC	ATF4	GC	FAM155A	GC
DRD2	GC	ACAT1	GC	TOR2A	GC
APOE	GC	PLAG1	GC	ZNF589	GC
COMT	GC	TPPP	GC	BSPRY	GC
LEP	GC	ZIC2	GC	FAM170B	GC
IGF1	GC	CEBPD	GC	CD200	GC
PER3	GC	TSEN15	GC	B4GALNT2	GC
MAPT	GC	COQ8B	GC	TADA2B	GC
GRIN2A	GC	FOXO1	GC	SLC4A2	GC
INS	GC	AMPH	GC	SPATS2L	GC
SNCA	GC	GNAI2	GC	RAB11FIP2	GC
PER2	GC	RHD	GC	INSM1	GC
MAOA	GC	BLK	GC	DEFB119	GC
MECP2	GC	PAPPA	GC	BPIFB6	GC
ACE	GC	NAT8L	GC	SPTSSA	GC
RYR1	GC	CD81	GC	VPS37D	GC
GHRL	GC	SHROOM4	GC	ELP6	GC
POMC	GC	MGR2	GC	TRIM73	GC
ALB	GC	RSPO1	GC	STAG3L1	GC
TH	GC	YWHAH	GC	STAG3L4	GC
DRD4	GC OMIM	TKT	GC	STAG3L2	GC
DRD3	GC	TGM3	GC	STAG3L3	GC
EDN1	GC	LOC108663993	GC	SPDYE7P	GC
IL1B	GC	PPAN	GC	LINC01163	GC
CACNA1C	GC	BCORL1	GC	EIF4HP1	GC
IL10	GC	LRP2	GC	SPDYE8	GC
CNTNAP2	GC	UGT1A1	GC	SPDYE12P	GC
CRH	GC	ADGRA3	GC	SPDYE10P	GC
HLA-DRB1	GC	EIF3G	GC	ENSG00000266919	GC

MOG	GC	PLVAP	GC	LOC102723692	GC
REN	GC	YWHAZ	GC	SPDYE14	GC
FMR1	GC	ULK4	GC	SPDYE9	GC
ATXN3	GC	DLG2	GC	SPDYE13	GC
GRIN2B	GC	PCSK9	GC	SPDYE15	GC
PRKN	GC	MBL2	GC	LRP5L	GC
CACNA1A	GC	ALDH9A1	GC	ADAMTS12	GC
ANK3	GC	NQO2	GC	PGA4	GC
HLA-DQB1	GC	HIRA	GC	UBA52	GC
PRODH	GC	EXT2	GC	CCNG1	GC
SOD1	GC	ADCYAP1R1	GC	KLHL12	GC
NRXN1	GC	MGR1	GC	SELENOP	GC
GDNF	GC	NAALADL2	GC	MIR494	GC
TPH2	GC	ACSL4	GC	PDIA6	GC
POLG	GC	NR2F2	GC	SMC4	GC
SLC18A2	GC	CYC1	GC	PRDX2	GC
DBH	GC	MARCKS	GC	FAU	GC
MTHFR	GC	MT-TS2	GC	IL17C	GC
PHOX2B	GC	RAB3GAP1	GC	RRAGA	GC
PRL	GC	NEB	GC	RRAGB	GC
APP	GC	DLGAP2	GC	IMMP1L	GC
CHAT	GC	CALCR	GC	TMEM11	GC
UBE3A	GC	MGR3	GC	RPS19BP1	GC
DRD5	GC	MGR12	GC	PURB	GC
SLC6A2	GC	MGR8	GC	SURF4	GC
NTRK2	GC	CCNA2	GC	CNOT8	GC
SCN1A	GC	PARP1	GC	AUP1	GC
SCN2A	GC	KALRN	GC	GEMIN7	GC
PRNP	GC	WNT7B	GC	CYTL1	GC
GAD1	GC	NCAN	GC	UCN3	GC
NGF	GC	PPAN-P2RY11	GC	ACACA	GC
NR3C1	GC	NFKB1	GC	RAB28	GC
GFAP	GC	TMCO1	GC	DYTN	GC
DNMT1	GC	MACROD2	GC	WHCR	GC
CDKL5	GC	FST	GC	LRRC4C	GC
EPO	GC	ARF1	GC	PSMC3	GC
NOS3	GC	LOC102724058	GC	UGT1A	GC
HTT	GC	KIF7	GC	DKFZP434H168	GC
CCL2	GC	SEC61A1	GC	ASCL2	GC
GRIA3	GC	NES	GC	CER1	GC
GCH1	GC	IL11RA	GC	DTL	GC
PINK1	GC	SLC34A1	GC	ATP6V0E2	GC
GABRA1	GC	PICSAR	GC	ZNF620	GC
RAI1	GC	LINC00163	GC	RNASE13	GC

LRRK2	GC	LINC00165	GC	RTL10	GC
CRP	GC	INPPL1	GC	PRSS3P2	GC
SCN8A	GC	FGF7	GC	SNORD97	GC
SLC2A1	GC	MIR204	GC	DPYD-AS1	GC
SNAP25	GC	EWSR1	GC	RNASEH2B-AS1	GC
C9orf72	GC	RAB7B	GC	UNC5C	GC
KCNQ2	GC	ZBTB24	GC	ELOVL6	GC
AKT1	GC	GRIK5	GC	ROS1	GC
DISC1	GC	FIS1	GC	SLC25A16	GC
CSNK1D	GC	TDP2	GC	PTPN3	GC
IFNG	GC	CASP1	GC	ZNF622	GC
HTR1A	GC	IL17RA	GC	C17orf80	GC
VWF	GC	IL10RA	GC	PCSK6	GC
SHANK3	GC	WNT3	GC	CNN1	GC
SERPINE1	GC	GLT8D1	GC	NUDT9	GC
APOB	GC	NUP155	GC	ORMDL1	GC
F2	GC	MIRLET7I	GC	FUT1	GC
NOS1	GC	GPX1	GC	COL6A5	GC
CRY1	GC	SCZD11	GC	MIR451A	GC
GABRB3	GC	SCZD12	GC	IRAK3	GC
ICAM1	GC	SCZD13	GC	ATL2	GC
GH1	GC	SCZD14	GC	KRTCAP2	GC
RETN	GC	GREM2	GC	VSTM5	GC
CD40LG	GC	PIK3C3	GC	MIR299	GC
GABRG2	GC	PTLS	GC	PLCXD3	GC
CLOCK	GC	XPA	GC	TPTEP2-CSNK1E	GC
FGFR1	GC	IDH1	GC	RRP15	GC
GBA	GC	GYPA	GC	APOA4	GC
CHRNA7	GC	INPP1	GC	PRSS57	GC
SCN9A	GC	SLMAP	GC	MIR519D	GC
FOXP2	GC	PYGM	GC	PRD	GC
POGZ	GC	PANK1	GC	UHRF1	GC
RELN	GC	KIF15	GC	ZKSCAN3	GC
AGT	GC	HDAC1	GC	GTF2H4	GC
HTR2C	GC TTD	HSPA5	GC	NET1	GC
ATXN2	GC	CPQ	GC	MDM1	GC
GRIN1	GC	RECK	GC	SLC9B1	GC
PCDH19	GC	MIR185	GC	RPH3A	GC
PDYN	GC	EBF3	GC	CXCL2	GC
STXBP1	GC	NAV1	GC	KAT2A	GC
WFS1	GC	DAOA-AS1	GC	TXNRD1	GC
AR	GC	ARMC5	GC	FLOT1	GC
TP53	GC	RBX1	GC	LOC108660404	GC

AHDC1	GC	VSNL1	GC	MTHFD1L	GC
FOXG1	GC	ANCR	GC	GALR1	GC
FGFR3	GC	CDR1	GC	CUTC	GC
NAGLU	GC	CD38	GC	YIPF3	GC
ATP13A2	GC	CDK1	GC	MS4A4A	GC
ADAMTS2	GC	ENO1	GC	UBE2QL1	GC
ERCC6	GC	SOX5	GC	CSH2	GC
ESR1	GC	HSP90B1	GC	OR14C36	GC
TCF20	GC	MIR324	GC	OOEP	GC
DDC	GC	ATP6AP2	GC	PPRC1	GC
PMM2	GC	GPD2	GC	PCK2	GC
IQSEC2	GC	AFF4	GC	STOX2	GC
SLC1A2	GC	RAPGEF2	GC	CEND1	GC
TCF4	GC	PLCG1	GC	PTPA	GC
PSEN1	GC	MDGA1	GC	CD151	GC
MBD5	GC	GALT	GC	GIT1	GC
CREBBP	GC	CACNG5	GC	ADCY3	GC
TSC2	GC	CLCF1	GC	NIPBL-DT	GC
ADIPOQ	GC	SYN3	GC	EBF1	GC
VCP	GC	EIF4EBP1	GC	AMBRA1	GC
DEAF1	GC	FAF1	GC	UBR4	GC
TFAP2B	GC	SFTPD	GC	CERK	GC
TBP	GC	LOC109610631	GC	ARHGAP4	GC
MIR132	GC	TLR1	GC	POLD4	GC
RORA	GC	PDXK	GC	RAB4A	GC
DCTN1	GC	CSMD2	GC	SPAG17	GC
ADNP	GC	STK39	GC	CPA4	GC
SNRPN	GC	CCR7	GC	IGHG1	GC
ELN	GC	IL1RAPL2	GC	ADCY8	GC
RET	GC	UFD1	GC	SMDT1	GC
FGFR2	GC	FLAD1	GC	PACSIN3	GC
CHD8	GC	ALK	GC	MFGE8	GC
EHMT1	GC	CCNF	GC	CD3D	GC
CAT	GC	VIPR2	GC	WDR48	GC
FLNA	GC	SUN2	GC	OTX1	GC
LOC110806262	GC	MID2	GC	ABRA	GC
SYP	GC	LOC110011216	GC	IGF2BP3	GC
PARK7	GC	HTR3C	GC	ASIC2	GC
HRAS	GC	GJA8	GC	GTF3C1	GC
CHRNA4	GC	ZNF592	GC	EIPR1	GC
CASK	GC	HSPB2	GC	NAP1L5	GC
HTR3A	GC	ODC1	GC	WEE1	GC
NIPBL	GC	VCL	GC	ZNF275	GC

MMP9	GC	PMS1	GC	FIBIN	GC
NEXMIF	GC	LRIT3	GC	FAM183A	GC
CTLA4	GC	SLCO1B3	GC	LOC113960611	GC
ATXN7	GC	STIN2-VNTR	GC	MIR130A	GC
CHI3L1	GC	ALYREF	GC	ZNF214	GC
AHI1	GC	RNF19A	GC	UHRF2	GC
GRN	GC	JAKMIP1	GC	ADARB1	GC
TNFRSF1B	GC	CSTA	GC	TRPC4	GC
HLA-B	GC	PIEZO2	GC	FAM53B	GC
TLR4	GC	CTC1	GC	SEPTIN12	GC
ALG2	GC	NELFA	GC	APPL2	GC
TNFSF4	GC	KCTD17	GC	SP3	GC
TARDBP	GC	RPS15	GC	CYB5B	GC
ATN1	GC	GZMB	GC	LBP	GC
TPH1	GC	CPLX2	GC	CISD1	GC
MTOR	GC	TUBGCP5	GC	NPBWR1	GC
PRRT2	GC	DOCK8	GC	PPP1R3B	GC
VDR	GC	CPA6	GC	PRKAB2	GC
TRPV4	GC	QRICH1	GC	TCF7	GC
UCHL1	GC	CACNA1C-AS1	GC	SMC2	GC
NGLY1	GC	LOC106020709	GC	ZNF215	GC
COQ2	GC	LOC106020710	GC	GNAI1	GC
FKBP5	GC	LAPTM4A	GC	LTB	GC
LMNA	GC	LOC106627981	GC	TRPM5	GC
PDSS2	GC	OLIG2	GC	PTRH1	GC
ATXN1	GC	SPTA1	GC	MEAK7	GC
CTNNB1	GC	NPM1	GC	SLC25A5P2	GC
ARNTL	GC	UCP2	GC	TFF3	GC
BSCL2	GC	CISH	GC	LTB4R	GC
IL1RN	GC	EPHA4	GC	GNPDA2	GC
CXCL8	GC	SCYL1	GC	PLCL1	GC
IGF2	GC	ADGRL3	GC	LCORL	GC
FTL	GC	DPP10	GC	SELENOK	GC
GRM1	GC	HMCN1	GC	SMC6	GC
SLC17A5	GC	LOC108745275	GC	CDC25C	GC
IDUA	GC	LOC108745276	GC	IFIT5	GC
TGFB1	GC	ADAT3	GC	MYRIP	GC
GNRH1	GC	EMP1	GC	MUCL3	GC
GRIK2	GC	PPCS	GC	UACA	GC
SGSH	GC	HOXA13	GC	GUSBP2	GC
SCN5A	GC	SH3PXD2B	GC	WDR70	GC
PTH	GC	CFAP47	GC	NRSN1	GC
SHH	GC	ELP3	GC	MARCHF11	GC
DYNC1H1	GC	PRND	GC	SLC23A3	GC

IDS	GC	ARC	GC	DDX39B	GC
ARX	GC	EN2	GC	DMRT2	GC
MAGEL2	GC	IRAK1	GC	H2AC15	GC
MFN2	GC	SLC32A1	GC	MYSM1	GC
GNAS	GC	AQP1	GC	ERG28	GC
CP	GC	IL9	GC	CREM	GC
GABRA5	GC	DLX5	GC	AKAP13	GC
DRD1	GC	MAPT-AS1	GC	NACA	GC
SLC25A13	GC	DNAJB1	GC	SPNS3	GC
DHCR7	GC	CHRM3	GC	PLGRKT	GC
SOX2	GC	COL9A2	GC	ANO2	GC
AVP	GC	ANGPT2	GC	BICD1	GC
VEGFA	GC	FUT8	GC	SASH1	GC
NR3C2	GC	TNFRSF8	GC	TLL2	GC
SLC1A3	GC	KHDC3L	GC	ACOX3	GC
ARSA	GC	IBSP	GC	REG1A	GC
MT-ND1	GC	ITGAL	GC	OR51S1	GC
CSNK1E	GC	GUF1	GC	P3H4	GC
KRAS	GC	SHMT1	GC	CISD3	GC
ALG9	GC	LHPP	GC	MIR3911	GC
COL2A1	GC	RIT2	GC	DENND1B	GC
MTNR1A	GC TTD	BSG	GC	SMURF1	GC
HDAC4	GC	CELF4	GC	KIR2DL3	GC
PLA2G6	GC	TPPP3	GC	SSTR3	GC
SLC9A6	GC	RANGRF	GC	PTPRT	GC
PTEN	GC	UBE2N	GC	DUOXA1	GC
NPY	GC	ERMARD	GC	CENPBD1	GC
NF1	GC	SOCS3	GC	MIR367	GC
PSEN2	GC	ITIH4	GC	MIR302D	GC
HCRTR2	GC	MLX	GC	LINC01828	GC
TTR	GC	FOLH1	GC	ENSG00000234940	GC
DAOA	GC	ITGA1	GC	ENSG00000230704	GC
SLC6A1	GC	GRM8	GC	ENSG00000286145	GC
NR4A2	GC	KANK1	GC	GRDX	GC
FBN1	GC	SLC12A2	GC	LOC108281110	GC
NEFL	GC	FRZB	GC	CPA5	GC
NDUFS4	GC	MT-TQ	GC	FFAR4	GC
IL13	GC	MTMR2	GC	PAX5	GC
SYN1	GC	PPIG	GC	SLCO1C1	GC
CHKB	GC	PLXND1	GC	COLEC12	GC
APOA1	GC	F12	GC	HEG1	GC

NKX2-1	GC	FAH	GC	OSBPL5	GC
MRE11	GC	TACR2	GC	CLSTN2	GC
GPHN	GC	FKBP4	GC	P2RY2	GC
DTNBP1	GC	ADRA2C	GC	TSSC4	GC
WDR45	GC	FGF1	GC	PTDSS1	GC
NLGN4X	GC	NUP88	GC	PTPRZ1	GC
DMD	GC	AKR1A1	GC	LHX1	GC
TSC1	GC	CANX	GC	TMEM147	GC
SGCE	GC	HLA-DRB5	GC	SLC22A18AS	GC
CHD7	GC	NEAT1	GC	TIMD4	GC
NSD1	GC	GADL1	GC	LAX1	GC
PMP22	GC	FTCD	GC	BNIP3L	GC
SST	GC	CFAP43	GC	SORBS1	GC
PON1	GC	ATL3	GC	COPG2	GC
NDN	GC	PCCB	GC	TRIB1	GC
KCNQ1	GC	HOTAIR	GC	ADAM33	GC
GABRB2	GC	NLGN2	GC	PPM1H	GC
F5	GC	PRX	GC	GCM1	GC
GABBR2	GC OMIM DrugBank	PSMC4	GC	DCTPP1	GC
IL2RA	GC	PENK	GC	GLYATL1	GC
PAH	GC	BCOR	GC	BABAM1	GC
CACNA1H	GC	FNDC5	GC	PPP1R3D	GC
KCNA1	GC	CMIP	GC	SPCS2	GC
ATP1A3	GC	GABRR2	GC	PGGHG	GC
CABP4	GC	LCOR	GC	ATP11AUN	GC
SPR	GC	PLEK	GC	EMC3	GC
MTR	GC	DRC3	GC	HTR3D	GC
SCN4A	GC	LBX1	GC	PRKCH	GC
TF	GC	FABP3	GC	PRDM13	GC
SQSTM1	GC	USP9Y	GC	GRB7	GC
HLA-DQA1	GC	ZMYM2	GC	GID8	GC
NPAS2	GC	PHIP	GC	TEX28	GC
GNB3	GC	UPK3A	GC	RABL2B	GC
DLG3	GC	PECAM1	GC	C16orf87	GC
SLC1A1	GC	IVL	GC	SPRY4-IT1	GC
TSPO	GC	DAZ1	GC	NFATC2	GC
ZNF365	GC	DUSP6	GC	EFNA4	GC
AUTS2	GC	SLC19A3	GC	PDCD6IP	GC
TOR1A	GC	CPT1A	GC	MIR708	GC
GGT1	GC	DHTKD1	GC	COPA	GC
SYT1	GC	NFIA	GC	RNF138	GC

ALG6	GC	MYRF	GC	AP1S3	GC
SYNGAP1	GC	SCARB1	GC	PLLP	GC
FOS	GC	DCANP1	GC	STRN	GC
SLC6A8	GC	DBN1	GC	SMC1B	GC
HCRTR1	GC TTD	EEF1A1	GC	GPR107	GC
TBC1D24	GC	CCN2	GC	FXYD7	GC
SHANK2	GC	ENSG00000202498	GC	ZNF695	GC
CNR1	GC	DPYSL5	GC	MARVELD3	GC
ARID1B	GC	UBE2D2	GC	DSCC1	GC
MPO	GC	SEMA3E	GC	RABL2A	GC
RUNX1	GC	TARS1	GC	ENSG00000252283	GC
CRY2	GC	MYO1E	GC	TLN2	GC
PANK2	GC	CYP1B1	GC	C1orf94	GC
AGRN	GC	ADRA2B	GC	CYP4F3	GC
ACADM	GC	KIF2A	GC	RNF166	GC
HTR1B	GC	SMCR8	GC	ADH5P2	GC
EP300	GC	PSMD2	GC	NOX4	GC
EDNRB	GC	HBS1L	GC	PKD1L2	GC
GJB1	GC	AGXT	GC	VPS52	GC
FUS	GC	MIR181A1	GC	ADPRH	GC
HMOX1	GC	TLK2	GC	TPRN	GC
ALG13	GC	SEC24C	GC	AKAP6	GC
MAOB	GC	MARS1	GC	CAMK1D	GC
DISC2	GC	LORICRIN	GC	MBD3	GC
NPPA	GC	GBA2	GC	PTPRS	GC
MAN1B1	GC	LAMB1	GC	PASK	GC
VPS13A	GC	MIEF2	GC	GSDMB	GC
TFRC	GC	CYB5R3	GC	TMEM14A	GC
BRAF	GC	DCC	GC	GUSBP15	GC
BRCA2	GC	CCKBR	GC	BAAT	GC
KCNQ3	GC	CNTFR	GC	SLC8A1	GC
NLGN3	GC	IL12B	GC	FGF19	GC
CYBB	GC	FOXO3	GC	LIME1	GC
HADHA	GC	TOX3	GC	FBXO15	GC
TTN	GC	HEXB	GC	OFCC1	GC
SLITRK1	GC	IRF4	GC	INTS3	GC
HFE	GC	SMIM30	GC	ODAM	GC
NAGS	GC	MLH3	GC	OARD1	GC
PNKD	GC	GPR37	GC	TMEM128	GC
MYH9	GC	DEL1Q21	GC	SVBP	GC
TLR2	GC	LOC108660406	GC	DTD1	GC
SCN3A	GC	RREB1	GC	FAF2	GC

COL1A1	GC	AFG3L2	GC	LOC109611589	GC
NPC1	GC	ERDA1	GC	MIR19B1	GC
PLP1	GC	COX5A	GC	USP25	GC
DMPK	GC	VGF	GC	TOP1MT	GC
P2RY11	GC	PSMD3	GC	CCR10	GC
MPI	GC	APEX1	GC	ASPG	GC
NOTCH1	GC	SGK1	GC	OST4	GC
MEN1	GC	GNA11	GC	GUSBP4	GC
DPP6	GC	CD63	GC	MIR6084	GC
MC4R	GC	CDR2	GC	NOSTRIN	GC
TAC1	GC	NOG	GC	CXADR	GC
MKRN3	GC	KLHL24	GC	CERS6	GC
SERPINC1	GC	ARHGAP24	GC	DCDC2B	GC
CDK5	GC	SKOR1	GC	TMC5	GC
GABRD	GC	CHRNB4	GC	ATRNL1	GC
PAX6	GC	DYNC2H1	GC	RPN1	GC
NOS2	GC	SCAP	GC	NRF1	GC
U2AF1	GC	TET1	GC	ARL6IP5	GC
HERC2	GC	SERPINF1	GC	RHOF	GC
RUNX2	GC	H3-2	GC	MAK16	GC
ATRX	GC	SBF2	GC	SMIM23	GC
MMP2	GC	SPRY2	GC	KCNA6	GC
COL3A1	GC	PCSK1	GC	SLC35F4	GC
HCN1	GC	CARTPT	GC	DUX4L1	GC
SLC5A7	GC	NT5M	GC	FAM149A	GC
FA2H	GC	LINC02153	GC	STPG3	GC
KCNH2	GC TTD	ADCY1	GC	WFDC21P	GC
SYNJ1	GC	MIR26B	GC	LINC00976	GC
CAV3	GC	CIC	GC	RACK1	GC
TAF1	GC	RAB18	GC	TYW5	GC
TG	GC	SLC9A3	GC	F2RL2	GC
PCLO	GC	ITSN2	GC	RAD54L2	GC
SNCAIP	GC	MYO15A	GC	C8orf37-AS1	GC
OXT	GC	LOC106050102	GC	L3MBTL4	GC
TYR	GC	RBM4	GC	GTF2H1	GC
CDH23	GC	AFG1L	GC	PXDNL	GC
HGSNAT	GC	SEMA4A	GC	TIMM17A	GC
CTSD	GC	NUDT1	GC	CBLB	GC
EGF	GC	CYSLTR1	GC	ING3	GC
PPT1	GC	SORCS2	GC	SLC25A40	GC
MEF2C	GC	CAMP	GC	MTCL1	GC
AANAT	GC	CORT	GC	DUSP3	GC
MPZ	GC	CD14	GC	SLC22A2	GC
SRPX2	GC	GRID1	GC	PPP1R27	GC

GNS	GC	MIR146B	GC	IMMT	GC
GNAO1	GC	RPS6	GC	DCT	GC
PSAP	GC	LIPC	GC	GOLIM4	GC
TPO	GC	ARHGEF6	GC	SLC9A8	GC
ITGB3	GC	PTX3	GC	TCF15	GC
SMC3	GC	FH	GC	MLLT10	GC
PTPN11	GC	BECN1	GC	TP53RK	GC
BHLHE41	GC	NMB	GC	CAMK4	GC
EPRS1	GC	TRMT10C	GC	TRA	GC
ADORA2A	GC	DCLK1	GC	NOP9	GC
TUSC3	GC	IGF2BP2	GC	RPL3	GC
SOX3	GC	RIC3	GC	BMP8B	GC
ALG11	GC	HNRNPUL2-BSCL2	GC	NSMCE4A	GC
CHMP2B	GC	PCDH15	GC	YIPF6	GC
SMPD1	GC	ST8SIA2	GC	ZUP1	GC
CACNB2	GC	CAPN2	GC	KPNA7	GC
FAS	GC	IKZF3	GC	RAPGEF6	GC
KDM5C	GC	YWHAB	GC	MCC	GC
HMBS	GC	MYL1	GC	SLC24A2	GC
SLC9A7	GC	FYN	GC	VPS50	GC
CALR	GC	KIF3A	GC	ZWINT	GC
CYP2D6	GC	RBMY1A1	GC	KANSL1-AS1	GC
ASCL1	GC	CADPS2	GC	SLC29A4	GC
BMP2	GC	PSMD14	GC	TXNDC2	GC
MED12	GC	SLC1A7	GC	EPHA1	GC
GNAL	GC	OMD	GC	RBFOX2	GC
SMARCA2	GC	CCND2	GC	HLA-S	GC
LDLR	GC	PARS2	GC	TFAP2C	GC
IREB2	GC	STRADA	GC	CYP39A1	GC
NTRK1	GC	SPON1	GC	IDNK	GC
CLCN2	GC	KCNK5	GC	DUSP26	GC
TNFRSF1A	GC	MOBP	GC	TMEM134	GC
RAB39B	GC	TMEM138	GC	ABHD14A	GC
SPTAN1	GC	LRRTM1	GC	ABHD18	GC
MIR21	GC	HDAC2	GC	CCDC177	GC
PTCHD1	GC	SIGLEC5	GC	ZIM2-AS1	GC
PRPS1	GC	MAPRE3	GC	SLC2A1-AS1	GC
GHR	GC	VPS51	GC	MESTIT1	GC
KCNT1	GC	PHGDH	GC	LRIG2	GC
CBL	GC	PIKFYVE	GC	IRX1	GC
H2AC18	GC	TLX3	GC	PLPPR5	GC
NPC2	GC	PTCH2	GC	FHAD1	GC
RBFOX1	GC	IL4R	GC	WSCD2	GC

CHRNB2	GC	LIG3	GC	CD180	GC
GNE	GC	PIR	GC	DPEP1	GC
RYR2	GC	TOMM40	GC	TLL1	GC
ADRB2	GC TTD	ARTN	GC	CSNK1A1	GC
HLA-DPB1	GC	DEL15Q11.2	GC	CCDC136	GC
STAT3	GC	LINC01108	GC	PKP4	GC
IL2	GC	LINC02152	GC	RND3	GC
GABBR1	GC DrugBank	CNNM2	GC	CBY1	GC
MT-ATP6	GC	KLF4	GC	DDR1	GC
GRIA4	GC	UTRN	GC	STK4	GC
GABRA2	GC	EEA1	GC	SERPINA12	GC
TPP1	GC	ACMSD	GC	BTC	GC
HNMT	GC	COPS3	GC	KIR2DL4	GC
CACNB4	GC	KLK6	GC	ADAMTS16	GC
GLA	GC	RNF2	GC	HSD17B1	GC
CLN5	GC	ADRA1D	GC	TMCO4	GC
HSPD1	GC	TLR7	GC	TCFL5	GC
DPAGT1	GC	VIPR1	GC	MIR302CHG	GC
GJA1	GC	GRIK3	GC	ANAPC1	GC
FN1	GC	DDIT3	GC	IFI16	GC
TRAPPC9	GC	TIMP2	GC	LINC01194	GC
SRC	GC	EHMT2	GC	QTRT1	GC
PNKP	GC	HLA-DRA	GC	PRELP	GC
MT-CO1	GC	KDSR	GC	CSAG1	GC
MAG	GC	CS	GC	GOPC	GC
MT-ND5	GC	PMCH	GC	PSMD8	GC
CREB1	GC	PCDH11X	GC	OSMR	GC
DHX30	GC	LCN2	GC	BTRC	GC
MT-TL1	GC	GLRA2	GC	KCNMB1	GC
HSPG2	GC	APELA	GC	APCS	GC
SPG11	GC	GLS	GC	ITPK1	GC
FRRS1L	GC	ARHGAP11A	GC	NGB	GC
SLC18A3	GC	CRKL	GC	PRDM4	GC
KCNJ11	GC	FCER2	GC	LGALS13	GC
RERE	GC	LINC02694	GC	CHRAC1	GC
TSHR	GC	IER3	GC	SAMD8	GC
MOGS	GC	CXCL1	GC	MRXSL	GC
MAP2K1	GC	PRDM12	GC	APBB2	GC
CASR	GC	CAMTA1	GC	ADORA3	GC
DAO	GC	NUP93	GC	ENTPD5	GC
CLN3	GC	TUBB8	GC	TTC9B	GC
ATXN8OS	GC	CHERP	GC	ZNF428	GC

ABCA1	GC	LOC106020711	GC	TICAM1	GC
XBP1	GC	LOC106020712	GC	NAA20	GC
KMT2A	GC	MDH1	GC	SLC2A13	GC
ECE1	GC	KDR	GC	HSD3B1	GC
ALDH5A1	GC	PSMD9	GC	TIPIN	GC
CYP19A1	GC	BBS12	GC	PTGFR	GC
PWRN1	GC	FCGR3A	GC	TWISTNB	GC
FOXP3	GC	TOR1B	GC	PRTFDC1	GC
NKX2-5	GC	MCL1	GC	CA6	GC
GALNS	GC	KIF1C	GC	SMAD7	GC
VPS35	GC	AS3MT	GC	DIAPH3	GC
CYP17A1	GC	PNMA2	GC	PELI2	GC
MYH7	GC	VTN	GC	CERS5	GC
VPS13B	GC	PACRG	GC	PLEKHA8	GC
EGR2	GC	SRR	GC	CCPG1	GC
LTA	GC	MIR328	GC	ARHGAP36	GC
FGF8	GC	FTMT	GC	OR5AS1	GC
COG2	GC	CCL18	GC	TMEM266	GC
OPN4	GC	SORCS1	GC	DBH-AS1	GC
IGFBP3	GC	ALDH1A1	GC	CADM3-AS1	GC
SOX10	GC	MESD	GC	PAFAH1B1P2	GC
OPHN1	GC	CYBA	GC	ARHGAP1	GC
PROP1	GC	ACTC1	GC	ACOX2	GC
HTRA2	GC	CFL1	GC	PSG1	GC
PLAU	GC	DDHD2	GC	SLC28A1	GC
P2RX7	GC	PSMD5	GC	SLC3A2	GC
NIPA1	GC	LYN	GC	TMEM18	GC
CASP8	GC	RPS6KB2	GC	PEG13	GC
ACHE	GC	CD86	GC	ANKRD44	GC
CFI	GC	CELF1	GC	TUBGCP6	GC
GIGYF2	GC	IL33	GC	FREM3	GC
CACNA1S	GC	RPS6KB1	GC	ZNRD1	GC
FKRP	GC	PPIEL	GC	NPFFR1	GC
CYP21A2	GC	GATA3	GC	ACTR5	GC
SERPINA1	GC	MIR27A	GC	YEATS4	GC
FLCN	GC	NSF	GC	OTOG	GC
COL1A2	GC	BTBD16	GC	CSNK1A1L	GC
QDPR	GC	NRP1	GC	UFL1	GC
KCNA2	GC DrugBank	RIN2	GC	PIN1	GC
SMARCB1	GC	ARRB2	GC	PCMT1	GC
PROK2	GC	HSD17B3	GC	GTDC1	GC
SELP	GC	DENND5A	GC	CHD9	GC

THRB	GC	TAT	GC	ATP10B	GC
FXN	GC	FGF9	GC	HCAR2	GC
SLC35A1	GC	DRG2	GC	ENTPD4	GC
MT-TK	GC	RGS2	GC	BAZ1A	GC
COL5A1	GC	DAPK1	GC	PPARGC1B	GC
SCN1B	GC	TJP1	GC	TUSC1	GC
HPRT1	GC	FGD4	GC	SUPT16H	GC
GPT	GC	PLA2G2A	GC	TRAPPC12	GC
SETD5	GC	ALDH3A1	GC	DOC2A	GC
RAPSN	GC	ITGAX	GC	DCTD	GC
BRCA1	GC	LLGL1	GC	SEMA6D	GC
APC	GC	EXT1	GC	LOC101927066	GC
TBX1	GC	TENT5A	GC	TANC2	GC
ADRB1	GC	CYB561	GC	TFF2	GC
KIF1A	GC	ATG4C	GC	NPY2R	GC
LPL	GC	RPS20	GC	MAP9	GC
OPRM1	GC	VAMP7	GC	IL21-AS1	GC
POU1F1	GC	LRRK1	GC	CD3G	GC
GLUD1	GC	TAF6	GC	ELFN1	GC
SMC1A	GC	VDAC1	GC	ASCL3	GC
ALS2	GC	MICB	GC	UNK	GC
MUSK	GC	SETDB1	GC	WDYHV1	GC
ABAT	GC	INSL3	GC	NDUFA5	GC
SELENON	GC	POSTN	GC	DSEL	GC
CC2D2A	GC	SH2B1	GC	SLC25A2	GC
NRG1	GC	TBX3	GC	HLA-DRB4	GC
ASPM	GC	MAFA	GC	OR6P1	GC
CRHR1	GC	SRF	GC	ARHGEF40	GC
SEMA3A	GC	CTSG	GC	RHBDF1	GC
LEPR	GC	CADM1	GC	CCHCR1	GC
SLC25A22	GC	SULT2A1	GC	C19orf33	GC
SPG7	GC	TLR10	GC	NUCB2	GC
SUMF1	GC	DMRT1	GC	ECI2	GC
NALCN	GC	KCNH5	GC	RFTN2	GC
KCNJ2	GC	NBEA	GC	CYLC2	GC
CHRNA2	GC	TRPM8	GC	RAP2C	GC
HLA-A	GC	TCF12	GC	METTL7B	GC
PIK3CA	GC	H2AC20	GC	SLC25A41	GC
ADORA1	GC	CDH10	GC	CCDC70	GC
CTCF	GC	TINF2	GC	LIPG	GC
NPS	GC	PDLIM1	GC	TMEM132B	GC
HSPB1	GC	MTMR10	GC	TEC	GC
SRD5A3	GC	MIR326	GC	RFX4	GC
ASPA	GC	TXNDC15	GC	RPL13	GC

ABCB7	GC	ID2	GC	CBX5	GC
ATXN10	GC	OLR1	GC	CLYBL	GC
VPS13C	GC	ENSG00000277767	GC	FABP1	GC
FOXP1	GC	MCAM	GC	DDRGK1	GC
ADH1C	GC	GPER1	GC	METTL21C	GC
CFTR	GC	HTR3E	GC	PTGER2	GC
NEFH	GC	WDPCP	GC	DYNLT1	GC
CLCN1	GC	ARHGAP11B	GC	ERC2	GC
S100B	GC	PWARSN	GC	CEP128	GC
SLC25A1	GC	RAD23A	GC	LGR6	GC
ANK2	GC	TLR8	GC	PLEKHH2	GC
THBD	GC	HIP1R	GC	TMEM184B	GC
HDC	GC	SMARCA1	GC	FBXL18	GC
DEPDC5	GC	DLX1	GC	DPH3	GC
LMX1B	GC	NR4A1	GC	FBXO48	GC
SNORD115-1	GC	NREP	GC	MIR639	GC
MT-CYB	GC	CLEC16A	GC	CPEB1-AS1	GC
STS	GC	PHF1	GC	LOC107181287	GC
PTS	GC	ARHGEF15	GC	APOM	GC
STAT1	GC	PDE11A	GC	APEH	GC
PAFAH1B1	GC	MBNL1	GC	HSP90AB2P	GC
ATP7A	GC	TRIM21	GC	MROH6	GC
GSN	GC	ACP1	GC	LINC00051	GC
NPPB	GC	SRGAP2	GC	GNLY	GC
F8	GC	PUS3	GC	GALR3	GC
SERPINA3	GC	P2RX4	GC	SEC23IP	GC
SOX9	GC	BRAT1	GC	NMU	GC
GABRA3	GC TTD	SYK	GC	LINC02210	GC
SATB2	GC	FHL5	GC	LILRB1	GC
NR1D1	GC DrugBank	WHRN	GC	ACSL3	GC
NR5A1	GC	SYNE2	GC	LOC109623489	GC
SYT2	GC	GGTLC3	GC	MARCHF6	GC
ARSB	GC	RASD1	GC	TMSB4X	GC
MFSD8	GC	SMCR5	GC	PIGF	GC
FKTN	GC	MEG8	GC	UBQLNL	GC
MYH6	GC	TUBB	GC	RTN1	GC
TNFRSF11B	GC	FCRL3	GC	PPP2R3A	GC
CACNA1B	GC	GRIN2C	GC	LINC02288	GC
ZEB2	GC	HTR1E	GC	EHD3	GC
RAB7A	GC	MIEF1	GC	SPCS3	GC
CPT2	GC	GYPC	GC	DOCK1	GC

SLC11A2	GC	EGR1	GC	ATP8B3	GC
BTD	GC	IRF1	GC	KPNA3	GC
RPS6KA3	GC	EPB41	GC	SLC28A2	GC
TACR1	GC	CLCN6	GC	MIR452	GC
WASHC5	GC	PARL	GC	HAGH	GC
MSX1	GC	POU3F3	GC	PPM1B	GC
CST3	GC	DLX6	GC	UBE2D4	GC
PRKCG	GC	AIM2	GC	SCAMP5	GC
TWNK	GC	LINGO2	GC	FCRL4	GC
KAT6B	GC	GGT2	GC	ZCCHC24	GC
HESX1	GC	ANXA2	GC	PATJ	GC
MT-ND6	GC	CALCRL	GC	DYNC1I1	GC
DYRK1A	GC	LMAN1	GC	C12orf50	GC
AFF2	GC	PRG2	GC	SPAG16	GC
ANKRD11	GC	CD44	GC	IFNGR2	GC
SLC26A4	GC	ARFGAP1	GC	CD109	GC
MDM2	GC	POU5F1	GC	KCNN2	GC
OTX2	GC	RAC1	GC	FOXN3	GC
CLN6	GC	SLC2A3	GC	SLC22A14	GC
SYN2	GC	CA8	GC	ANKRD60	GC
HAMP	GC	P2RX3	GC	AHRR	GC
EIF4G1	GC	GSTO1	GC	NANOG	GC
F9	GC	ALKBH5	GC	CDH11	GC
FLII	GC	PGD	GC	CACNA2D3	GC
SLC12A3	GC	PTPRO	GC	MELTF	GC
F10	GC	FRMPD4	GC	UBA7	GC
ST3GAL3	GC	NAA50	GC	PECR	GC
SPAST	GC	ADIPOR2	GC	GNG5	GC
H19	GC	PRKACG	GC	FAM126B	GC
CCR1	GC	CUL1	GC	PSORS1C1	GC
MYO9A	GC	SPTB	GC	PXK	GC
LOX	GC	AKR1C2	GC	HTRA4	GC
KMT2D	GC	STBD1	GC	C15orf39	GC
GRM5	GC	IL31	GC	GUCY1A2	GC
AQP4	GC	NEDD4L	GC	SDK1	GC
C3	GC	DUPD1	GC	GP2	GC
EPM2A	GC	RXRB	GC	SGCZ	GC
FASLG	GC	CCL22	GC	IFI44	GC
PNPO	GC	WRNIP1	GC	CCN3	GC
DOK7	GC	CBFB	GC	ATIC	GC
GLUL	GC	UBN2	GC	WWC1	GC
GJB2	GC	PTPRN	GC	ANP32A	GC
CTSH	GC	ELAVL4	GC	RAD51B	GC
PLG	GC	FEV	GC	PACSIN1	GC

CLCNKB	GC	MUC5AC	GC	URM1	GC
FAN1	GC	CACNA1I	GC	PLAC1	GC
STAG2	GC	GRM4	GC	PCDHGB3	GC
VAMP1	GC	SUCLG1	GC	LINC01094	GC
MIR22	GC	GLRX5	GC	SRI	GC
CCK	GC	GALNT17	GC	AK2	GC
B2M	GC	ALS3	GC	ADAM8	GC
FLNB	GC	KCTD13	GC	HPSE	GC
DNA2	GC	KLRK1	GC	TCF19	GC
SPP1	GC	NDRG1	GC	NEBL	GC
C19orf12	GC	HPGDS	GC	THOC1	GC
SNCB	GC	CALY	GC	ADCK1	GC
GAMT	GC	SGCD	GC	PSORS1C2	GC
PCNT	GC	DLEU2	GC	NUP153	GC
ATP2A2	GC	CTSL	GC	ARRB1	GC
HDAC8	GC	GRIN3A	GC	KBTBD8	GC
SYNE1	GC	HSPE1	GC	SOCS6	GC
AGTR1	GC	NR1I2	GC	MYO10	GC
PROKR2	GC	ARFGAP3	GC	MT2A	GC
TGFBR2	GC	YUHAL	GC	GRK5	GC
HBB	GC	USP6	GC	CPVL	GC
CDKN1C	GC	NCS1	GC	FER	GC
IL7	GC	REL	GC	SLC4A7	GC
NHLRC1	GC	HSD17B12	GC	CDK5R1	GC
THAP1	GC	CNP	GC	TPP2	GC
SLC35A2	GC	CCL26	GC	CCDC33	GC
FBXO7	GC	HDAC3	GC	KCTD8	GC
CLN8	GC	VARS1	GC	BCL2L13	GC
MAP2K2	GC	UBQLN1	GC	NPFFR2	GC
RAD21	GC	NT5C1A	GC	SLC25A31	GC
ZNF804A	GC	SSTR5	GC	RABIF	GC
VCAM1	GC	GJD2	GC	SENP7	GC
AQP2	GC	H4-16	GC	NETO2	GC
CD36	GC	UROS	GC	OR52B2	GC
PDGFRB	GC	NPPC	GC	NPBWR2	GC
FLI1	GC	KCNJ12	GC	CYHR1	GC
DNAJC13	GC	FEZ1	GC	MS4A6E	GC
GAD2	GC	TEK	GC	NUDT17	GC
ARHGEF9	GC	PRIMA1	GC	DGAT2L6	GC
NPHP1	GC	MMP8	GC	FAM187B	GC
ZNF41	GC	RECQL	GC	OR5K1	GC
GLB1	GC	RPA1	GC	PLAC8L1	GC
PTPN22	GC	APH1A	GC	FAM228A	GC
SCN4B	GC	GNB1L	GC	OR2T4	GC

KANSL1	GC	PEG3	GC	MTRNR2L3	GC
CHRNB1	GC	PTPRN2	GC	C8orf17	GC
FGF10	GC	MT3	GC	DLEU1-AS1	GC
IL4	GC	TOMM20	GC	YWHAEP7	GC
MCOLN1	GC	RTL1	GC	MIR4325	GC
LAMA2	GC	WNT2	GC	MIR4539	GC
ATP1A2	GC	OGG1	GC	LOC101927055	GC
IL2RB	GC	DST	GC	CEACAMP8	GC
SCN11A	GC	SCGB1A1	GC	FLT1P1	GC
HRH3	GC	TOP2B	GC	RASSF1	GC
DPYD	GC	BCL2A1	GC	MAGEA9	GC
TSPYL1	GC	RNF112	GC	CDC42EP3	GC
WASHC4	GC	MIR338	GC	IL37	GC
PDGFB	GC	MLC1	GC	PDZD2	GC
G6PC	GC	THBS1	GC	RNF185	GC
NTF3	GC	RPL38	GC	NALCN-AS1	GC
NSUN2	GC	USP27X	GC	INMT	GC
NDP	GC	WASHC1	GC	MT1F	GC
XK	GC	LINC-ROR	GC	DCP1B	GC
GUSB	GC	TSLP	GC	KPNB1	GC
GRIN2D	GC	GLP1R	GC	AOC1	GC
TRIM8	GC	MIR485	GC	POLL	GC
SPG21	GC	NRCAM	GC	ENSG00000286192	GC
LAMB2	GC	COQ4	GC	MALRD1	GC
THPO	GC	ATG12	GC	UBXN7	GC
RBFOX3	GC	APOA5	GC	LINC00243	GC
DNM1	GC	ALS7	GC	RASSF8	GC
TWIST1	GC	SUCLA2	GC	MYO3A	GC
NDUFV2	GC	RPS6KA1	GC	RHOT1	GC
CYP11A1	GC	NBEAL1	GC	SNX7	GC
CHRNE	GC	SLC7A5	GC	MDM4	GC
LAMP2	GC	PRKACA	GC DrugBank	MAIP1	GC
COLQ	GC	SMAD2	GC	CPSF7	GC
SLC6A5	GC	SULT4A1	GC	TCTA	GC
RPS27A	GC	SORT1	GC	BAIAP2L1	GC
IL17A	GC	MIA2	GC	DCHS2	GC
GDAP1	GC	TTN-AS1	GC	GPR139	GC
WT1	GC	SLC2A4	GC	BSN	GC
CRYAB	GC	ADIPOR1	GC	MNDA	GC
FBN2	GC	CGAS	GC	CWC27	GC
ACADS	GC	GLIS3	GC	ARL4A	GC

GATA4	GC	GRD1	GC	BRMS1L	GC
ESR2	GC	ALLC	GC	HDX	GC
RSRC1	GC	PRSS3	GC	CDC14C	GC
ADSL	GC	NAPB	GC	NDUFA8	GC
SUFU	GC	CLINT1	GC	SP110	GC
NOTCH3	GC	FURIN	GC	NRN1	GC
DNAJC6	GC	NAGPA	GC	HLF	GC
TNNT2	GC	RAB10	GC	AK5	GC
IQCB1	GC	UNG	GC	SPECC1	GC
SLC12A5	GC	PPIP5K2	GC	ATRN	GC
PITX2	GC	MRPS22	GC	LPAR1	GC
CNKSR2	GC	NCAPD2	GC	F8A1	GC
PHOX2A	GC	CAMK2G	GC	LINC01122	GC
GSK3B	GC	ITGA4	GC	ACKR2	GC
CACNA1D	GC	BAMBI	GC	PGRMC1	GC
SIN3A	GC	LMO1	GC	LOC643542	GC
GLRB	GC	TUBA1B	GC	PKD2L1	GC
OXTR	GC	CDH8	GC	ZBTB17	GC
LHX3	GC	FGFR1OP	GC	SLC12A4	GC
ANK1	GC	IRF2BP2	GC	GTPBP1	GC
L1CAM	GC	OMP	GC	COP1	GC
G6PD	GC	KCNK18	GC	PKN3	GC
PTCH1	GC	FXYD6	GC	MIR1908	GC
MC1R	GC	RHCE	GC	HSPA14	GC
PITX3	GC	ZNF750	GC	TFPT	GC
GARS1	GC	CHL1	GC	ZKSCAN8	GC
KDM5B	GC	MARK3	GC	ZNF446	GC
ADCYAP1	GC	ADGRV1	GC	UGT2B7	GC
TNFSF11	GC	ACD	GC	TMEM115	GC
SLC52A2	GC	SLC5A6	GC	KCNS2	GC
LRP5	GC	ETAA1	GC	ZFP3	GC
EDN3	GC	ZNF385D	GC	ZNF133	GC
FGA	GC	BUD23	GC	UBR2	GC
C12orf4	GC	MLEC	GC	PARD3B	GC
TBR1	GC	SNIP1	GC	NCAM2	GC
AIRE	GC	CHGB	GC	PPP1R12B	GC
GCG	GC	PLXNA2	GC	CD99	GC
PEX5	GC	EPX	GC	FOXO6	GC
CALCA	GC	POU3F2	GC	HIVEP3	GC
MIR17	GC	PRDM9	GC	ZNF662	GC
CCR5	GC	NCOA7	GC	LTC4S	GC
GAL	GC	PRSS1	GC	VTA1	GC
TNFRSF13B	GC	CASP2	GC	PCDH7	GC
ARNT2	GC	GRB2	GC	LGSN	GC

USP8	GC	MBS2	GC	DVL1P1	GC
PER1	GC	MBS3	GC	CEP350	GC
GATA1	GC	ATOD1	GC	SPATA8	GC
PAX8	GC	ATOD3	GC	FMOD	GC
BTBD9	GC	ATOD5	GC	IK	GC
FIG4	GC	ATOD6	GC	ABCF1	GC
EPG5	GC	DUP7Q11.23	GC	FLRT2	GC
PROC	GC	ATOD7	GC	ENPP6	GC
ATP2B3	GC	ATOD8	GC	KNTC1	GC
ELANE	GC	ATOD9	GC	ANKMY1	GC
TREX1	GC	WAPL	GC	FTCDNL1	GC
EIF4E	GC	ATP2C2	GC	SERPINA4	GC
COL13A1	GC	SRGAP3	GC	KDM3B	GC
IPW	GC	BTNL2	GC	SPRED2	GC
TBX5	GC	MIAT	GC	NR1D2	GC
EGFR	GC	MIR195	GC	EDN2	GC
ASS1	GC	CD55	GC	AGBL2	GC
PLCB4	GC	VPS26A	GC	INKA1	GC
FBXL3	GC	ZDHHC8	GC	TENT4A	GC
IRS1	GC	FGGY	GC	AZIN2	GC
CD28	GC	PAGR1	GC	SVEP1	GC
DNAAF4	GC	SLC41A1	GC	ZPR1	GC
ZFYVE26	GC	FCSK	GC	SLCO3A1	GC
ACTA1	GC	SLC5A2	GC	FAM107B	GC
NSDHL	GC	AHR	GC	MATK	GC
GAA	GC	CFHR2	GC	SRL	GC
EFHC1	GC	SLITRK5	GC	FABP5	GC
COASY	GC	IFNAR1	GC	EPB41L3	GC
ERBB4	GC	SEMA3C	GC	COL28A1	GC
C4A	GC	KRT8	GC	ARHGEF3	GC
GRIA1	GC	KCNQ1-AS1	GC	STAC	GC
PDE4D	GC	COL4A2	GC	PZP	GC
ELP4	GC	DNAJC12	GC	HAS2	GC
MMD	GC	HS1BP3	GC	ZFP36	GC
SCN10A	GC	SCN7A	GC	SLC24A3	GC
SETBP1	GC	LIN7B	GC	ABLIM1	GC
ATP6V0A2	GC	ANLN	GC	RGS7	GC
GDI1	GC	ARID2	GC	HUS1	GC
PRKAR1A	GC	UTS2	GC	KSR2	GC
AIFM1	GC	USH1C	GC	PDF	GC
SPART	GC	SEPTIN4	GC	NICN1	GC
KCNB1	GC	HEY2	GC	ZNF292	GC
IGF1R	GC	SRXN1	GC	SRSF10	GC
NLRP3	GC	ACER3	GC	AK1	GC

GLI3	GC	KMO	GC	USP4	GC
PTPRC	GC	IMPACT	GC	UBAP2L	GC
DNAJC5	GC	HNRNPH1	GC	HSPD1P15	GC
KCNMA1	GC	IFT20	GC	ANKAR	GC
TET2	GC	KCNIP2	GC	HCG18	GC
GABRR3	GC	SSTR1	GC	RIF1	GC
NDE1	GC	CCT3	GC	PLA2G2D	GC
TCOF1	GC	MMP12	GC	CYP2J2	GC
POMGNT1	GC	CCL27	GC	NMUR1	GC
SETX	GC	SERPING1	GC	SYT6	GC
UBE2L3	GC	ADK	GC	AMOTL2	GC
GLRA1	GC	EPHA3	GC	TICRR	GC
CSTB	GC	CELF2	GC	ANXA6	GC
DKK1	GC	H3C14	GC	ACACB	GC
KCNE1	GC	CLPS	GC	MON1A	GC
EDNRA	GC	ALKBH8	GC	GLB1L3	GC
INSR	GC	NR1I3	GC	H3-3B	GC
SH3TC2	GC	CIDEC	GC	TLN1	GC
TIMM8A	GC	NAMPT	GC	PRM1	GC
KITLG	GC	CYP46A1	GC	ACTBL2	GC
USP9X	GC	ACVR1	GC	ACTG1P22	GC
SNTA1	GC	C1orf210	GC	ENSG00000226965	GC
WASF1	GC	ZIC1	GC	FECHP1	GC
MYC	GC	GMDS	GC	ROMO1	GC
KIAA0319	GC	MIR23A	GC	ENSG00000249881	GC
TSHB	GC	GPM6B	GC	PBX2P1	GC
HUWE1	GC	SPRN	GC	MIR2113	GC
MAP1B	GC	POLI	GC	ENSG00000257060	GC
SPTBN2	GC	SHTN1	GC	ENSG00000262267	GC
FGF23	GC	TLX2	GC	MRPL35	GC
HIF1A	GC	CLNK	GC	MTERF1	GC
IL12RB1	GC	RNF168	GC	NFAT5	GC
TENM4	GC	PSPN	GC	MSRB2	GC
SNORD116-1	GC	GJC1	GC	CPNE8	GC
DNMT3B	GC	DISP1	GC	GPX6	GC
TFAM	GC	SELENBP1	GC	SHISA4	GC
PVALB	GC	FMN1	GC	ATP6V0B	GC
GNB5	GC	SDC1	GC	MIR3681HG	GC
FAR1	GC	FBXL13	GC	CELA1	GC
MIR126	GC	SPI1	GC	SUPT3H	GC

ABCC8	GC	ITGB1	GC	KCTD16	GC
HOXA1	GC	HSPB7	GC	AGMO	GC
KCNE2	GC	TRAP1	GC	SLC7A4	GC
CEP57	GC	AHSG	GC	IRF2	GC
TIMELESS	GC	FGF20	GC	RAD18	GC
MIR140	GC	BAK1	GC	KLHL29	GC
CD2AP	GC	HOXB5	GC	MVB12B	GC
ALG1	GC	LRRC43	GC	IGSF9B	GC
MYH11	GC	MCM6	GC	SLC22A8	GC
SHBG	GC	HHEX	GC	SMYD3	GC
RHO	GC	VARS2	GC	C15orf32	GC
JRK	GC	MYO1D	GC	ADAMTS19	GC
LTBP3	GC	SLC27A5	GC	BAG6	GC
LEPQTL1	GC	SNHG1	GC	SMAD5	GC
FLT1	GC	LINC01554	GC	STIM2	GC
PRKAG2	GC	MIR122	GC	GJB5	GC
LHX4	GC	NEDD9	GC	ROR1	GC
CSF1R	GC	CD68	GC	PAK5	GC
IGLON5	GC	CTBP1	GC	SRRM4	GC
TSPAN7	GC	MTF1	GC	LOC101927284	GC
STAR	GC	PLEKHB1	GC	TMC2	GC
IGFBP1	GC	NEFM	GC	IGKV2D-29	GC
CDC42	GC	PTRHD1	GC	MIR664A	GC
IL1RAPL1	GC	ANGPTL8	GC	CWF19L2	GC
OGDH	GC	MIR154	GC	PLEKHG1	GC
CLIP2	GC	EN1	GC	STXBP4	GC
RS1	GC	HNRNPDL	GC	FAM216A	GC
NR0B1	GC	ACE2	GC	MIR99AHG	GC
GTF2I	GC	AP4E1	GC	H1-5	GC
PLCB1	GC	AGFG1	GC	AHNAK	GC
ITPR1	GC	DDB1	GC	ASGR2	GC
SCP2	GC	PTK2B	GC	ACTR3	GC
GJC2	GC	SBF1	GC	GAS2	GC
CNBP	GC	CAMKK2	GC	ENAH	GC
DNM2	GC	PWAR5	GC	CCDC91	GC
ADAM10	GC	INPP5B	GC	KCNIP4	GC
CAMK2A	GC	MIR33A	GC	HCG4	GC
TGFB2	GC	CMYA5	GC	HLA-K	GC
CHRND	GC	GRB10	GC	MICD	GC
CTSA	GC	CXorf56	GC	UFL1-AS1	GC
SHOX	GC	TFG	GC	DDR1-DT	GC
FXYD1	GC	ALAS1	GC	ENSG00000271860	GC
JPH3	GC	NEDD8	GC	RF00017-5399	GC

CCL11	GC	TAOK2	GC	CARS1P2	GC
BMP6	GC	SLITRK2	GC	ENSG00000226066	GC
MYOD1	GC	PPM1G	GC	SLC8A1-AS1	GC
DCAF17	GC	CYBRD1	GC	FSTL1	GC
XDH	GC	PML	GC	AK8	GC
CPOX	GC	MBS1	GC	METTL15	GC
ANXA5	GC	MLN	GC	APBA3	GC
AIP	GC	ITGAE	GC	ORC5	GC
TGFBR1	GC	DIO3	GC	DAZL	GC
GK	GC	BDKRB2	GC	ECE2	GC
TLR5	GC	GAK	GC	MRGPRX2	GC
PODXL	GC	UBE2E3	GC	TVP23B	GC
CACNA1G	GC	NR1H3	GC	INCA1	GC
GATM	GC	KIF14	GC	COL23A1	GC
ABCB1	GC	CDK4	GC	RASGEF1B	GC
DCX	GC	MSH3	GC	GPC5	GC
ADRB3	GC	PLA2G1B	GC	UBXN1	GC
TNIK	GC	SIAH1	GC	CDH20	GC
AGRP	GC	TERF1	GC	ENSG00000224836	GC
STX1B	GC	KRT7	GC	CFAP54	GC
CC2D1A	GC	CENPB	GC	COX11	GC
SLC52A3	GC	MIP	GC	CD3E	GC
FLNC	GC	PKD1L1	GC	ISG20	GC
MIR125A	GC	MIR27B	GC	PCAT1	GC
NRTN	GC	HPR	GC	AOAH	GC
LBR	GC	GABRR1	GC	LIPF	GC
SLC29A3	GC	CITED2	GC	WDR37	GC
DSP	GC	GIGYF1	GC	LINC02210-CRHR1	GC
STX1A	GC	MMP7	GC	CSAD	GC
ALDH18A1	GC	FKBP1A	GC	CSKMT	GC
MIR223	GC	FHIT	GC	HYAL3	GC
NDST1	GC	CD1A	GC	RNASE1	GC
PABPN1	GC	PMP2	GC	OLFM2	GC
LIMK1	GC	CASC16	GC	PRC1	GC
NOD2	GC	AZF1	GC	SULF1	GC
GHRH	GC	GH-LCR	GC	DCDC2C	GC
HK1	GC	CBLN1	GC	RPS26P52	GC
SLC9A9	GC	HM13	GC	ENSG00000237742	GC
GALC	GC	PCOS1	GC	LINC02238	GC

PPP2CA	GC	GRD2	GC	ENSG00000233005	GC
SLC6A9	GC	ROBO4	GC	ENSG00000286973	GC
NTS	GC	NARF	GC	MBOAT2	GC
ADRA2A	GC	MBD1	GC	FOXB1	GC
SLC6A19	GC	BRD1	GC	MTMR6	GC
SAG	GC	MSMO1	GC	CXDUPQ26.3	GC
PGAP1	GC	SLC4A4	GC	MYO1B	GC
P4HTM	GC	ST7	GC	PHACTR2	GC
CDH1	GC	PLEKHM2	GC	SQOR	GC
ABCC9	GC	TAF1L	GC	TSBP1	GC
IL18	GC	SYT12	GC	EIF3A	GC
MIR155	GC	AMBP	GC	GPAM	GC
ADA	GC	NCKAP1	GC	PTGER3	GC
MKRN3-AS1	GC	PTGDR	GC	PABPC1L	GC
PDGFRA	GC	SUMO2	GC	TRUB2	GC
ATL1	GC	ATOH1	GC	SNAPIN	GC
KIF5A	GC	F2RL1	GC	LRTM1	GC
TNNI3	GC	NEURL4	GC	SHISA6	GC
SETD2	GC	PPY	GC	HYAL2	GC
LHCGR	GC	HMOX2	GC	RNY5	GC
GPR101	GC	DUSP1	GC	DGKB	GC
RNU4ATAC	GC	DNTT	GC	AEBP2	GC
IKBKG	GC	CAV2	GC	HS3ST4	GC
KDM4C	GC	IFNL3	GC	POU2F1	GC
CYP27B1	GC	GNGT2	GC	PHTF1	GC
TDO2	GC	FOXD4	GC	KLHDC10	GC
ALPL	GC	GSK3A	GC	INTS5	GC
PICK1	GC	CGB7	GC	ZSCAN31	GC
LOC108684022	GC	NNMT	GC	NKAIN2	GC
ADCY5	GC	UBE2K	GC	CUL9	GC
PWAR1	GC	TAAR6	GC	BET1	GC
HP	GC	EHD4	GC	NXF1	GC
SHOC2	GC	ATP1A4	GC	TOMM22	GC
RBP4	GC	QRSL1	GC	SEMA3F-AS1	GC
ACTA2	GC	PLAUR	GC	TMED9	GC
RBM12	GC	RECQL5	GC	TTLL7	GC
APTX	GC	DIO2	GC	CDNF	GC
CD19	GC	SSTR2	GC	AOPEP	GC
MYBPC3	GC	PRDX5	GC	KIF6	GC
DUOX2	GC	HSPBP1	GC	LOC111365141	GC
EZH2	GC	EVX1	GC	TRIM24	GC

RTN4R	GC	ADAM22	GC	SLC27A6	GC
CISD2	GC	DUPXQ25	GC	TNFSF14	GC
MLXIPL	GC	NGEF	GC	C1QTNF4	GC
B3GALNT2	GC	CDH9	GC	ZBED9	GC
RPL10	GC	ANPEP	GC	HLA-L	GC
DCDC2	GC	APAF1	GC	KDM4A-AS1	GC
GRM7	GC	INSIG2	GC	HCG21	GC
PIGQ	GC	PPP3CC	GC	LINC01876	GC
MIR146A	GC	DCTN2	GC	MIR5579	GC
EZR	GC	PDC	GC	LINC01985	GC
SLC40A1	GC	MTDH	GC	RPL21P17	GC
NEU1	GC	GABRQ	GC	ENSG00000253844	GC
SLC45A1	GC	MFHAS1	GC	ENSG00000284989	GC
CEP152	GC	SH3KBP1	GC	ENSG00000259223	GC
ATAD1	GC	ANKRD31	GC	RN7SKP120	GC
CHRM2	GC	IGBP1	GC	ENSG00000270896	GC
MT-ND2	GC	LZTS1	GC	lnc-DDR1-4	GC
NR1H4	GC	POTEF	GC	ENSG00000212168	GC
F3	GC	LINC01546	GC	RF00017-5404	GC
PLEC	GC	RNU1-1	GC	ENSG00000287318	GC
MT-TF	GC	PTPRD-AS2	GC	ANKRD12	GC
WARS2	GC	PRKX-AS1	GC	ARL15	GC
TGM1	GC	CD2	GC	PALM2AKAP2	GC
SMAD3	GC	ABO	GC	SCGN	GC
LMOD3	GC	UBE3C	GC	FOXL1	GC
MITF	GC	TSNAX	GC	YTHDC1	GC
NRCLP3	GC	RAB29	GC	IL20RB	GC
ACADVL	GC	PTTG1	GC	PGLS	GC
IMPA1	GC	WASHC3	GC	PMEPA1	GC
RTTN	GC	GSTO2	GC	ARMC1	GC
TMEM106B	GC	CCR4	GC	TOMM34	GC
ZMPSTE24	GC	PRPF40A	GC	ZSCAN12	GC
HEPACAM	GC	RP1L1	GC	RALYL	GC
PQBP1	GC	EIF2AK2	GC	EPSTI1	GC
COL7A1	GC	C4B_2	GC	SGSM1	GC
SDHA	GC	TNFSF13	GC	SPOCK3	GC
GTF2IRD1	GC	CMA1	GC	SH3GL2	GC
SZT2	GC	ANKRD49	GC	ITGBL1	GC

KIF1B	GC	PCDHA3	GC	GTF2A1	GC
MIR144	GC	PRKDC	GC	AKAP1	GC
DSPS	GC OMIM	CHRNB3	GC	ERAS	GC
SMN1	GC	MIR98	GC	RNPEP	GC
PMPCA	GC	TXNDC9	GC	MTCH2	GC
NEUROD2	GC	EFHC2	GC	ZKSCAN4	GC
BGLAP	GC	HARS1	GC	ZSCAN9	GC
MIR106B	GC	HMGB1	GC	NOX5	GC
MTM1	GC	FZD3	GC	PSME4	GC
PACS1	GC	TEKT3	GC	SLC9A2	GC
BAZ1B	GC	PVR	GC	GRK6	GC
MIF	GC	UBE2E2	GC	FBXL7	GC
KCNJ5	GC	ASIC1	GC	SLC9A3R2	GC
PNPLA6	GC	GPX3	GC	MAPKAP1	GC
ALOX5	GC	S100A9	GC	PPP1R13L	GC
CXCR4	GC	SOGA3	GC	LINC01500	GC
CYP1A2	GC	OTUD7A	GC	ADAM24P	GC
CFH	GC	PTPRJ	GC	SLC22A23	GC
CIT	GC	MIR497	GC	OCEL1	GC
ANO3	GC	HHC2:066650	GC	TRAM2	GC
NPAP1	GC	HCNR606	GC	CCDC13	GC
COL5A2	GC	HCNR617	GC	EPHA5	GC
KCNJ8	GC	MAP3K11	GC	MAGI3	GC
MAPK10	GC	HTR1F	GC	TCF21	GC
HJV	GC	CTCFL	GC	GTF2E1	GC
ITGAM	GC	HBE1	GC	SUCLG2	GC
CSF3R	GC	MIR219A2	GC	FAM189A1	GC
TOP3A	GC	MAS1L	GC	DDX52	GC
BCHE	GC	RICTOR	GC	SEPTIN7P2	GC
VLDLR	GC	SNX2	GC	EEF1A1P14	GC
NRCLP2	GC	TTBK1	GC	NAT1	GC
OPTN	GC	AQP3	GC	CDK5RAP1	GC
ACAN	GC	DDI1	GC	NBPF22P	GC
HEXA	GC	CSH1	GC	LRRC1	GC
IL1A	GC	IL6ST	GC	PLD2	GC
PRKRA	GC	GPR55	GC	LGALS2	GC
MYT1L	GC	SNX6	GC	ST13P1	GC
HSPA1A	GC	SNX5	GC	DLG5	GC
SKI	GC	KCNIP1	GC	SCAND1	GC
CPLX1	GC	SP2	GC	ADGRB3	GC
IRF5	GC	RCC1L	GC	SART1	GC
TRPA1	GC	ANGPT1	GC	TMOD1	GC
HNRNPA1	GC	HAR1A	GC	CRACR2B	GC

UBQLN2	GC	AQP5	GC	LOC284395	GC
FBLN5	GC	EPM2AIP1	GC	TSPAN18	GC
PRPH2	GC	CHST12	GC	IP6K1	GC
BCR	GC	GTSCR1	GC	MED8	GC
HTR1D	GC	GLIS1	GC	C2orf69	GC
LINS1	GC	GLRA3	GC	LINC01135	GC
TBL2	GC	TNKS	GC	PTGIR	GC
GBE1	GC	SLC30A4	GC	WDR59	GC
EMD	GC	LAMA5	GC	WDFY1	GC
WAC	GC	NIPAL1	GC	LRCH3	GC
DLG4	GC	DEXI	GC	GFRA4	GC
NRCLP4	GC	SNORD112	GC	RBBP6	GC
NRCLP5	GC	ENSG00000271486	GC	EEF1G	GC
NRCLP6	GC	TBC1D5	GC	ELMOD1	GC
GCK	GC	TBCD	GC	NAA80	GC
GAPDH	GC	IDE	GC	CDRT7	GC
PRPH	GC	CXCR2	GC	OBI1-AS1	GC
GRID2	GC	CDCP2	GC	PAXIP1	GC
ABCA7	GC	EGR3	GC	PREX1	GC
CNGB3	GC	H4C1	GC	MAEA	GC
MT-TH	GC	OLIG3	GC	ADAMTS20	GC
IGHMBP2	GC	HAR1B	GC	BAG1	GC
CCR6	GC	HPS1	GC	HYI	GC
FSHR	GC	MIR382	GC	RIC8B	GC
BICD2	GC	TOM1L2	GC	IFITM4P	GC
APOL1	GC	MST1R	GC	DENND3	GC
SCARB2	GC	ID1	GC	GNB2	GC
ADAR	GC	MAP3K13	GC	HECW1	GC
CASP3	GC	PCA3	GC	GGT7	GC
MBP	GC	TUT1	GC	FHOD3	GC
RFC2	GC	SULT1A3	GC	FSIP1	GC
IGF2R	GC	MIR379	GC	FNBP4	GC
ARVCF	GC	CD163	GC	IPMK	GC
DLK1	GC	SMAD1	GC	CCDC69	GC
MED23	GC	RHEB	GC	SLIT1	GC
CTNNA3	GC	MAP11	GC	FICD	GC
GP6	GC	SMARCA5	GC	KIRREL1	GC
TBXA2R	GC	NSMF	GC	PEAK1	GC
ATPAF2	GC	CXCL11	GC	RASGEF1A	GC
KCNA4	GC	GRAP2	GC	TLX1	GC
IL12A	GC	MBNL2	GC	DEFA1	GC
PC	GC	SLC11A1	GC	DHX57	GC
AVPR2	GC	ARHGAP39	GC	MIA3	GC

TRIB2	GC	TGFA	GC	ORC3	GC
ACP5	GC	SRSF1	GC	JDP2	GC
TREM2	GC	ANKRD50	GC	ANKS1A	GC
GLUD2	GC	DGKQ	GC	USP34	GC
MEFV	GC	MFAP4	GC	S100A2	GC
TBK1	GC	MED9	GC	CTDSP2	GC
SEPSECS	GC	GID4	GC	NPNT	GC
MET	GC	LRRTM3	GC	UBOX5	GC
ADGRG1	GC	CHKA	GC	SRSF12	GC
CRPPA	GC	ADD1	GC	LINC01556	GC
PSMB8	GC	DSC3	GC	STK4-AS1	GC
TFR2	GC	MAP6	GC	LINC00240	GC
SIM1	GC	CCL7	GC	VAV3-AS1	GC
NIPAL4	GC	MFF	GC	SLC9A9-AS1	GC
CRBN	GC	HIP1	GC	MIR4301	GC
HLCS	GC	CPB2	GC	LINC01581	GC
ASAH1	GC	BRD2	GC	MICE	GC
GLDC	GC	DBX1	GC	GRM7-AS2	GC
SERPINF2	GC	NFIL3	GC	LINC01776	GC
WWOX	GC	AMD1	GC	RPL23AP1	GC
JMJD1C	GC	HR	GC	GNPATP	GC
DARS2	GC	DSTYK	GC	ZNF603P	GC
DDOST	GC	TRIM27	GC	LOC101928475	GC
PRDM10	GC	CHRM5	GC	ENSG00000229533	GC
DCN	GC	PTPRG	GC	ENSG00000124593	GC
TRDN	GC	RIBC1	GC	ENSG00000253238	GC
PRF1	GC	SRM	GC	ENSG00000248973	GC
ITGA2	GC	SH3GL1	GC	ENSG00000254035	GC
KAT6A	GC	GPR4	GC	KRT8P18	GC
DAG1	GC	CASC15	GC	ENSG00000261758	GC
ZBTB20	GC	MIR137HG	GC	LINC02450	GC
CACNG2	GC	CDC20	GC	RPS7P8	GC
NDUFV1	GC	SLC22A3	GC	ENSG00000243276	GC
POLR3A	GC	STAT5B	GC	FXNP1	GC
TUBB3	GC	GREM1	GC	RNA5SP181	GC
HADHB	GC	SNORD107	GC	EBLN3P	GC

HBA2	GC	DERL1	GC	ENSG00000223774	GC
PTHLH	GC	MED13	GC	ENSG00000223838	GC
ERCC8	GC	ID4	GC	ACTBP13	GC
MCTP2	GC	MCEE	GC	ENSG00000248538	GC
CHRNA1	GC	FBXW7	GC	ENSG00000249429	GC
CACNA1F	GC	POC1B	GC	PTP4A1P4	GC
CHD2	GC	GPI	GC	ENSG00000253901	GC
GSR	GC	GPR78	GC	ENSG00000266460	GC
C4B	GC	ZBTB33	GC	ENSG00000286608	GC
ALMS1	GC	ABCA13	GC	piR-39858-250	GC
ALDH3A2	GC	EIF4A1	GC	LOC105377632	GC
C12orf65	GC	LEF1	GC	ENSG00000261310	GC
TXNRD2	GC	PAWR	GC	ENSG00000227766	GC
SIGMAR1	GC	MEF2D	GC	ENSG00000236837	GC
SMN2	GC	ATP5PD	GC	lnc-VRK2-10	GC
PPP2R2B	GC	CD58	GC	lnc-FEN1-6	GC
CLIP1	GC	UHMK1	GC	ENSG00000287027	GC
PRTN3	GC	LYRM1	GC	lnc-RPGRIP1L-2	GC
LIAS	GC	MIR182	GC	ENSG00000280040	GC
COG5	GC	NTSR1	GC	piR-48749-002	GC
TBL1XR1	GC	YBX1	GC	RF00017-5528	GC
CSMD1	GC	PCM1	GC	ENSG00000271272	GC
HSPB8	GC	FGF13	GC	ENSG00000287634	GC
GLE1	GC	ULK2	GC	ENSG00000285409	GC
PTH1R	GC TTD	MYO16	GC	LOC105377628	GC
PDCD1	GC	TTI2	GC	ENSG00000254780	GC
NFIX	GC	PROCR	GC	ENSG00000225842	GC

SLC25A15	GC	RAMP1	GC	ENSG00000270541	GC
MCPH1	GC	MIR4422HG	GC	ENSG00000275827	GC
CSF3	GC	TMEM126A	GC	OLFM1	GC
KIF11	GC	CD9	GC	SCAI	GC
LARS2	GC	MTRNR2L5	GC	SCGB1D4	GC
MAP2	GC	NR2E1	GC	CTDSPL	GC
STUB1	GC	WDR11	GC	IPO9	GC
ABCB11	GC	MAP3K9	GC	MRPS24	GC
NFIB	GC	POF1B	GC	NR2F6	GC
VIP	GC	CHP1	GC	SLC38A3	GC
RAB11A	GC	SERPINE2	GC	MPZL3	GC
EYA1	GC	CERKL	GC	SLC22A11	GC
MT-CO3	GC	SNX1	GC	PYROXD2	GC
C1QBP	GC	KDM4A	GC	KBTBD11	GC
FDFT1	GC	MAP2K3	GC	EIF4G3	GC
ALAS2	GC	CLN9	GC	MDFIC	GC
FAAH	GC	ECT	GC	UBXN11	GC
GNPTAB	GC	DEL16P13.3	GC	TRIM69	GC
CLCN7	GC	S100A8	GC	ZBTB46	GC
PTGS2	GC	SP140	GC	CCN4	GC
MIR15A	GC	SYNGR1	GC	HDAC7	GC
FGF14	GC	MIR381	GC	SESN1	GC
NRXN3	GC	TNFRSF18	GC	RIPOR2	GC
KRT14	GC	ORC1	GC	KLHL1	GC
SCN3B	GC	ERC1	GC	C1orf87	GC
KMT2C	GC	FMO5	GC	ALOX15B	GC
HNRNPA2B1	GC	CXCR1	GC	MMS19	GC
TECPR2	GC	PGAM5	GC	KCTD15	GC
CAPN1	GC	RFX2	GC	RADIL	GC
AGL	GC	CASZ1	GC	ZNF460	GC
CD4	GC	MAGI2	GC	LRRC18	GC
ROBO3	GC	SPN	GC	PIP5K1B	GC
SLC4A1	GC	P2RX2	GC	VPS35L	GC
TACR3	GC	SLC30A6	GC	ZC3H11A	GC
OPA3	GC	RARRES2	GC	CLTB	GC
MED25	GC	GRIN3B	GC	OTUD7B	GC
CALM1	GC	C8orf37	GC	SLC39A6	GC
STT3A	GC	VILL	GC	TMPRSS11D	GC
TCAP	GC	KRT18	GC	HIGD1A	GC
FLG	GC	VPS29	GC	RAB17	GC
HTR7	GC TTD	ZNF746	GC	CDHR3	GC
NEK1	GC	COBL	GC	TGFBRAP1	GC

ABCD1	GC	KCNN1	GC	FAM218A	GC
HIBCH	GC	CD1D	GC	KLK9	GC
NOS1AP	GC	PEG10	GC	MBOAT1	GC
WDR4	GC	ARR3	GC	CYB561D1	GC
TRH	GC	CTPS1	GC	FARP1	GC
HBA1	GC	KCNJ16	GC	ACSL1	GC
NTF4	GC	RAN	GC	ILF3	GC
COMP	GC	CCT4	GC	F11R	GC
MATR3	GC	MAFB	GC	MAGEC2	GC
CYP11B1	GC	PAM	GC	SLC9A4	GC
FGF12	GC	CRYZL1	GC	ZNF641	GC
SLC35A3	GC	PCSK1N	GC	ATF7IP2	GC
CHCHD10	GC	RSS	GC	AMIGO3	GC
KCND3	GC	TRAPPC2B	GC	SMAD5-AS1	GC
PRSS12	GC	ID3	GC	LOC105372310	GC
PKD1	GC	SMCHD1	GC	TM9SF2	GC
APOH	GC	ATP13A3	GC	UMODL1	GC
CYP2C19	GC	SLC47A1	GC	GLCCI1	GC
ETFDH	GC	SMCR2	GC	TNIP2	GC
SCN2B	GC	SMCR6	GC	EIF5A	GC
GMPPB	GC	HSD17B13	GC	PRKCQ	GC
RORB	GC	TSNAX-DISC1	GC	DISP3	GC
HAND2	GC	DIP2A	GC	C1GALT1	GC
IYD	GC	GABARAPL1	GC	AP1B1	GC
EEF1A2	GC	KLK4	GC	MARF1	GC
APOC3	GC	IL1R2	GC	PRELID1	GC
ATP1A1	GC	HAVCR1	GC	DEFB128	GC
MSN	GC	SCLY	GC	TMEM158	GC
ASMT	GC	GRP	GC	LTBR	GC
AKAP9	GC	SRSF6	GC	E2F3	GC
TUBB2B	GC	CD274	GC	SETMAR	GC
SMARCA4	GC	MIR1185-1	GC	GRXCR1	GC
TCF7L2	GC	WHAMM	GC	RBM39	GC
HSD11B1	GC	LRRC41	GC	CD93	GC
GATAD2B	GC	SRP68P1	GC	ITM2C	GC
GRIA2	GC	MIR346	GC	LEMD2	GC
SLC22A5	GC	SGPL1	GC	HMBOX1	GC
CUL3	GC	RHBDF2	GC	HNRNPD	GC
GJA5	GC	LINC01734	GC	LUC7L3	GC
TRAPPC6B	GC	CRELD1	GC	DEDD	GC
SH3BP2	GC	CHN2	GC	OLFM4	GC
ADAMTS13	GC	SSTR4	GC	SYT17	GC
EML1	GC	AJAP1	GC	DIRC1	GC
DYM	GC	LCA5	GC	GNPDA1	GC

MEIS1	GC	QKI	GC	MYO3B	GC
TSEN54	GC	GPRIN1	GC	ENY2	GC
ALDH2	GC	SCOC-AS1	GC	JHY	GC
TRHR	GC	S100A6	GC	GDF7	GC
NOP56	GC	ENTR1	GC	PLCXD2	GC
UROD	GC	ITPR3	GC	GALNT13	GC
MIR483	GC	GCFC2	GC	SENP5	GC
ZNF81	GC	MIR675	GC	KRCC1	GC
MMP13	GC	PLD1	GC	PBX4	GC
IHH	GC	GPC6	GC	TAS2R14	GC
CRADD	GC	VPS54	GC	ARHGAP28	GC
HMGCR	GC	ADRA1B	GC	MXD3	GC
EPAS1	GC	CCDC144A	GC	LRP3	GC
PRDM16	GC	SOX11	GC	MPHOSPH6	GC
KCNK3	GC	EIF4A2	GC	CHST2	GC
MYO7A	GC	USP24	GC	PDIA3	GC
RTN4	GC	SYT11	GC	TMEM30B	GC
CCL5	GC	RANBP17	GC	GPAT3	GC
LIPN	GC	RNF11	GC	LINC00841	GC
ACTG1	GC	AURKA	GC	CCRL2	GC
NFE2L2	GC	AADAC	GC	NAP1L1	GC
GHSR	GC	H2BC21	GC	SNORA13	GC
CSNK2A1	GC	MS4A2	GC	RGS14	GC
MAPK1	GC	STRADB	GC	LAYN	GC
TRAK1	GC	LMX1A	GC	CCDC27	GC
TGFB3	GC	CCDC8	GC	DEUP1	GC
DUOXA2	GC	MIR20B	GC	ZNF217	GC
BTK	GC	USP40	GC	ARHGEF10L	GC
SLC1A4	GC	VPS26B	GC	DDX17	GC
PPARGC1A	GC	VAPA	GC	DDN	GC
MYLK	GC	LNPEP	GC	HTATIP2	GC
GNRHR	GC	APBB1	GC	STAP2	GC
MRAP	GC	LOC106050103	GC	GPR156	GC
DNMT3A	GC	RPSA	GC	FGFBP1	GC
SNX14	GC	NT5E	GC	CDK5R2	GC
PPARG	GC	GAS5	GC	SEMA3B	GC
RECQL4	GC	AMT	GC	BTBD3	GC
TPM2	GC	HOMER3	GC	CREB3L2	GC
SIL1	GC	USP2	GC	SUSD1	GC
DDX41	GC	LOC108663985	GC	LIFR-AS1	GC
INPP5E	GC	PRKG2	GC	PCF11	GC
ASXL3	GC	STK32B	GC	GPR141	GC
ADCY10	GC	PIGK	GC	GAB1	GC
CYP3A4	GC	GOLGA2	GC	ZMYM4	GC

TNPO3	GC	MST1	GC	MX2	GC
DICER1	GC	SKP1	GC	PLD5	GC
FGF2	GC	CDC34	GC	GREB1	GC
CILK1	GC	PSMA5	GC	SAMD4A	GC
CIZ1	GC	RPN2	GC	HACD2	GC
CBS	GC	CAVIN2	GC	RAB3C	GC
GNAI3	GC	CDKAL1	GC	SEMA3F	GC
SCNN1A	GC	GPR89B	GC	PFN3	GC
AGPAT2	GC	GPR89A	GC	IPO5	GC
AGTR2	GC	TRPM3	GC	CNBD1	GC
VAPB	GC	ATXN3L	GC	PCDHGA3	GC
SOD2	GC	REM1	GC	SMYD1	GC
RLS6	GC	CCDC62	GC	RLN3	GC
LITAF	GC	DPY19L2P2	GC	ACKR4	GC
MCCC1	GC	UCHL1-AS1	GC	SNRNP48	GC
ANKK1	GC	LINC01262	GC	CD53	GC
GHRHR	GC	UCH1LAS	GC	NMNAT2	GC
ENO2	GC	TRIP12	GC	GPR83	GC
SIK1	GC	KCNK2	GC	CAND1	GC
CYP2B6	GC	TTC8	GC	RNF123	GC
STT3B	GC	RNF13	GC	PBX3	GC
NLGN1	GC	PPP1R9B	GC	ASCL4	GC
MDD2	GC OMIM	MIR128-1	GC	PABPC1P2	GC
PEX14	GC	TRAF3IP2-AS1	GC	BNIP2	GC
CA2	GC	ADH1A	GC	SRP14	GC
SLC16A2	GC	FERD3L	GC	MPP7	GC
HCN4	GC	PAF1	GC	SMARCD1	GC
ENG	GC	GSTT1	GC	NIPSNAP3B	GC
GM2A	GC	BST1	GC	BUB1B-PAK6	GC
NADK2	GC	TBXAS1	GC	UBL5	GC
KCNJ1	GC	EIF4EBP2	GC	MRPL1	GC
NPTX2	GC	CRAT	GC	GSTM4	GC
KCNC1	GC	GLRA4	GC	PHLPP1	GC
ANOS1	GC	SWI5	GC	LOC101928516	GC
OPRK1	GC	STMN2	GC	RALBP1	GC
CD59	GC	HGF	GC	INSC	GC
PPP3CA	GC	PNLIP	GC	GTF2B	GC
HNRNPH2	GC	BACH2	GC	ZNF536	GC
WAS	GC	RNF170	GC	PTENP1	GC
EBP	GC	MIR92A1	GC	ATP6V1C2	GC
GLI2	GC	NSUN5	GC	GPA33	GC
EPOR	GC	ATP13A1	GC	NSL1	GC
PCGF2	GC	CDH17	GC	CMC2	GC

MDD1	GC OMIM	ADCY2	GC	TMEM150C	GC
ARSH	GC	ESS2	GC	LINC00996	GC
CHCHD2	GC	HLA-DRB3	GC	RBM6	GC
PMS2	GC	SCG5	GC	EVA1A	GC
SELE	GC	PAPOLG	GC	STYX	GC
MC2R	GC	BRD4	GC	CIAO2B	GC
MIR9-1	GC	IQCK	GC	DMC1	GC
GNAS-AS1	GC	TBC1D20	GC	CASP8AP2	GC
SLC33A1	GC	DNAJB2	GC	TMEM109	GC
ALG14	GC	PPT2	GC	SIGLEC1	GC
SOS1	GC	H4C11	GC	KDELR3	GC
DYNC1I2	GC	CD209	GC	UBN1	GC
HIVEP2	GC	DEFB1	GC	HELT	GC
SPTLC1	GC	CHD5	GC	ZNF500	GC
PIGP	GC	KANTR	GC	IPO9-AS1	GC
CAPN3	GC	PLIN1	GC	RGS12	GC
MOCS1	GC	CNIH2	GC	CSE1L	GC
PGAP3	GC	SAMD11	GC	MS4A3	GC
LINGO1	GC	BHLHE40	GC	SLC28A3	GC
SLC12A1	GC	RBCK1	GC	PKHD1L1	GC
PICALM	GC	GNAT1	GC	PHTF2	GC
F13A1	GC	SCG2	GC	ZPBP2	GC
MMP1	GC	UBE4A	GC	OR51F1	GC
SNHG14	GC	GCLM	GC	GLYR1	GC
UMOD	GC	KCTD3	GC	TMEM182	GC
AIMP1	GC	GDF15	GC	CDC37	GC
ASH1L	GC	FUT2	GC	AADACL4	GC
TPK1	GC	SV2C	GC	PPP6R1	GC
GRIK1	GC	LRRC7	GC	ENOPH1	GC
AIMP2	GC	PRR12	GC	CDC16	GC
ELP1	GC	LY86	GC	RFC3	GC
ERBB3	GC	CTTN	GC	RSBN1	GC
TPI1	GC	CUL7	GC	LRRC59	GC
SERPINA6	GC	COX4I1	GC	CAMKV	GC
AGA	GC	SRSF9	GC	VGLL2	GC
HLA-G	GC	TET3	GC	CGRRF1	GC
CR1	GC	AGO1	GC	PTPN13	GC
AMH	GC	ASIC3	GC	HNRNPA0	GC
CTSF	GC	UBE2V1	GC	RTRAF	GC
CALM2	GC	TFE3	GC	JOSD1	GC
SARS1	GC	CHD6	GC	HOXD3	GC
IFIH1	GC	FNDC3A	GC	ARHGAP18	GC
SULT2B1	GC	GPR153	GC	KLHL5	GC

TRPS1	GC	UBE2G2	GC	SF3A3	GC
MIR34C	GC	MBD2	GC	CPEB4	GC
PDHA1	GC	LAMB3	GC	BCAS3	GC
CYP27A1	GC	GGCT	GC	MOV10L1	GC
ADAM17	GC	GC	GC	GPRC5B	GC
COL11A2	GC	SIX6	GC	RFFL	GC
NSD2	GC	WIPI1	GC	STARD4-AS1	GC
TRPV3	GC	DENND11	GC	TAFA2	GC
CCDC47	GC	CALCB	GC	CHKB-CPT1B	GC
TACO1	GC	MIR193B	GC	EPS8	GC
MDH2	GC	NUP160	GC	PCDH18	GC
STK11	GC	TSPOAP1	GC	GPSM3	GC
SEPTIN9	GC	FRAS1	GC	CHCHD3	GC
MIR148B	GC	TIRAP	GC	TACC2	GC
SEMA5A	GC	TSPAN2	GC	EEPD1	GC
PEPD	GC	LIPJ	GC	CFAP299	GC
NPSR1	GC	HNRNPF	GC	EEF1A1P22	GC
GPC3	GC	TCF4-AS1	GC	TSKU	GC
ATAD3A	GC	RPTOR	GC	OXNAD1	GC
ADRA1A	GC	SEZ6L	GC	SPAG7	GC
CYFIP2	GC	STX17	GC	RGS16	GC
HTR3B	GC	LRRN1	GC	PSME3IP1	GC
TUBA1A	GC	LINC00271	GC	H3-5	GC
MAPK14	GC	H4C12	GC	C11orf97	GC
RNASEL	GC	TPTE	GC	MTA2	GC
KCNA5	GC	ISG15	GC	IDO2	GC
OCA2	GC	INHA	GC	PLCB2	GC
TRIO	GC	KCNK6	GC	PARP4	GC
MYCN	GC	CCR2	GC	TANK	GC
COL17A1	GC	VAV3	GC	EHF	GC
ARCN1	GC	DUP5P13	GC	OR6C1	GC
MAD1L1	GC	DEL17P13.1	GC	BCAP29	GC
IBA57	GC	BFIS1	GC	OR10Z1	GC
MAGT1	GC	PRSS8	GC	MIR3976HG	GC
WDR62	GC	LRRN3	GC	SP140L	GC
MYD88	GC	CHD4	GC	PSD4	GC
HAPLN1	GC	TNXA	GC	PRPF4B	GC
RAC2	GC	MOV10	GC	ARID4B	GC
KCNK9	GC	MIRLET7B	GC	RNF169	GC
WDR73	GC	POU4F1	GC	CTPS2	GC
HSD3B2	GC	GPHA2	GC	CLASP1	GC
IL7R	GC	CCL20	GC	GDPD4	GC
JUP	GC	THAP3	GC	RAD1	GC
POMT1	GC	PDPK1	GC	CEP72	GC

OPRD1	GC	POLE	GC	ADGRB2	GC
SIRT1	GC	FGF18	GC	DDX60L	GC
PDE10A	GC	HABP2	GC	FHDC1	GC
CTSK	GC	CD1C	GC	RIDA	GC
BAP1	GC	SPECC1L	GC	IGSF11	GC
FBP1	GC	EXOC1	GC	ATAD2B	GC
ABCB4	GC	GRHPR	GC	ARPC2	GC
NT5C2	GC	LOC114803470	GC	CPSF2	GC
LRP4	GC	RAB11FIP5	GC	ZC3H4	GC
MEG3	GC	AGAP6	GC	NIBAN1	GC
TM4SF20	GC	SUCO	GC	PUM3	GC
PLOD1	GC	CCS	GC	ECT2	GC
TNFRSF10A	GC	NMUR2	GC	RSBN1L	GC
CUX2	GC	MDGA2	GC	GPR176	GC
PPOX	GC	RNF41	GC	EMC7	GC
SLC12A6	GC	H4C9	GC	FAM83B	GC
CYP11B2	GC	ETS1	GC	RPRD2	GC
SPIB	GC	ANKS1B	GC	RFC5	GC
WDR45B	GC	H4C3	GC	NEK6	GC
COG4	GC	H4C8	GC	USP6NL	GC
ASXL1	GC	H4C6	GC	SCRN1	GC
CNTF	GC	FCGR1A	GC	SFXN1	GC
ACTB	GC	KCNE4	GC	UPP2	GC
CRYAA	GC	JARID2	GC	LRFN2	GC
CPLANE1	GC	COCH	GC	INTS12	GC
PAX2	GC	DNMT3L	GC	C3orf67	GC
UNC13A	GC	H4C2	GC	OPN3	GC
MIR29A	GC	H4C13	GC	APOBEC3C	GC
RRAS	GC	H4C4	GC	UNC79	GC
PRICKLE1	GC	H4C5	GC	LMAN2	GC
SLC13A5	GC	CACNA1C-AS2	GC	GFPT2	GC
CUX1	GC	PPP1R3F	GC	STK17A	GC
SELL	GC	CSRP1	GC	BDP1	GC
NBAS	GC	IPPK	GC	RIMS4	GC
BLOC1S1	GC	TRAT1	GC	CACTIN	GC
COG1	GC	PFAS	GC	TMEM189-UBE2V1	GC
PLA2G4A	GC	PPP1R13B	GC	FLJ33534	GC
WNT4	GC	HPX	GC	LOC401478	GC
TNFRSF11A	GC	NCOA2	GC	BUD13	GC
AVPR1B	GC	SUPT5H	GC	USP28	GC
MMEL1	GC	PTGDR2	GC	EFNA1	GC
GPD1L	GC	HPD	GC	S100P	GC
PLPBP	GC	ESPL1	GC	SS18	GC

GYS1	GC	TPT1	GC	PLEKHG6	GC
NTNG1	GC	CERS1	GC	AFAP1L1	GC
LGI1	GC	SHOX2	GC	KDM8	GC
FSHB	GC	KLK7	GC	TRIM67	GC
TUBB4A	GC	POLD1	GC	UBTD1	GC
TMEM43	GC	NPRL3	GC	C2CD4C	GC
TBL1X	GC	EDEM1	GC	C14orf177	GC
A2M	GC	GOT2	GC	LINC00917	GC
RLS7	GC	TEKT5	GC	WDR11-AS1	GC
IFT43	GC	RNF217-AS1	GC	XRRA1	GC
CD79A	GC	KLKB1	GC	SNRK	GC
ITGB2	GC	GNB4	GC	RASAL2	GC
SNORD116	GC	ESX1	GC	LOC100129620	GC
EPCAM	GC	MIR652	GC	GALNTL6	GC
PI4KA	GC	CDC14A	GC	TAB1	GC
CRX	GC	FARP2	GC	DEFB127	GC
M6PR	GC	PIK3CD	GC	IZUMO1R	GC
CASQ2	GC	SNAI1	GC	ENC1	GC
CR2	GC	HBEGF	GC	VPS37C	GC
SIX3	GC	LTF	GC	GPR26	GC
NRGN	GC	PIRC2	GC	OR2J3	GC
NFKB2	GC	PIRC4	GC	TANGO6	GC
AMELX	GC	MMAB	GC	H2AC6	GC
PSENEN	GC	UPK1A	GC	RNF40	GC
GAP43	GC	THADA	GC	EPC1	GC
REST	GC	KIR3DL1	GC	C10orf90	GC
TRIP13	GC	CABIN1	GC	PROKR1	GC
HOXB1	GC	TSNARE1	GC	GBF1	GC
P3H1	GC	FZD9	GC	LRP1B	GC
NUP107	GC	NFKBIL1	GC	EPB41L4A	GC
INF2	GC	FMO2	GC	TENM2	GC
ARMC9	GC	XIST	GC	ZNF561	GC
CHRNG	GC	PPP1R15A	GC	SNTG1	GC
SLC5A5	GC	GJA3	GC	BCL2L14	GC
ATP12A	GC	NOP2	GC	NFE2L3	GC
UNC80	GC	LIF	GC	VGLL3	GC
DCPS	GC	NLRP5	GC	ATXN7L1	GC
HCFC1	GC	DNAH12	GC	LRIF1	GC
OCRL	GC	VDAC3	GC	CABCOCO1	GC
HTR2B	GC	USF1	GC	TPI1P2	GC
FGD1	GC	BCL2L1	GC	RPL6P25	GC
PLAT	GC	NFATC1	GC	EGID-100124696	GC
HCCS	GC	DEFB4A	GC	CCDC162P	GC
DSG2	GC	APLN	GC	GASK1A	GC

DBP	GC	TLE1	GC	STK32A	GC
APPL1	GC	NBPF3	GC	GIN1	GC
CPT1B	GC	NMT1	GC	RBM34	GC
CTNND2	GC	LDHA	GC	JAML	GC
CHM	GC	NKX6-2	GC	MIR4789	GC
MTRR	GC	SOBP	GC	RGS20	GC
STAT4	GC	SNRPE	GC	TAS2R1	GC
TCN2	GC	CD83	GC	ZBED5	GC
CALB1	GC	EIF4H	GC	FAM241B	GC
CXCL10	GC	MIR95	GC	RHOBTB3	GC
DLD	GC	ZNRD1ASP	GC	TMX2	GC
TSC22D3	GC	MIR432	GC	ABHD14B	GC
PGAP2	GC	TIMM13	GC	ZBED8	GC
SERPINI1	GC	FBLN2	GC	SH3RF1	GC
SMARCAL1	GC	PPP3R1	GC	RFTN1	GC
SMARCC2	GC	PDE4DIP	GC	TRPC7	GC
MAFD1	GC	FMO4	GC	ZNF691	GC
MIR29C	GC	COMA	GC	RMDN2	GC
RETREG1	GC	SNX13	GC	ZNF66	GC
LARP7	GC	NTM	GC	OR2BH1P	GC
NONO	GC	KIFAP3	GC	RAPGEF5	GC
HSPA9	GC	TRIM9	GC	TRIM11	GC
PYY	GC	NFIC	GC	PRLH	GC
MIR24-1	GC	NXPH1	GC	PDXP	GC
GPD1	GC	NMBR	GC	HLX	GC
IL3	GC	TXK	GC	MAGEA9B	GC
HSP90AA1	GC	ABCC3	GC	SLC2A12	GC
THRA	GC	DLAT	GC	C1RL	GC
UBA5	GC	PTCHD1-AS	GC	PCMTD1	GC
SLC37A4	GC	MIR369	GC	GPATCH2	GC
SLC10A2	GC	KCNK10	GC	ZNF562	GC
ATP4A	GC	RAI14	GC	MB21D2	GC
KMT2B	GC	ALDH1B1	GC	STEAP1B	GC
EMX2	GC	PRPS1L1	GC	ARMH4	GC
TMLHE	GC	MIR181D	GC	LINC01184	GC
COL10A1	GC	SYNPO	GC	LINC01722	GC
HDAC9	GC	DCDC1	GC	PA2G4P2	GC
MIR30E	GC	ICAM3	GC	USE1	GC
EIF2AK3	GC	BAZ2B	GC	PSD3	GC
LRSAM1	GC	C1QTNF3	GC	ACTR3B	GC
PKP2	GC	LALBA	GC	NCEH1	GC
GRM2	GC	PTBP1	GC	HMGCLL1	GC
BACE1	GC	ENSG00000230894	GC	HERPUD2	GC

SLC2A2	GC	DNAJC14	GC	FOXD2	GC
MAPK3	GC	TCF3	GC	TMEM45B	GC
KLHL40	GC	TRO	GC	NLRP8	GC
PF4	GC	YIF1A	GC	FAM180A	GC
POU2AF1	GC	SGCB	GC	PLEKHS1	GC
HINT1	GC	ESRRG	GC	TRABD2B	GC
CORIN	GC	ZCCHC12	GC	CFAP77	GC
KRT5	GC	SYNJ2	GC	UMAD1	GC
KDM6A	GC	ADH7	GC	NPM2	GC
PROS1	GC	EPS15	GC	TMEM178A	GC
SLC18A1	GC	CX3CL1	GC	C8orf34	GC
KNG1	GC	RIPK1	GC	CRYBG2	GC
PFKM	GC	BLZF1	GC	USP48	GC
IL17F	GC	LHFPL5	GC	BTF3	GC
APRT	GC	MAP3K7	GC	NCBP2	GC
GRIP1	GC	LHX8	GC	CLSTN3	GC
ABCC2	GC	ATG9A	GC	IFRD2	GC
CD8A	GC	PRPF40B	GC	EIF2S2P7	GC
DHCR24	GC	MEST	GC	ITGB7	GC
RIMS1	GC	TRIM2	GC	MAPK15	GC
ZFYVE27	GC	NIF3L1	GC	IFNW1	GC
CX3CR1	GC	H4C15	GC	MRPL48	GC
TRPM1	GC	GNA14	GC	SETD4	GC
PSAT1	GC	MLST8	GC	OR52K1	GC
TRAPPC2	GC	KCNB2	GC	MMP27	GC
LMOD1	GC	TSPY1	GC	LNX2	GC
TNFSF15	GC	PSIP1	GC	DEFA6	GC
LMAN2L	GC	H4C14	GC	TASP1	GC
NR2E3	GC	SACM1L	GC	CPNE2	GC
WNT7A	GC	HOOK1	GC	MTIF3	GC
TGM5	GC	ATG2A	GC	TSTD1	GC
LTBP2	GC	RAMP3	GC	ARRDC1	GC
TPM1	GC	F2R	GC	RANBP3L	GC
IFNA1	GC	IRF7	GC	C6orf201	GC
PLN	GC	CSK	GC	DBNDD2	GC
TIMP1	GC	ENAM	GC	LSMEM2	GC
SERPINH1	GC	AKR7A2	GC	PNMA8A	GC
ADM	GC	SERPINB2	GC	MIR1179	GC
RLS1	GC	VPS16	GC	RPS23P3	GC
RCBTB1	GC	FOXM1	GC	CYP27C1	GC
BSND	GC	DNAJC30	GC	RBM5	GC
SLC25A11	GC	TUG1	GC	DEFA4	GC
EFNB1	GC	PLIN3	GC	RAB24	GC
ALX3	GC	GABARAP	GC	HOXD4	GC

WIPI2	GC	ITPA	GC	FAM107A	GC
TK2	GC	GABARAPL2	GC	ARHGAP30	GC
NF2	GC	LMO2	GC	RNF167	GC
STIM1	GC	KNOP1	GC	PM20D2	GC
IL5	GC	GYPE	GC	TMEM26	GC
CCR3	GC	DPY19L1	GC	PIWIL3	GC
EXOSC3	GC	ME2	GC	ZNF90	GC
TTBK2	GC	EMX1	GC	ZNF454	GC
FHL1	GC	PCSK2	GC	OR7G1	GC
GYPB	GC	RIPPLY3	GC	FASTKD5	GC
HNF1A	GC	SNTB2	GC	ZNF486	GC
FOXE1	GC	ATP5PO	GC	ZNF626	GC
GRM3	GC	RILP	GC	ANKRD30B	GC
TNXB	GC	CD1E	GC	OR7G2	GC
LIPA	GC	MIR484	GC	PPP5D1	GC
MT-ND4	GC	CGB3	GC	RTL6	GC
PLA2G7	GC	RPLP1	GC	PNMA8B	GC
PREPL	GC	MYH10	GC	ZNF826P	GC
PHKA2	GC	LMLN	GC	LINC01619	GC
CDK20	GC	WDR20	GC	GUCY2EP	GC
MT-CO2	GC	C17orf107	GC	POM121L10P	GC
PGR	GC	MIR1304	GC	RPS2P6	GC
KCNE3	GC	CXCR5	GC	ECI2-DT	GC
COG8	GC	SYNM	GC	MIR4465	GC
KARS1	GC	DHRS9	GC	PNMA8C	GC
KCNV2	GC	LPP	GC	LOC728739	GC
HSD11B2	GC	CNIH3	GC	RPL30P3	GC
COL4A3	GC	RUNX1T1	GC	PAK6	GC
FMN2	GC	MED19	GC	FAAH2	GC
TYROBP	GC	KAAG1	GC	VWA5A	GC
RB1	GC	LOC110121269	GC	OR52B4	GC
TJP2	GC	TK1	GC	LOC101928387	GC
MAFD2	GC	IL1RL1	GC	USP32	GC
MAFD8	GC OMIM	KRT19	GC	AATK	GC
MAFD9	GC OMIM	MKI67	GC	CLDN23	GC
LIG4	GC	RAMP2	GC	SAMD4B	GC
MEGF10	GC	TOR1AIP1	GC	ACBD7	GC
PRKCD	GC	TMEM79	GC	KLHL23	GC
IL1R1	GC	NSUN6	GC	PRSS55	GC
GSTM1	GC	NPAS1	GC	ACTL8	GC
MIR210	GC	GAS8	GC	LINC00907	GC
PIGT	GC	CD46	GC	LOC284930	GC

CERT1	GC	ROGDI	GC	POLD2P1	GC
FGB	GC	RNU6ATAC	GC	ASS1P11	GC
ALAD	GC	STX8	GC	RPL31P35	GC
MBOAT7	GC	NBR1	GC	PARD6A	GC
PHF6	GC	SEL1L	GC	CTNNAL1	GC
DSC2	GC	OSM	GC	ASB13	GC
NPR2	GC	PUM1	GC	IFNA21	GC
MAFD3	GC	ADH6	GC	MPRIP	GC
MAFD4	GC	NOVA1	GC	CEP85	GC
MAFD5	GC OMIM	CSN3	GC	RABL3	GC
MAFD6	GC	AGO2	GC	TPGS2	GC
IDO1	GC	POLR2G	GC	DDIAS	GC
ATP6V1A	GC	CYP51A1	GC	RASSF10	GC
HMGCL	GC	PDIA4	GC	OR5T3	GC
LOC109504728	GC	SYNCRIP	GC	ODF3B	GC
HIVEP1	GC	FAS-AS1	GC	C19orf18	GC
CTSC	GC	SLC22A18	GC	FBXO47	GC
KDM6B	GC	KCNT2	GC	C5orf66	GC
BMPR2	GC	FGF4	GC	CSNK1G3	GC
GMPPA	GC	MCM5	GC	CCDC167	GC
EDC3	GC	ERI3	GC	LINC00893	GC
LAMA1	GC	KIAA1586	GC	IFN1@	GC
RPS3A	GC	FCER1A	GC	PPP1R21	GC
WARS1	GC	PDS5A	GC	STMN4	GC
ATP2A1	GC	KAT5	GC	IPO4	GC
AVPR1A	GC	KYAT1	GC	KCTD18	GC
YY1	GC	KAT2B	GC	CRYL1	GC
OAT	GC	AKR1B1	GC	RHPN2	GC
IMMP2L	GC	RTN4IP1	GC	OLFML3	GC
RPGRIP1L	GC	FADS2	GC	TUBD1	GC
RBM8A	GC	XIRP2	GC	TOM1L1	GC
MYPN	GC	AP2B1	GC	CCNG2	GC
MIR93	GC	EFTUD2	GC	ADGRL2	GC
VRK1	GC	TLDC2	GC	GPR6	GC
FKBP10	GC	RSU1	GC	SLC46A3	GC
G6PC3	GC	EEF1AKNMT	GC	RNFT1	GC
ZC3H14	GC	INSIG1	GC	UBXN2A	GC
CDK5RAP2	GC	LOC106029312	GC	SNX29	GC
TGFBI	GC	SHPK	GC	ADSS2	GC
CSF2	GC	MCHR2-AS1	GC	OR2S2	GC
METTL23	GC	S100A7	GC	OR52D1	GC
TRAF6	GC	CA10	GC	C2orf42	GC

RAB40AL	GC	MBNL3	GC	OR10T2	GC
DGCR2	GC	RBBP5	GC	SLC25A48	GC
SPTLC2	GC	TAPBPL	GC	OR10K1	GC
PCBD1	GC	PSPH	GC	OR52E6	GC
GRIK4	GC	YAP1	GC	MORN2	GC
ZFP57	GC	MAU2	GC	OR8S1	GC
USP7	GC	DGAT2	GC	EEF1AKMT2	GC
ABCG5	GC	DUOX1	GC	TPTE2P1	GC
PTK7	GC	NPL	GC	HBBP1	GC
RGS4	GC	CLEC4C	GC	KU-MEL-3	GC
KRT16	GC	PEE1	GC	LOC154449	GC
GYG1	GC	CWC22	GC	RPL21P119	GC
TECR	GC	ATP13A4	GC	TVP23CP2	GC
RARS2	GC	IGES	GC	LOC101927078	GC
DHODH	GC	CYB561D2	GC	AIMP1P1	GC
BBS9	GC	VASP	GC	HDAC10	GC
SARDH	GC	CENPI	GC	PIDD1	GC
MAPK8	GC	HIPK3	GC	UTS2B	GC
PCK1	GC	IST1	GC	PCDHB10	GC
SCT	GC	FCN2	GC	EOLA1	GC
CAPN10	GC	SH2D3A	GC	EOLA2	GC
CRLF1	GC	HSF1	GC	TRAJ10	GC
ALX4	GC	PRR5	GC	PTGER1	GC
GP1BB	GC	SOX2-OT	GC	KLHL20	GC
HRH1	GC	ESCO1	GC	APOL6	GC
RLS2	GC	SPTSSB	GC	CLDND1	GC
NPHS1	GC	AATF	GC	NASP	GC
COL6A1	GC	CEACAM5	GC	ZDHHC16	GC
KCNC3	GC	UCP3	GC	ATP5MC3	GC
ICOSLG	GC	CCL24	GC	LINC00894	GC
TCIRG1	GC	APLNR	GC	DNAJB1P1	GC
CDH13	GC	ABCC4	GC	LINC02112	GC
SP7	GC	CDC42SE2	GC	PEX10	OMIM
RARB	GC	DLG2-AS1	GC	NALD	OMIM
CLU	GC	PANK4	GC	PBD6A	OMIM
VRK2	GC	CARMIL2	GC	PBD6B	OMIM
CYCS	GC	XIRP1	GC	PER3	OMIM
PSMB9	GC	RAB6A	GC	FASPS3	OMIM
PFN1	GC	CNTNAP5	GC	SLC45A1	OMIM
RNF135	GC	UBA1	GC	DNB5	OMIM
VPS11	GC	SLC30A8	GC	IDDNPF	OMIM
MBTPS2	GC	FKBP1B	GC	RERE	OMIM
MSMB	GC	COQ7	GC	NEDBEH	OMIM
DSG1	GC	STOM	GC	PEX14	OMIM

SPINK5	GC	INS-IGF2	GC	PBD13A	OMIM
GFPT1	GC	RNF207	GC	DDOST	OMIM
FBXO31	GC	ARHGEF26	GC	OST	OMIM
CFAP410	GC	OR1L6	GC	OST48	OMIM
RLS4	GC	FAM50B	GC	CDG1R	OMIM
IFNGR1	GC	NPY4R	GC	EPHB2	OMIM
DGCR6	GC	DEPTOR	GC	EPHT3	OMIM
MAN2B1	GC	LOC109029533	GC	DRT	OMIM
MVK	GC	RXFP3	GC	ERK	OMIM
CGA	GC	SLC6A15	GC	PCBC	OMIM
XYLT2	GC	SNX10	GC	CAPB	OMIM
PKLR	GC	NEUROG2	GC	BDPLT22	OMIM
TTF2	GC	PHLDA2	GC	DHDDS	OMIM
TBX21	GC	RRIS	GC OMIM	HDS	OMIM
TMEM107	GC	STC1	GC	RP59	OMIM
F7	GC	ENGASE	GC	DEDSM	OMIM
TRIP11	GC	FLG-AS1	GC	SVBP	OMIM
AP3B2	GC	SP100	GC	CCDC23	OMIM
CACNA2D1	GC	BIRC5	GC	NEDAHM	OMIM
MIR20A	GC	THSD4	GC	ALG6	OMIM
MIR342	GC	CNIH1	GC	CDG1C	OMIM
HSPA8	GC	MIR598	GC	PGM1	OMIM
TRPC3	GC	LRRC2-AS1	GC	GSD14	OMIM
COL6A2	GC	PPP1R8	GC	CDG1T	OMIM
SFTPB	GC	SAA1	GC	SARS1	OMIM
ARSG	GC	GPR88	GC	SARS	OMIM
CLCN4	GC	LOC106728418	GC	SERS	OMIM
CHRNA5	GC	OR1C1	GC	NEDMAS	OMIM
PPARA	GC	NATD1	GC	WARS2	OMIM
KCNQ1OT1	GC	ENSG00000212411	GC	NEMMLAS	OMIM
MIR659	GC	EA3	GC	PEX11B	OMIM
CCDC78	GC	EA7	GC	PEX14B	OMIM
GABRG3	GC	EA8	GC	PRUNE1	OMIM
HSPB3	GC	LAT2	GC	DRES17	OMIM
ACY1	GC	EID1	GC	NMIHBA	OMIM
ARFGEF2	GC	CKAP5	GC	ARHGEF2	OMIM
RLS3	GC	DROSHA	GC	GEFH1	OMIM
RLS5	GC	ZMYND10	GC	KIAA0651	OMIM
RLS8	GC	TLNRD1	GC	NEDMHM	OMIM
ABCG8	GC	P2RX5	GC	BDET	OMIM
HOMER1	GC	KRIT1	GC	PEX19 P	OMIM
ABCB6	GC	CDC27	GC	XF	OMIM

PON2	GC	BICC1	GC	HK33	OMIM
GYS2	GC	ENPP3	GC	D1S2223E	OMIM
POMT2	GC	BANK1	GC	PBD12A	OMIM
DDX11	GC	TTC3	GC	UFC1	OMIM
ANTXR2	GC	FGF16	GC	NEDSG	OMIM
PDE5A	GC	DUSP4	GC	NFASC	OMIM
CYP2C9	GC	HBQ1	GC	KIAA0756	OMIM
TUBA4A	GC	ASAH2	GC	NEDCPMD	OMIM
ZDHHC9	GC	UCN	GC	COG2	OMIM
PPP1R1B	GC	KCNJ3	GC	LDLC	OMIM
TNNT1	GC	SLC10A1	GC	CDG2Q	OMIM
TUBB1	GC	MIR361	GC	PPP1CB	OMIM
PLCE1	GC	HMX2	GC	NSLH2	OMIM
ITIH3	GC	DDI2	GC	FBXO11	OMIM
KCNN3	GC	NFE2L1	GC	FBX11	OMIM
CDKN1A	GC	CTNNA1	GC	VIT1	OMIM
ATF6	GC	HK2	GC	PRMT9	OMIM
MSX2	GC	CHST3	GC	IDDFBA	OMIM
MAX	GC	PSMF1	GC	PEX13	OMIM
BGN	GC	ICOS	GC	ZWS	OMIM
PIGC	GC	TIMM23	GC	PBD11A	OMIM
PNOC	GC	SCFD1	GC	PBD11B	OMIM
TBX20	GC	RPS6KA2	GC	MOGS	OMIM
YWHAG	GC	ATP11C	GC	GCS1	OMIM
BRWD3	GC	CD1B	GC	CDG2B	OMIM
FZD4	GC	MIR431	GC	GGCX	OMIM
DLGAP1	GC	MIR539	GC	VKCFD1	OMIM
AGER	GC	INSM2	GC	ADHD5	OMIM
PRKG1	GC	TMEM161B	GC	SMPD4	OMIM
CDH3	GC	DDIT4	GC	NSMASE3	OMIM
MAF	GC	CALHM1	GC	KIAA1418	OMIM
HYDIN	GC	TIAM1	GC	NEDMABA	OMIM
CRHR2	GC	FRS2	GC	CCDC115	OMIM
OPRL1	GC	GZMM	GC	CCP1	OMIM
COL6A3	GC	SLC1A6	GC	CDG2O	OMIM
MED13L	GC	NPC1L1	GC	TBR1 IDDAS	OMIM
CHRM1	GC	GSX1	GC	SCN9A	OMIM
CHD1	GC	MIR10B	GC	NENA	OMIM
TEF	GC	SPTBN1	GC	PN1	OMIM
COX15	GC	CSNK2A2	GC	FEB3B	OMIM
NPHS2	GC	CHRD	GC	GEFSP7	OMIM
SLC45A2	GC	ESRRB	GC	FNP	OMIM
ALDH6A1	GC	TEKT2	GC	HSAN2D	OMIM
DPP4	GC	ZIC4	GC	METTL5	OMIM

DGCR8	GC	ARHGAP26	GC	HSPC133	OMIM
SNURF	GC	MCHR1	GC	MRT72	OMIM
SORL1	GC	SNORD109B	GC	DYNC1I2	OMIM
TRPM4	GC	RSPH6A	GC	DNCI2	OMIM
PTGDS	GC	RB1CC1	GC	IC2	OMIM
RPS23	GC	MC3R	GC	NEDMIBA	OMIM
TFAP2A	GC	SYVN1	GC	LNPK	OMIM
LRP1	GC	CKS1B	GC	KIAA1715	OMIM
ABCA3	GC	RMDN3	GC	NEDEHCC	OMIM
PBX1	GC	ACTR3C	GC	HECW2	OMIM
COQ6	GC	MGRN1	GC	NEDL2	OMIM
ITM2B	GC	PCDH8	GC	KIAA1301	OMIM
IL15	GC	HYAL1	GC	NDHSAL	OMIM
LARGE1	GC	KLRC1	GC	ZNF142	OMIM
ASNS	GC	CACNB3	GC	NEDISHM	OMIM
TMPO	GC	DEGS2	GC	PER2	OMIM
COA8	GC	TBPL1	GC	FASPS1	OMIM
VSX2	GC	ADH5	GC	KIAA0347	OMIM
SDHD	GC	STX10	GC	BRPF1	OMIM
FGF3	GC	RCVRN	GC	BR140	OMIM
COL4A1	GC	TEKT1	GC	IDDDFP	OMIM
MSH2	GC	GANAB	GC	IQSEC1	OMIM
ADH1B	GC	SLC7A10	GC	KIAA0763	OMIM
H19-ICR	GC	HCN2	GC	IDDSSBA	OMIM
ANO10	GC	TCTE1	GC	NGLY1	OMIM
KCNAB2	GC	PCDHB1	GC	PNG1	OMIM
MLH1	GC	C6orf62	GC	CDDG	OMIM
ARNTL2	GC	MIR3176	GC	CDG1V	OMIM
NACC1	GC	PWRN2	GC	STT3B	OMIM
STX16	GC	PPM1K	GC	SIMP	OMIM
ORAI1	GC	MFAP2	GC	CDG1X	OMIM
TBCK	GC	FASTK	GC	PLCD1	OMIM
NFU1	GC	WDR5	GC	NDNC3	OMIM
MASP2	GC	ALOX12	GC	CTNNB1	OMIM
SIX5	GC	SLC4A11	GC	NEDSDV	OMIM
MORC2	GC	LXN	GC	EVR7	OMIM
SP1	GC	AKT1S1	GC	DHX30	OMIM
RBM20	GC	DMAP1	GC	DDX30	OMIM
POLR2A	GC	DOCK4	GC	RETCOR	OMIM
COL25A1	GC	TAOK3	GC	KIAA0890	OMIM
NGFR	GC	GOLGA8F	GC	NEDMIAL	OMIM
GABRB1	GC	GOLGA8G	GC	DOCK3	OMIM
CAMK2B	GC	GOLGA8EP	GC	NEDIDHA	OMIM
ZNF711	GC	SNORD115-2	GC	RFT1	OMIM

SLC13A3	GC	SNORD115-10	GC	CDG1N	OMIM
TRPC6	GC	SNORD115-11	GC	SSD	OMIM
CALB2	GC	SNORD115-12	GC	ZBTB11	OMIM
TWIST2	GC	SNORD115-13	GC	MRT69	OMIM
PDX1	GC	SNORD115-14	GC	P2RY12	OMIM
CDSN	GC	SNORD115-15	GC	P2Y12	OMIM
NLRP12	GC	SNORD115-16	GC	BDPLT8	OMIM
LAMP1	GC	SNORD115-17	GC	RSRC1	OMIM
CYB5A	GC	SNORD115-18	GC	SRRP53	OMIM
BMP1	GC	SNORD115-19	GC	MRT70	OMIM
NAA15	GC	SNORD115-20	GC	AP2M1	OMIM
IRS2	GC	SNORD115-21	GC	CLAPM1	OMIM
GPNMB	GC	SNORD115-22	GC	MRD60	OMIM
PDLIM5	GC	SNORD115-23	GC	ALG3	OMIM
ISCA2	GC	SNORD115-25	GC	NOT56L	OMIM
FCGR3B	GC	SNORD115-26	GC	CDGS4	OMIM
ZNF408	GC	SNORD115-29	GC	CDG1D	OMIM
HLA-DQA2	GC	SNORD115-3	GC	DRD5	OMIM
HSPA4	GC	SNORD115-30	GC	DRD1B	OMIM
ACO1	GC	SNORD115-31	GC	DRD1L2	OMIM
NAIP	GC	SNORD115-32	GC	SRD5A3	OMIM
CDKN1B	GC	SNORD115-33	GC	SRD5A2L	OMIM
MIR30D	GC	SNORD115-34	GC	CDG1Q	OMIM
FGF17	GC	SNORD115-35	GC	KRIZI	OMIM
KCNK4	GC	SNORD115-36	GC	CDG1Q	OMIM
MPP5	GC	SNORD115-37	GC	TMEM165	OMIM
GDF9	GC	SNORD115-38	GC	FT27	OMIM
KISS1	GC	SNORD115-39	GC	CDG2K	OMIM
SNX27	GC	SNORD115-4	GC	SEC31A	OMIM
STAG3	GC	SNORD115-40	GC	SEC31L1	OMIM
ARV1	GC	SNORD115-41	GC	KIAA0905	OMIM
NR1H2	GC	PRR16	GC	NEDSOSB	OMIM
TRMT1	GC	WNT2B	GC	SLC39A8	OMIM
UBE2T	GC	ACY3	GC	BIGM103	OMIM
MMADHC	GC	OPCML	GC	CDG2N	OMIM
LGALS4	GC	DMWD	GC	PAND3	OMIM
ETV6	GC	NNAT	GC	SLC6A19	OMIM
PHACTR1	GC	SPRR3	GC	HND	OMIM
MYO1H	GC	MIR376A1	GC	ADHD4	OMIM
VPS53	GC	SMC5	GC	ZSWIM6	OMIM
HTRA1	GC	CPE	GC	KIAA1577	OMIM
NCAM1	GC	EEF1D	GC	AFND	OMIM
DMGDH	GC	KCNMB3	GC	NEDMAGA	OMIM
WNT10B	GC	KCNQ4	GC	RAD50	OMIM

PDP1	GC	METTL27	GC	NBSLD	OMIM
NUS1	GC	DECR1	GC	PPP2CA	OMIM
LAMA4	GC	NAB2	GC	NEDLBA	OMIM
JPH2	GC	RPL7A	GC	PDGFRB	OMIM
CYFIP1	GC	CCL13	GC	PDGFR	OMIM
MIR143	GC	RABAC1	GC	IBGC4	OMIM
MIR150	GC	SNORD108	GC	IMF1	OMIM
MIR433	GC	SNORD109A	GC	PENTT	OMIM
FCGR2A	GC	SNORD64	GC	KOGS	OMIM
TRIP4	GC	DUSP19	GC	VARS1	OMIM
MIR30B	GC	PHF11	GC	VARS	OMIM
HAVCR2	GC	CRLF2	GC	G7A	OMIM
PDE4B	GC	ATG13	GC	VARS2	OMIM
MTNR1B	GC	CLDN4	GC	NDMSCA	OMIM
BCL2	GC	TEKT4	GC	FKBP5	OMIM
IL16	GC	IAH1	GC	FKBP51	OMIM
CUL4B	GC	NBPF9	GC	PEX6	OMIM
KCNE5	GC	CLDN1	GC	PXAAA1	OMIM
COL4A4	GC	ACCS	GC	PAF2	OMIM
ACTN4	GC	LOC106029311	GC	PBD4A	OMIM
GPR50	GC	LOC106029313	GC	PDB4B	OMIM
FADS1	GC	PRNT	GC	HMLR2	OMIM
HOXA2	GC	ADAMTS14	GC	ADHD3	OMIM
TLR9	GC	ANGPTL6	GC	SLC17A5	OMIM
CCDC103	GC	FASN	GC	SIASD	OMIM
ENPP1	GC	INHBB	GC	SLD	OMIM
SREBF1	GC	DMRT3	GC	SLC35A1	OMIM
MNX1	GC	LRRC75B	GC	CST	OMIM
RSPO4	GC	C22orf15	GC	CDG2F	OMIM
TRPM7	GC	DENND1A	GC	WASF1	OMIM
AIF1	GC	IL23A	GC	WAVE	OMIM
ZEB1	GC	MTHFSD	GC	WAVE1	OMIM
KCTD7	GC	NPHP3-ACAD11	GC	SCAR1	OMIM
PRLR	GC	TREM1	GC	NEDALVS	OMIM
FKBP14	GC	CUL2	GC	NUS1	OMIM
MIR142	GC	CARS1	GC	NGBR	OMIM
MIR320A	GC	GLS2	GC	C6orf68	OMIM
AASS	GC	ASH2L	GC	CDG1AA	OMIM
LRRC56	GC	UCP1	GC	MRD55	OMIM
SNCG	GC	HOXA3	GC	MAFD6	OMIM
CLCNKA	GC	LETM1	GC	BPAD	OMIM
MIR137	GC	CKAP4	GC	PEX7	OMIM
ARIH1	GC	ILK	GC	RCDP1	OMIM
CNOT3	GC	KLK3	GC	PBD9B	OMIM

TUBA8	GC	VPREB1	GC	PEX3	OMIM
MICU1	GC	NOL3	GC	PBD10A	OMIM
CDC6	GC	C5AR1	GC	PBD10B	OMIM
PIGW	GC	BCL7B	GC	FGFR1OP	OMIM
LMBRD1	GC	CDX2	GC	FOP	OMIM
OSTM1	GC	OXA1L	GC	DLL1	OMIM
PPP2R1A	GC	CUL4A	GC	DELTA1	OMIM
KIF5C	GC	GLG1	GC	NEDBAS	OMIM
STOX1	GC	MT-TG	GC	INTS1	OMIM
TAC3	GC	MINK1	GC	INT1	OMIM
DAB1	GC	FABP4	GC	KIAA1440	OMIM
ANKRD1	GC	NCK1	GC	NDCAGF	OMIM
ATP10A	GC	ZFP28	GC	BRAT1	OMIM
MIR133B	GC	CSRNP3	GC	BAAT1	OMIM
VAX1	GC	MAMLD1	GC	C7orf27	OMIM
SRD5A1	GC	WNT11	GC	RMFSL	OMIM
CSN1S1	GC	CDCA5	GC	NEDCAS	OMIM
SLC2A9	GC	TTLL1	GC	WIPI2	OMIM
TBX19	GC	CYTH4	GC	IDDSSA	OMIM
SLC39A8	GC	RPLP0	GC	CDK13	OMIM
PTPRD	GC	EOMES	GC	DC2L5	OMIM
GFM1	GC	MTX1	GC	CHED	OMIM
GTS	GC	NOTUM	GC	CHDFIDD	OMIM
UBQLN4	GC	HOXA11	GC	PEX1	OMIM
VAMP2	GC	GYG2	GC	ZWS1	OMIM
CLPB	GC	RRAGC	GC	PBD1A	OMIM
GLO1	GC	DMTN	GC	PBD1B	OMIM
BCL11A	GC	RGS6	GC	HMLR1	OMIM
GPX4	GC	ADAM12	GC	ACTL6B	OMIM
FARS2	GC	NLRP2	GC	BAF53B	OMIM
NBN	GC	SH2D3C	GC	IDDSSAD	OMIM
KCNJ18	GC	CACNB1	GC	EIEE76	OMIM
CD40	GC	CKB	GC	PUS7	OMIM
MIRLET7D	GC	NAP1L4	GC	KIAA1897	OMIM
XYLT1	GC	IL12RB2	GC	IDDABS	OMIM
SLC17A7	GC	SPATA22	GC	COG5	OMIM
SFTPC	GC	NDRG2	GC	GOLTC1	OMIM
MBTPS1	GC	BNC2	GC	GTC90	OMIM
KLHL7	GC	DOP1B	GC	CDG2I	OMIM
CACNA2D2	GC	PSG3	GC	FOXP2	OMIM
SCN1A-AS1	GC	PTPN5	GC	SPCH1	OMIM
TSEN34	GC	ABALON	GC	TNRC10	OMIM
HNRNPK	GC	GLYAT	GC	CAGH44	OMIM
IL18R1	GC	RPS6KA6	GC	PEX2	OMIM

SECISBP2	GC	RUNX3	GC	PAF1	OMIM
CARS2	GC	NELFCD	GC	PMP35	OMIM
PIGL	GC	THAP7	GC	PBD5A	OMIM
TNNI3K	GC	PAX7	GC	PBD5B	OMIM
FAT4	GC	YPEL1	GC	PXMP3	OMIM
DHPS	GC	ABHD11	GC	OTUD6B	OMIM
SRCAP	GC	GNAT3	GC	DUBA5	OMIM
HRH2	GC	NSUN7	GC	IDDFSDA	OMIM
ALDH4A1	GC	LACTB	GC	INTS8	OMIM
SDHB	GC	KEL	GC	INT8	OMIM
LONP1	GC	WNT9A	GC	NEDCHS	OMIM
BAX	GC	CYP1A1	GC	FZD6	OMIM
AMPD1	GC	PDHB	GC	NDNC10	OMIM
NECAP1	GC	UBR5	GC	PLAA	OMIM
VPS13D	GC	SLC26A7	GC	PLAP	OMIM
STAG1	GC	UTS2R	GC	NDMSBA	OMIM
MIR206	GC	RNF146	GC	B4GALT1	OMIM
NRXN2	GC	MAB21L1	GC	GGTB2	OMIM
CHRNA3	GC	PITPNM2	GC	GT1	OMIM
MSH6	GC	WDR41	GC	GTB	OMIM
ESCO2	GC	ATP2B4	GC	CDG2D	OMIM
CBSL	GC	TMEM189	GC	PRKACG	OMIM
DCAF8	GC	FKBP6	GC	BDPLT19	OMIM
ATG5	GC	FBN3	GC	GPR51	OMIM
TUBG1	GC	TEN1	GC	EIEE59	OMIM
PAEP	GC	RHOA	GC DrugBank	NDPLHS	OMIM
CARD11	GC	NECTIN4	GC	ALG2	OMIM
LOC108663987	GC	SIK1B	GC	CDGII	OMIM
XPNPEP3	GC	CAMK2N2	GC	CMSTA3	OMIM
PORCN	GC	SLC30A1	GC	CMS14	OMIM
PGK1	GC	PLS1	GC	PAND2	OMIM
DPYS	GC	OSR2	GC	DPM2	OMIM
CNTN4	GC	TIMM23B	GC	CDG1U	OMIM
ERCC4	GC	MRT19	GC	DOLK	OMIM
REEP1	GC	MRT23	GC	TMEM15	OMIM
SLC25A3	GC	MRT24	GC	DK1	OMIM
CSF1	GC	MRT25	GC	SEC59	OMIM
CNC2	GC	MRT28	GC	KIAA1094	OMIM
BIN1	GC	CDH4	GC	CDG1M	OMIM
SC5D	GC	PPIAL4D	GC	NTNG2	OMIM
PIGG	GC	LINC00624	GC	NEDBASH	OMIM

NAA10	GC	MIR5087	GC	GFI1B	OMIM
PIK3C2A	GC	ARMC4	GC	BDPLT17	OMIM
PITX1	GC	ACSS1	GC	GRIN1	OMIM
LOXL1	GC	GPC4	GC	NMDAR1	OMIM
CETP	GC	FGR	GC	NDHMSR	OMIM
IAPP	GC	TMEM37	GC	NDHMSD	OMIM
IL6R	GC	MCF2	GC	CACNA1B	OMIM
VIM	GC	MNT	GC	CACNL1A5	OMIM
TMEM132D	GC	WASF3	GC	NEDNEH	OMIM
SLC17A6	GC	RAD21L1	GC	HK1	OMIM
PREP	GC	PPP1R12A	GC	HKD	OMIM
VMA21	GC	MAS1	GC	HMSNR	OMIM
SPRED1	GC	GIP	GC	RP79	OMIM
PSMD4	GC	DUX4	GC	NEDVIBA	OMIM
MMP14	GC	DAND5	GC	PLAU	OMIM
TALDO1	GC	PARD6G-AS1	GC	URK	OMIM
EPHX1	GC	ST3GAL1	GC	QPD	OMIM
HMGCS2	GC	LOC105274310	GC	BDPLT5	OMIM
SLCO1B1	GC	KBTBD4	GC	ZMIZ1	OMIM
SLC6A20	GC	XAGE3	GC	RAI17	OMIM
PHF21A	GC	DEL10Q26	GC	KIAA1224	OMIM
PIGY	GC	CTEPH1	GC	ZIMP10	OMIM
RHOBTB2	GC	PEE2	GC	NEDDFSA	OMIM
UPF3B	GC	PEE3	GC	ADRB1	OMIM
NPRL2	GC	DEL16P13.2	GC	ADRB1R	OMIM
MIR107	GC	MRT16	GC	RHR	OMIM
SNX3	GC	MRT29	GC	FNSS2	OMIM
UBC	GC	MRT30	GC	DEAF1	OMIM
FGFR4	GC	MRT31	GC	SPN	OMIM
PCDH10	GC	MRT33	GC	ZMYND5	OMIM
ATP5F1E	GC	MRT35	GC	MRD24	OMIM
HTR6	GC	DDAH2	GC	FAR1	OMIM
MECOM	GC	SKA2	GC	MLSTD2	OMIM
GSTP1	GC	ACAD8	GC	PFCRD	OMIM
MIR106A	GC	DMXL2	GC	SLC35C1	OMIM
AARS1	GC	RNASE2	GC	FUCT1	OMIM
CYP2E1	GC	CCL1	GC	CDG2C	OMIM
DLG1	GC	CXCL6	GC	PEX16	OMIM
FCGR2B	GC	SLC30A3	GC	PBD8A	OMIM
KIFBP	GC	EPHA6	GC	PBD8B	OMIM
RNASE3	GC	NCKAP5	GC	PHF21A	OMIM
SCZD2	GC	SCD	GC	BHC80	OMIM
WDTC1	GC	P3H3	GC	KIAA1696	OMIM
CXCR3	GC	MIR296	GC	IDDBCS	OMIM

CNR2	GC	MTOR-AS1	GC	TMX2	OMIM
NOTCH4	GC	ULK1	GC	NEDMCMS	OMIM
TSPAN12	GC	VDAC2	GC	RASGRP2	OMIM
NUP85	GC	IL25	GC	CDC25L	OMIM
APOL4	GC	SRRD	GC	CABP4	OMIM
PARK21	GC	CYSLTR2	GC	CRSD	OMIM
CDH2	GC	GPR108	GC	CSNB2B	OMIM
CHGA	GC	ICA1	GC	PAK1	OMIM
ALPP	GC	BMP7	GC	IDDMSSD	OMIM
DBT	GC	LOC100130587	GC	ALG8	OMIM
CYP7A1	GC	ZNF470	GC	CDG1H	OMIM
HTR4	GC	RBMS3	GC	PCLD3	OMIM
EPHB4	GC	NT5C1B	GC	MRE11A	OMIM
NDUFS3	GC	ZNF674	GC	MRE11	OMIM
PES1	GC	SLC25A6	GC	ATLD	OMIM
TFEB	GC	WWP1	GC	GRIA4	OMIM
AXIN2	GC	FZD1	GC	GLUR4	OMIM
SREBF2	GC	TMOD2	GC	NEDSGA	OMIM
POLR3B	GC	DCLK2	GC	ALKBH8	OMIM
PLAGL1	GC	GML	GC	ABH8	OMIM
CRHBP	GC	YIPF7	GC	MRT71	OMIM
APOL2	GC	GEMIN8	GC	ALG9	OMIM
CCKAR	GC	HOXA4	GC	DIBD1	OMIM
CSF2RA	GC	GTF3A	GC	CDG1L	OMIM
FTO	GC	BTF3L4	GC	GIKANIS	OMIM
FANCI	GC	DHRS7C	GC	DDX6	OMIM
IGHE	GC	LOC110121288	GC	HLR2	OMIM
SPAG1	GC	HRH4	GC	IDDILF	OMIM
DNAJC3	GC	MROH7	GC	DPAGT1	OMIM
NIPA2	GC	HP1BP3	GC	DPAGT2	OMIM
PARK10	GC	TMEM132E	GC	DGPT	OMIM
PARK16	GC	SPINK1	GC	CDG1J	OMIM
IGFALS	GC	SLC37A1	GC	CMSTA2	OMIM
CLTC	GC	MXD1	GC	CMS13	OMIM
IKBKB	GC	RRAS2	GC	CBL	OMIM
PARK12	GC	ZNF526	GC	CBL2	OMIM
PTGS1	GC	HPSE2	GC	NSLL	OMIM
ANG	GC	FBXL5	GC	STT3A	OMIM
SCZD1	GC	RBM25	GC	ITM1	OMIM
IFNB1	GC	C4orf45	GC	TMC	OMIM
PPBP	GC	LTA4H	GC	FLI1	OMIM
TEAD4	GC	IL17D	GC	BDPLT21	OMIM
COL4A5	GC	IGF2-AS	GC	PEX5	OMIM
AP4B1	GC	DLC1	GC	PXR1	OMIM

ASTN2	GC	BNIP3	GC	PTS1R	OMIM
IMPA2	GC	PPID	GC	PBD2A	OMIM
CXCL12	GC	CACNG3	GC	PBD2B	OMIM
GABRA6	GC	PRKCE	GC	RCDP5	OMIM
DAD1	GC	WNT5B	GC	KRAS	OMIM
STIL	GC	VEZT	GC	KRAS2	OMIM
ATOH7	GC	BRD3	GC	RASK2	OMIM
IRS4	GC	PRDM5	GC	NS	OMIM
NCOR1	GC	SRA1	GC	CFC2	OMIM
STAC3	GC	PDCD10	GC	RALD	OMIM
FANCL	GC	PPP1R3C	GC	OES	OMIM
PRKCA	GC	SEC14L5	GC	BHLHE41	OMIM
UBXN2B	GC	HEPHL1	GC	BHLHB3	OMIM
IKZF1	GC	GPX2	GC	SHARP1	OMIM
NEUROD1	GC	CDIP1	GC	FNSS1	OMIM
NPAS3	GC	OR3A3	GC	CNOT2	OMIM
REV3L	GC	POM121C	GC	NOT2	OMIM
ELP2	GC	PDCL	GC	IDNADFS	OMIM
ISL1	GC	TSPAN32	GC	TPH2	OMIM
WNT5A	GC	SPIN4	GC	NTPH	OMIM
TRPV1	GC	TKTL1	GC	ADHD7	OMIM
PPM1D	GC	ATG14	GC	CRY1	OMIM
CHRFAM7A	GC	PAPPA2	GC	PHLL1	OMIM
SCZD3	GC	NEUROG1	GC	DSPD	OMIM
MME	GC	RAB3A	GC	DHX37	OMIM
HFM1	GC	GLRX	GC	KIAA1517	OMIM
CCL3	GC	CNTN5	GC	NEDBAVC	OMIM
KATNB1	GC	PLB1	GC	ADHD6	OMIM
ADAMTSL1	GC	SERTAD3	GC	CDK8	OMIM
TNFSF13B	GC	SYBU	GC	IDDHBA	OMIM
FECH	GC	FZD8	GC	COG6	OMIM
TNFSF12	GC	GHITM	GC	COD2	OMIM
MMP20	GC	ATG7	GC	KIAA1134	OMIM
CAV1	GC	WIZ	GC	CDG2L	OMIM
JUN	GC	MEOX2	GC	SHNS	OMIM
CLP1	GC	ENO3	GC	ALG11	OMIM
IFNA2	GC	MACF1	GC	KIAA1266	OMIM
SV2A	GC	LOC109461477	GC	CDG1P	OMIM
CHRNA6	GC	IL31RA	GC	PAND1	OMIM
BMPR1A	GC	KLK1	GC	FBXL3	OMIM
SCZD8	GC	TRIM64	GC	FBXL3A	OMIM
PRDM8	GC	RNR2	GC	FBL3	OMIM
DHFR	GC	BRS3	GC	IDDSFAS	OMIM
PINK1-AS	GC	CCDC196	GC	TRAPPC6B	OMIM

SLC25A12	GC	ASRGL1	GC	TPC6	OMIM
MIR199A1	GC	CCDC154	GC	NEDMEBA	OMIM
DPYSL2	GC	ALOX5AP	GC	MGAT2	OMIM
SCZD7	GC	ZNF569	GC	CDGS2	OMIM
MB	GC	PPIF	GC	CDG2A	OMIM
RPL18	GC	ENPEP	GC	FUT8	OMIM
DHDDS	GC	NISCH	GC	CDGF1	OMIM
SGO1	GC	DUSP13	GC	ACTN1	OMIM
SOST	GC	ESM1	GC	BDPLT15	OMIM
ITCH	GC	LIPE	GC	IRF2BPL	OMIM
MAP2K5	GC	KCNAB1	GC	C14orf4	OMIM
CXCL9	GC	DNAJB13	GC	EAP1	OMIM
CLPX	GC	ZBTB38	GC	NEDAMSS	OMIM
CEP135	GC	ACVR2A	GC	BCL11B	OMIM
NAT2	GC	RENBP	GC	CTIP2	OMIM
PANX1	GC	MRGPRE	GC	IMD49	OMIM
PRKCB	GC	MPDZ	GC	IDDFSTA	OMIM
CD69	GC	SUPT4H1	GC	CCNK	OMIM
UBB	GC	GEMIN2	GC	IDDHDF	OMIM
SCZD6	GC	NEURL1	GC	GNB5	OMIM
KCND2	GC	SEPTIN7	GC	GB5	OMIM
NLRP7	GC	PPP2R3C	GC	IDDCA	OMIM
ERF	GC	TRAIP	GC	LADCI	OMIM
PDE4A	GC	EXOC3L2	GC	RORA	OMIM
GATA2	GC	TOP1	GC	IDDECA	OMIM
SERPINA7	GC	CHTF18	GC	MPI	OMIM
GSC	GC	RPS6KA5	GC	PMI1	OMIM
RELA	GC	NUP42	GC	CDG1B	OMIM
MMP3	GC	UQCRC2	GC	SCAPER	OMIM
TGM2	GC	PCBP4	GC	KIAA1454	OMIM
GABRA4	GC	GTF2IRD2	GC	IDDRP	OMIM
FABP7	GC	KIF4A	GC	MTHFS	OMIM
EFEMP1	GC	BLVRB	GC	NEDMEHM	OMIM
SCZD10	GC	NEK11	GC	ADHD1	OMIM
YWHAQ	GC	MOB2	GC	MAPK8IP3	OMIM
NRG3	GC	CLEC4E	GC	SYD2	OMIM
MIR212	GC	SPNS1	GC	JSAP1	OMIM
ITGB4	GC	DUSP11	GC	JIP3	OMIM
HPS4	GC	GIPR	GC	NEDBA	OMIM
ANXA11	GC	CCN1	GC	ALG1	OMIM
PIP5K1C	GC	SKAP2	GC	HMAT1	OMIM
ACKR1	GC	SF3B6	GC	HMT1 C	OMIM
CHST11	GC	NAV2	GC	DG1K	OMIM
MSR1	GC	LILRA4	GC	PMM2	OMIM

HPS5	GC	IRX6	GC	CDG1A	OMIM
MIR15B	GC	RSPRY1	GC	GRIN2A	OMIM
MIR197	GC	CTSE	GC	NMDAR2A	OMIM
LRPPRC	GC	TNKS2	GC	FESD	OMIM
PIGO	GC	ORMDL3	GC	LKS	OMIM
SET	GC	PLP2	GC	MAFD4	OMIM
MT-TS1	GC	LIMK2	GC	COG7	OMIM
NTNG2	GC	FUT9	GC	CDG2E	OMIM
PTGIS	GC	COL19A1	GC	GNAO1	OMIM
SLC52A1	GC	LOC108228208	GC	EIEE17	OMIM
HTN3	GC	LOC108228209	GC	NEDIM	OMIM
NTRK3	GC	PRKD1	GC	COG8	OMIM
MBD4	GC	BCL6	GC	DOR1	OMIM
CASP9	GC	INTS1	GC	CDG2H	OMIM
CCL17	GC	NUP210	GC	FCSK	OMIM
DGKH	GC	CLEC6A	GC	FUK	OMIM
TRANK1	GC	C14orf178	GC	CDGF2	OMIM
SUGCT	GC	MACROH2A1	GC	COG4	OMIM
APOD	GC	ADAM23	GC	COD1	OMIM
TPMT	GC	AHSA1	GC	CDG2J	OMIM
AFP	GC	SLC29A1	GC	SWILS	OMIM
ZNF462	GC	ICMT	GC	NDNC7	OMIM
MIR198	GC	LNPK	GC	GEMIN4	OMIM
DLGAP3	GC	MYO9B	GC	NEDMCR	OMIM
COL9A1	GC	DBI	GC	POLR2A	OMIM
MT-TW	GC	MYB	GC	RPOL2	OMIM
SLC9A1	GC	SULT1A1	GC	NEDHIB	OMIM
TBC1D7	GC	ST3GAL6	GC	MPDU1	OMIM
ERVW-1	GC	CACNA2D1-AS1	GC	SL15	OMIM
GNAQ	GC	SOX14	GC	CDGIF	OMIM
SETD1A	GC	MTUS2	GC	KDM6B	OMIM
HLA-C	GC	FGFRL1	GC	JMJD3	OMIM
SLC6A14	GC	THBS2	GC	KIAA0346	OMIM
PSMNSW	GC	SLC37A3	GC	NEDCFSA	OMIM
TOR3A	GC	VWC2	GC	VAMP2	OMIM
PAX4	GC	SCNM1	GC	SYB2	OMIM
NQO1	GC	DPYD-AS2	GC	NEDHAHM	OMIM
CHIT1	GC	ASAP1	GC	ADHD2	OMIM
ACTL6B	GC	CAPRIN1	GC	TMEM199	OMIM
SHANK1	GC	C11orf21	GC	VMA12	OMIM
HDAC6	GC	LY6E	GC	VPH2	OMIM
SLC16A1	GC	RFK	GC	C17orf32	OMIM
MGR6	GC	MXD4	GC	CDG2P	OMIM
RAB5A	GC	CHTF8	GC	SLC6A4	OMIM

AHCY	GC	CLDN3	GC	HTT	OMIM
GAST	GC	ZDBF2	GC	OCD1	OMIM
PEMT	GC	GPR1-AS	GC	SLFN14	OMIM
ASPRV1	GC	LINC01090	GC	BDPLT20	OMIM
AVIL	GC	ITLN1	GC	PEX12	OMIM
SP4	GC	CBLL1	GC	PBD3A	OMIM
GFRA1	GC	NEK3	GC	ITGA2B	OMIM
FAM3D	GC	COPE	GC	GP2B	OMIM
TBX4	GC	FDXR	GC	CD41B	OMIM
RARA	GC	SLC29A2	GC	GT	OMIM
PAK3	GC	C9orf50	GC	BDPLT2	OMIM
PON3	GC	GTF2IRD2B	GC	ITGB3	OMIM
HTR5A	GC	DEFB103B	GC	GP3A	OMIM
TLR3	GC	PAICS	GC	MED13	OMIM
LGI4	GC	RBM45	GC	THRAP1	OMIM
MALAT1	GC	ADGRG6	GC	TRAP240	OMIM
CDKN3	GC	ALG10B	GC	MRD61	OMIM
WNT3A	GC	ADCY9	GC	BPTF	OMIM
WASF2	GC	CGB5	GC	FALZ	OMIM
HMGA2	GC	REC8	GC	FAC1	OMIM
IL10RB	GC	TRIM74	GC	NURF301	OMIM
BOLA3	GC	ABHD11-AS1	GC	NEDDFL	OMIM
KCNQ5	GC	TSIX	GC	NDNC9	OMIM
LGALS3	GC	PRIMPOL	GC	COG1	OMIM
GRK3	GC	CADPS	GC	LDLB	OMIM
MIR30A	GC	NCR3	GC	KIAA1381	OMIM
CCL4	GC	DAGLA	GC	CDG2G	OMIM
IER3IP1	GC	PCYOX1	GC	TMEM94	OMIM
LRP8	GC	GTF2IRD2P1	GC	KIAA0195	OMIM
PIP4K2A	GC	PKNOX2	GC	IDDCDF	OMIM
AXIN1	GC	MED7	GC	RAC3	OMIM
PGF	GC	ICAM2	GC	CSNK1D	OMIM
TAF15	GC	NSUN3	GC	ASPS	OMIM
GPAA1	GC	WLS	GC	FASPS2	OMIM
CLEC7A	GC	DNAJC24	GC	WDR45B	OMIM
TLR6	GC	NSUN5P2	GC	WIPI3	OMIM
MGR5	GC	NSUN5P1	GC	WDR45L	OMIM
TXN	GC	CMS1A1	GC	NEDSBAS	OMIM
IGFBP2	GC	IGH	GC	MAFD1	OMIM
DSCAM	GC	SPTLC3	GC	BPAD	OMIM
SLC6A17	GC	ATE1	GC	MD1	OMIM
BDNF-AS	GC	CDC123	GC	OHDS	OMIM
KPTN	GC	CAMSAP2	GC	TBXA2R	OMIM
TYMS	GC	ERAP2	GC	BDPLT13	OMIM

VCAN	GC	ELF3	GC	RAB11B	OMIM
BEST1	GC	MED31	GC	NDAGSCW	OMIM
CD80	GC	TRIM50	GC	DHPS	OMIM
STAT6	GC	SPDYE1	GC	NEDSSWI	OMIM
ITGA3	GC	PROB1	GC	NACC1	OMIM
TMEM230	GC	TMEM270	GC	BTBD14B	OMIM
TCTEX1D2	GC	GTF2IP1	GC	NECFM	OMIM
RPE65	GC	MT-TD	GC	SPTBN4	OMIM
NDEL1	GC	GTF2IP4	GC	QV	OMIM
ACTN1	GC	WBSCR2	GC	NEDHND	OMIM
YWHAE	GC	SNORD133	GC	CMND	OMIM
CRB2	GC	WBSCR23	GC	CNOT3	OMIM
CARD9	GC	PDS5B	GC	NOT3	OMIM
RPL35	GC	IL21	GC	IDDSADF	OMIM
FTH1	GC	NUDC	GC	GP6	OMIM
JPH1	GC	NCOA1	GC	GPIV	OMIM
NAGK	GC	NPY5R	DrugBank	BDPLT11	OMIM
H3R	TTD				

GC: the GeneCards Database; OMIM: the Online Mendelian Inheritance in Man; TTD: the Therapeutic Target Database

Supplementary Table S5. Compound-Target Network information for BXHP

Name	Type	Degree	Betweenness Centrality
kaempferol	mol	47	0.376658
luteolin	mol	40	0.329407
beta-sitosterol	mol	25	0.147799
Stigmasterol	mol	24	0.180146
baicalein	mol	23	0.162184
Cavidine	mol	19	0.119031
coniferin	mol	14	0.058167
morin	mol	10	0.066690
delphinidin	mol	5	0.003864
(3S,6S)-3-(benzyl)-6-(4-hydroxybenzyl)piperazine-2,5-quinone	mol	3	0.001202
Spinasterol	mol	3	0.004765
24-Ethylcholest-4-en-3-one	mol	2	0.000910
gondoic acid	mol	2	0.000184
10,13-eicosadienoic	mol	2	0.000184
Vulgaxanthin-I	mol	2	0.000742
poriferasterol monoglucoside_qt	mol	2	0.000381
stigmast-7-enol	mol	2	0.000381
Cycloartenol	mol	1	0.000000
beta-D-Ribofuranoside, xanthine-9	mol	1	0.000000
Prostaglandin G/H synthase 2	gene	12	0.147901
Nuclear receptor coactivator 2	gene	12	0.106607
Prostaglandin G/H synthase 1	gene	10	0.115612
Progesterone receptor	gene	7	0.035114
Androgen receptor	gene	7	0.038088
Muscarinic acetylcholine receptor M1	gene	5	0.022507
Alpha-1B adrenergic receptor	gene	5	0.022507
Beta-2 adrenergic receptor	gene	5	0.009015
Peroxisome proliferator activated receptor gamma	gene	5	0.018752
Mineralocorticoid receptor	gene	4	0.018119
Muscarinic acetylcholine receptor M3	gene	4	0.006350
Sodium channel protein type 5 subunit alpha	gene	4	0.006350
Caspase-3	gene	4	0.021443
Mu-type opioid receptor	gene	3	0.002425
Trypsin-1	gene	3	0.010638
Nuclear receptor coactivator 1	gene	3	0.007724
Transcription factor p65	gene	3	0.010638
RAC-alpha serine/threonine-protein kinase	gene	3	0.010638
Apoptosis regulator Bcl-2	gene	3	0.008683
Apoptosis regulator BAX	gene	3	0.008683
Muscarinic acetylcholine receptor M2	gene	3	0.008647

Gamma-aminobutyric acid receptor subunit alpha-1	gene	3	0.008647
Transcription factor AP-1	gene	3	0.012077
Nitric oxide synthase, inducible	gene	3	0.002778
Potassium voltage-gated channel subfamily H member 2	gene	2	0.001164
ADRB1	gene	2	0.001889
Alpha-1D adrenergic receptor	gene	2	0.000609
Sodium-dependent serotonin transporter	gene	2	0.001164
Coagulation factor VII	gene	2	0.006212
Vascular endothelial growth factor A	gene	2	0.003345
Matrix metalloproteinase-9	gene	2	0.003345
Aryl hydrocarbon receptor	gene	2	0.003278
Alpha-1A adrenergic receptor	gene	2	0.001178
Caspase-9	gene	2	0.005400
Sodium-dependent noradrenaline transporter	gene	2	0.004807
Tumor necrosis factor	gene	2	0.004015
Interstitial collagenase	gene	2	0.004015
Heme oxygenase 1	gene	2	0.004015
Intercellular adhesion molecule 1	gene	2	0.004015
Arachidonate 5-lipoxygenase	gene	2	0.003709
Glutathione S-transferase P	gene	2	0.004015
Solute carrier family 2, facilitated glucose transporter member 4	gene	2	0.004015
Insulin receptor	gene	2	0.004015
DNA topoisomerase 1	gene	2	0.003353
Muscarinic acetylcholine receptor M5	gene	1	0.000000
5-hydroxytryptamine receptor 3A	gene	1	0.000000
Alpha-2C adrenergic receptor	gene	1	0.000000
Delta-type opioid receptor	gene	1	0.000000
Retinoic acid receptor RXR-beta	gene	1	0.000000
cAMP and cAMP-inhibited cGMP 3',5'-cyclic phosphodiesterase 10A	gene	1	0.000000
Proto-oncogene c-Fos	gene	1	0.000000
Hypoxia-inducible factor 1-alpha	gene	1	0.000000
Myeloperoxidase	gene	1	0.000000
Insulin-like growth factor II	gene	1	0.000000
Cytochrome c	gene	1	0.000000
Arachidonate 12-lipoxygenase, 12S-type	gene	1	0.000000
Nuclear factor of activated T-cells, cytoplasmic 1	gene	1	0.000000
NADPH oxidase 5	gene	1	0.000000
Apolipoprotein D	gene	1	0.000000
Neuronal acetylcholine receptor subunit alpha-2	gene	1	0.000000
Caspase-8	gene	1	0.000000
Protein kinase C alpha type	gene	1	0.000000

Serum paraoxonase/arylesterase 1	gene	1	0.000000
Microtubule-associated protein 2	gene	1	0.000000
Alcohol dehydrogenase 1C	gene	1	0.000000
Alpha-2A adrenergic receptor	gene	1	0.000000
Sodium-dependent dopamine transporter	gene	1	0.000000
Aldose reductase	gene	1	0.000000
Urokinase-type plasminogen activator	gene	1	0.000000
Leukotriene A-4 hydrolase	gene	1	0.000000
Amine oxidase [flavin-containing] B	gene	1	0.000000
Amine oxidase [flavin-containing] A	gene	1	0.000000
Estrogen receptor	gene	1	0.000000
Cyclin-A2	gene	1	0.000000
Acetylcholinesterase	gene	1	0.000000
Inhibitor of nuclear factor kappa-B kinase subunit beta	gene	1	0.000000
Activator of 90 kDa heat shock protein ATPase homolog 1	gene	1	0.000000
Mitogen-activated protein kinase 8	gene	1	0.000000
Signal transducer and activator of transcription 1-alpha/beta	gene	1	0.000000
Cytochrome P450 3A4	gene	1	0.000000
Cytochrome P450 1A2	gene	1	0.000000
Cytochrome P450 1A1	gene	1	0.000000
e-selectin	gene	1	0.000000
Vascular cell adhesion protein 1	gene	1	0.000000
Nuclear receptor subfamily 1 group I member 2	gene	1	0.000000
Cytochrome P450 1B1	gene	1	0.000000
Hyaluronan synthase 2	gene	1	0.000000
26S proteasome non-ATPase regulatory subunit 3	gene	1	0.000000
Nuclear receptor subfamily 1 group I member 3	gene	1	0.000000
Serine/threonine-protein phosphatase 2B catalytic subunit alpha isoform	gene	1	0.000000
Glutathione S-transferase Mu 1	gene	1	0.000000
Epidermal growth factor receptor	gene	1	0.000000
Bcl-2-like protein 1	gene	1	0.000000
Cyclin-dependent kinase inhibitor 1	gene	1	0.000000
72 kDa type IV collagenase	gene	1	0.000000
Mitogen-activated protein kinase 1	gene	1	0.000000
Interleukin-10	gene	1	0.000000
Retinoblastoma-associated protein	gene	1	0.000000
Interleukin-6	gene	1	0.000000
E3 ubiquitin-protein ligase Mdm2	gene	1	0.000000
Amyloid beta A4 protein	gene	1	0.000000
Induced myeloid leukemia cell differentiation protein Mcl-1	gene	1	0.000000

Baculoviral IAP repeat-containing protein 5	gene	1	0.000000
Interleukin-2	gene	1	0.000000
Tyrosinase	gene	1	0.000000
Interferon gamma	gene	1	0.000000
Interleukin-4	gene	1	0.000000
CD40 ligand	gene	1	0.000000
Adenylate cyclase type 2	gene	1	0.000000
Hepatocyte growth factor receptor	gene	1	0.000000
Endothelin-1	gene	1	0.000000
Multidrug resistance protein 1	gene	1	0.000000
Platelet glycoprotein 4	gene	1	0.000000
Glutathione reductase, mitochondrial	gene	1	0.000000

Supplementary Table S6. The core PPI network for 34 Core Candidate BXHP Targets

Target	Degree (DC)	LAC	Eigenvector (EC)	Betweenness (BC)	Closeness (CC)	Network (NC)
AKT1	71	24.197184	0.2037491	1204.3031	0.7151899	60.473476
IL6	64	25.15625	0.19558994	896.1162	0.6890244	51.934406
VEGFA	60	26.633333	0.19526711	439.6876	0.6608187	52.233864
JUN	60	26.7	0.19547728	462.44553	0.6608187	51.671867
CASP3	57	26.070175	0.18780428	540.6267	0.65317917	46.826435
FOS	56	23.357143	0.17146671	882.6748	0.65317917	41.194527
PTGS2	54	26.37037	0.18284132	416.53726	0.6348315	45.540604
MAPK8	54	27.555555	0.18801345	209.65298	0.6348315	45.5019
MAPK1	53	26.37736	0.18181162	242.86472	0.6312849	43.62528
EGFR	52	24.884615	0.17430517	388.63925	0.64204544	41.179848
MMP9	47	27.276596	0.17246202	256.82117	0.6042781	39.87058
ESR1	44	23.136364	0.14979742	228.85316	0.6042781	33.526962
CYCS	44	22.454546	0.1521999	188.10568	0.60106385	32.465523
PPARG	41	20.780487	0.13966197	229.38957	0.591623	28.636946
RELA	40	25	0.15296479	161.15675	0.5885417	30.698633
IL10	40	26.75	0.15591635	61.88223	0.58247423	33.459766
BCL2L1	40	25.85	0.15364026	61.90998	0.58247423	32.838665
MMP2	39	25.589743	0.152548	75.881134	0.57360405	31.331295
HMOX1	39	22.923077	0.14345294	118.42971	0.58247423	28.552681
APP	38	13.842105	0.10482103	705.5535	0.5885417	22.000998
IL4	37	23.72973	0.13989754	172.71529	0.57360405	28.463701
CASP8	37	24.972973	0.14504218	48.84611	0.5707071	30.394432
AR	37	20.486486	0.13001317	155.2597	0.57653064	26.726683
IL2	35	24	0.13826314	47.942364	0.56218904	28.153973
STAT1	34	23.647058	0.13643731	65.17049	0.5594059	26.741438
HIF1A	34	21.470589	0.13097338	87.08679	0.5566502	25.206003
CDKN1A	33	22.484848	0.1283113	65.7505	0.5485437	26.94772
MPO	32	19.625	0.11585885	123.632225	0.5594059	23.912392
AHR	32	16.4375	0.10633193	164.21674	0.5432692	20.806871
PGR	29	17.655172	0.10641885	61.478817	0.5512195	20.787596
MMP1	28	20.785715	0.11575514	54.67552	0.5432692	22.623016
ABCB1	25	11.28	0.07418045	147.25879	0.5458937	14.586952
PLAU	21	13.619047	0.08337217	62.776352	0.51834863	15.349145
GSTP1	21	10.380953	0.061818644	155.68744	0.509009	12.414377

Supplementary Table S7. GO Enrichment Analysis for 34 Core Candidate BXHP Targets

Category	Term	Count	PValue	Rich Factor
Biological process	regulation of apoptosis	24	2.40×10^{-21}	3.93×10^{-18}
	regulation of programmed cell death	24	3.01×10^{-21}	4.92×10^{-18}
	regulation of cell death	24	3.27×10^{-21}	5.35×10^{-18}
	regulation of cell proliferation	19	2.18×10^{-14}	3.58×10^{-11}
	response to organic substance	18	9.82×10^{-14}	1.61×10^{-10}
	regulation of transcription	18	3.15×10^{-5}	5.16×10^{-2}
	positive regulation of cellular biosynthetic process	17	8.19×10^{-13}	1.34×10^{-9}
	positive regulation of biosynthetic process	17	1.02×10^{-12}	1.67×10^{-9}
	positive regulation of macromolecule metabolic process	17	2.47×10^{-11}	4.04×10^{-8}
	regulation of transcription, DNA-dependent	17	9.14×10^{-7}	1.50×10^{-3}
Cell component	organelle lumen	16	3.54×10^{-6}	4.25×10^{-3}
	membrane--enclosed lumen	16	4.54×10^{-6}	5.45×10^{-3}
	intracellular organelle lumen	14	8.32×10^{-5}	9.98×10^{-2}
	extracellular region	13	1.19×10^{-3}	1.42
	cytosol	12	1.25×10^{-4}	1.50×10^{-1}
	nuclear lumen	12	2.71×10^{-4}	3.24×10^{-1}
	extracellular region part	11	4.08×10^{-5}	4.90×10^{-2}
	extracellular space	10	1.79×10^{-5}	2.15×10^{-2}
	nucleoplasm	9	7.43×10^{-4}	8.88×10^{-1}
	cell projection	7	4.96×10^{-3}	5.79
Molecular Function	transition metal ion binding	13	3.57×10^{-2}	3.63×10
	DNA binding	12	2.48×10^{-2}	2.67×10
	transcription factor activity	10	5.44×10^{-4}	6.71×10^{-1}
	transcription regulator activity	10	1.10×10^{-2}	1.28×10
	sequence--specific DNA binding	9	1.07×10^{-4}	1.33×10^{-1}
	protein dimerization activity	8	3.52×10^{-4}	4.35×10^{-1}
	lipid binding	7	8.38×10^{-4}	1.03
	peptidase activity	7	2.90×10^{-3}	3.54
	identical protein binding	7	4.97×10^{-3}	5.98
	endopeptidase activity	6	2.39×10^{-3}	2.91

Supplementary Table S8. Pathway Enrichment Analysis for 34 Core Candidate BXHP Targets

Number	Term	Count	PValue	Rich Factor
hsa05200	Pathways in cancer	23	1.17×10^{-20}	1.24×10^{-17}
hsa04080	Toll-like receptor signaling pathway	9	7.48×10^{-8}	7.95×10^{-5}
hsa04020	Pancreatic cancer	8	1.33×10^{-7}	1.42×10^{-4}
hsa05210	Colorectal cancer	8	3.90×10^{-7}	4.15×10^{-4}
hsa05215	T cell receptor signaling pathway	8	2.18×10^{-6}	2.32×10^{-3}
hsa04660	MAPK signaling pathway	8	7.29×10^{-4}	7.73×10^{-1}
hsa05212	Bladder cancer	7	1.12×10^{-7}	1.19×10^{-4}
hsa05222	Prostate cancer	7	1.03×10^{-5}	1.09×10^{-2}
hsa04210	Jak-STAT signaling pathway	7	2.36×10^{-4}	2.50×10^{-1}
hsa04010	Apoptosis	6	1.33×10^{-4}	1.41×10^{-1}
hsa04012	ErbB signaling pathway	6	1.33×10^{-4}	1.41×10^{-1}
hsa04510	Focal adhesion	6	5.84×10^{-3}	6.03
hsa04060	Cytokine-cytokine receptor interaction	6	1.73×10^{-2}	1.69×10
hsa04621	NOD-like receptor signaling pathway	5	4.33×10^{-4}	4.60×10^{-1}
hsa05120	Epithelial cell signaling in Helicobacter pylori infection	5	6.17×10^{-4}	6.54×10^{-1}
hsa05211	Renal cell carcinoma	5	6.89×10^{-4}	7.30×10^{-1}
hsa05220	Chronic myeloid leukemia	5	8.95×10^{-4}	9.47×10^{-1}
hsa04662	B cell receptor signaling pathway	5	8.95×10^{-4}	9.47×10^{-1}
hsa05222	Small cell lung cancer	5	1.37×10^{-3}	1.45
hsa04912	GnRH signaling pathway	5	2.42×10^{-3}	2.54
hsa04722	Neurotrophin signaling pathway	5	5.65×10^{-3}	5.85
hsa05010	Alzheimer's disease	5	1.46×10^{-2}	1.44×10
hsa04672	Intestinal immune network for IgA production	4	2.84×10^{-3}	2.98
hsa04150	mTOR signaling pathway	4	3.37×10^{-3}	3.53
hsa05214	Glioma	4	5.80×10^{-3}	5.99
hsa04115	p53 signaling pathway	4	7.17×10^{-3}	7.37
hsa05218	Melanoma	4	8.09×10^{-3}	8.27
hsa04370	VEGF signaling pathway	4	9.40×10^{-3}	9.55
hsa04664	Fc epsilon RI signaling pathway	4	1.05×10^{-2}	1.06×10
hsa04914	Progesterone-mediated oocyte maturation	4	1.36×10^{-2}	1.36×10
hsa05330	Allograft rejection	3	1.87×10^{-2}	1.82×10
hsa05320	Autoimmune thyroid disease	3	3.59×10^{-2}	3.22×10
hsa05213	Endometrial cancer	3	3.72×10^{-2}	3.31×10
hsa05014	Amyotrophic lateral sclerosis (ALS)	3	3.85×10^{-2}	3.41×10
hsa05223	Non-small cell lung cancer	3	3.98×10^{-2}	3.51×10
hsa05221	Acute myeloid leukemia	3	4.53×10^{-2}	3.89×10
